# Whole-exome sequencing identifies novel protein-altering variants associated with serum apolipoprotein and lipid concentrations

**DOI:** 10.1186/s13073-022-01135-6

**Published:** 2022-11-23

**Authors:** Niina Sandholm, Ronja Hotakainen, Jani K. Haukka, Fanny Jansson Sigfrids, Emma H. Dahlström, Anni A. Antikainen, Erkka Valo, Anna Syreeni, Elina Kilpeläinen, Anastasia Kytölä, Aarno Palotie, Valma Harjutsalo, Carol Forsblom, Per-Henrik Groop, on behalf of the FinnDiane Study Group

**Affiliations:** 1 Folkhälsan Research Center, Biomedicum Helsinki, Haartmaninkatu 8, Helsinki, 00290 Finland; 2 Department of Nephrology, University of Helsinki and Helsinki University Hospital, Helsinki, Finland; 3 Research Program for Clinical and Molecular Metabolism, Faculty of Medicine, University of Helsinki, Helsinki, Finland; 4 Institute for Molecular Medicine Finland (FIMM), HiLIFE, University of Helsinki, Helsinki, Finland; 5 Analytic and Translational Genetics Unit, Department of Medicine, Department of Neurology and Department of Psychiatry, Massachusetts General Hospital, Boston, MA USA; 6 The Stanley Center for Psychiatric Research and Program in Medical and Population Genetics, The Broad Institute of MIT and Harvard, Cambridge, MA USA; 7 Department of Diabetes, Central Clinical School, Monash University, Melbourne, Victoria Australia

**Keywords:** Apolipoprotein A1, Apolipoprotein C-III, Whole-exome sequencing, Lipidomics, *LIPC*, *APOB*, *RBM47*, *GTF3C5*, *MARCHF10*, *RYR3*

## Abstract

**Background:**

Dyslipidemia is a major risk factor for cardiovascular disease, and diabetes impacts the lipid metabolism through multiple pathways. In addition to the standard lipid measurements, apolipoprotein concentrations provide added awareness of the burden of circulating lipoproteins. While common genetic variants modestly affect the serum lipid concentrations, rare genetic mutations can cause monogenic forms of hypercholesterolemia and other genetic disorders of lipid metabolism. We aimed to identify low-frequency protein-altering variants (PAVs) affecting lipoprotein and lipid traits.

**Methods:**

We analyzed whole-exome (WES) and whole-genome sequencing (WGS) data of 481 and 474 individuals with type 1 diabetes, respectively. The phenotypic data consisted of 79 serum lipid and apolipoprotein phenotypes obtained with clinical laboratory measurements and nuclear magnetic resonance spectroscopy.

**Results:**

The single-variant analysis identified an association between the *LIPC* p.Thr405Met (rs113298164) and serum apolipoprotein A1 concentrations (*p*=7.8×10^−8^). The burden of PAVs was significantly associated with lipid phenotypes in *LIPC*, *RBM47*, *TRMT5*, *GTF3C5*, *MARCHF10*, and *RYR3* (*p*<2.9×10^−6^). The *RBM47* gene is required for apolipoprotein B post-translational modifications, and in our data, the association between *RBM47* and apolipoprotein C-III concentrations was due to a rare 21 base pair p.Ala496-Ala502 deletion; in replication, the burden of rare deleterious variants in *RBM47* was associated with lower triglyceride concentrations in WES of >170,000 individuals from multiple ancestries (*p*=0.0013). Two PAVs in *GTF3C5* were highly enriched in the Finnish population and associated with cardiovascular phenotypes in the general population. In the previously known *APOB* gene, we identified novel associations at two protein-truncating variants resulting in lower serum non-HDL cholesterol (*p*=4.8×10^−4^), apolipoprotein B (*p*=5.6×10^−4^), and LDL cholesterol (*p*=9.5×10^−4^) concentrations.

**Conclusions:**

We identified lipid and apolipoprotein-associated variants in the previously known *LIPC* and *APOB* genes, as well as PAVs in *GTF3C5* associated with LDLC, and in *RBM47* associated with apolipoprotein C-III concentrations, implicated as an independent CVD risk factor. Identification of rare loss-of-function variants has previously revealed genes that can be targeted to prevent CVD, such as the LDL cholesterol-lowering loss-of-function variants in the *PCSK9* gene. Thus, this study suggests novel putative therapeutic targets for the prevention of CVD.

**Supplementary Information:**

The online version contains supplementary material available at 10.1186/s13073-022-01135-6.

## Background

Cardiovascular disease (CVD) is the leading cause of mortality worldwide [1]. Blood lipid concentrations are key CVD risk factors, and thus, lipid-lowering medication is an essential treatment option to prevent CVD. Diabetes is another major risk factor for CVD, as over 500 million individuals worldwide have diabetes. In particular, individuals with type 1 diabetes develop CVD early and carry a considerable CVD risk burden, with a 7.5-fold incidence ratio for coronary artery disease (CAD) *vs.* the general population; in the presence of other comorbidities such as diabetic kidney disease (DKD), this ratio is up to 27-fold [2]. This risk is not fully explained by hyperglycemia, but diabetic dyslipidemia is an established risk factor for CVD in these individuals. While hypertriglyceridemia is considered the key characteristic of diabetic dyslipidemia [3], the incidence of CAD increases already below the currently recommended triglyceride cutoff of 1.7 mmol/L, suggesting that the additional risk imposed by lipids is pronounced in diabetes [4].

Genetic factors explain approximately 10–54% of plasma lipid concentrations [5], and the largest genome-wide association study (GWAS) on plasma lipid values identified nearly 400 genetic loci associated with plasma low-density lipoprotein cholesterol (LDLC), triglycerides, total cholesterol, or high-density lipoprotein cholesterol (HDLC) [6]. GWAS studies on lipids focusing on the exonic regions of the genome have identified low-frequency or rare protein-altering variants (PAVs) that contribute to the previously observed common variant lipid associations or even explain most of the associations observed for those [7, 8]. Similarly, a whole-exome sequencing (WES) of 3994 health traits in 454,787 individuals from the UK Biobank indicated that rare variant associations were enriched in loci from GWAS, but were independent of common variant signals [9]. Low-frequency PAVs can have a much stronger impact on the phenotype than the disease-associated common genetic variants, which are enriched for gene regulatory variants and often have moderate effect sizes [10]. It is of note that we have previously used WES to search for low-frequency and rare variants for DKD in individuals with type 1 diabetes [11, 12]. A recent exome sequencing of >170,000 individuals identified rare coding variants in 35 genes for total cholesterol, LDLC, HDLC, triglycerides, or their ratios [13]. Indeed, identification of rare loss-of-function variants may reveal genes that can be targeted to prevent disease, such as the LDLC-lowering loss-of-function variants in *PCSK9*, the identification of which resulted in the PCSK9 inhibitors for preventing CVD [14].

However, previous studies on PAVs for lipid traits were either limited to exome-focused genotyping arrays [8], individuals with suspected monogenic dyslipidemias [15], or simple clinical lipid measurements, e.g., total cholesterol, HDLC, and LDLC [9, 13, 16]. Lipidomic profiles consisting of more detailed lipid and lipoprotein subtypes can increase our understanding of the complex lipidomic regulatory networks and, occasionally, outperform the traditional lipid variables in risk prediction [17]. In addition, apolipoprotein concentrations provide added awareness of the burden of circulating lipoproteins. For example, one apolipoprotein B (apoB) molecule is embedded in each very-low-density lipoprotein (VLDL), intermediate-density lipoprotein (IDL), low-density lipoprotein (LDL), and lipoprotein(a) (Lp[a]) particle and apoB seems to estimate the atherogenic risk more accurately than the traditional LDLC [18] or even multivariable data-driven sub-grouping of lipoprotein subtypes [19]. Furthermore, apolipoprotein C-III (apoC-III)—found particularly in the triglyceride-rich lipoproteins (TRLs)—has been recently implicated as a CVD risk factor both in the general population and in individuals with type 1 diabetes [20, 21]. Genetic studies of these refined lipid phenotypes have revealed common variants contributing, e.g., to apoB concentrations [22], but also identified rare genetic factors with high impact, e.g., on apoC-III concentrations, reflected on the CVD risk [23].

In diabetes, high glucose, insulin, and insulin resistance can affect the lipid metabolism: for example, the apoC-III encoding *APOC3* gene expression is decreased by insulin [24] and stimulated by glucose [25]. Insulin resistance leads to overproduction of large VLDL particles, resulting in elevated triglyceride concentrations [26]. In adipose tissues, insulin suppresses lipolysis leading to mobilization of free fatty acids from stored triglycerides; in the liver, insulin inhibits the transfer of triglycerides to apoB, resulting in an overproduction of VLDL in insulin-resistant states [3].

Genetic studies on lipids in diabetes are of particular importance given the important role of glucose, insulin resistance, and insulin itself, as well as the altered lipid metabolism and exacerbated cardiovascular risk in diabetic dyslipidemia. Notably, only a few studies exist addressing PAVs for lipid traits in the general population and only for the standard clinical lipids. Furthermore, there are no such studies in individuals with type 2 or type 1 diabetes, traits with conspicuously altered lipid metabolism. Combined with a wider range of lipid and lipoprotein distribution among individuals with diabetes, genetic studies on lipid and lipoprotein traits can yield novel discoveries for PAVs that may be generalized also to the general population. Finally, the Finnish population provides advantages and increased statistical power for studying rare variants, as some deleterious rare variants are present at higher frequencies in Finnish subjects due to population isolation and recent genetic bottlenecks [27]. Therefore, using whole-exome and whole-genome sequencing (WES and WGS, respectively), we aimed to identify novel PAVs and protein-truncating variants (PTVs, as putative loss-of-function variants) affecting serum lipid and lipoprotein measurements, complemented with serum nuclear magnetic resonance (NMR) measurements in Finnish individuals with type 1 diabetes in the Finnish Diabetic Nephropathy (FinnDiane) Study [28, 29].

## Methods

### Cohort description

The Finnish Diabetic Nephropathy Study (FinnDiane) is an ongoing nationwide prospective multicenter study consisting of 93 participating centers, established in 1997 to pinpoint risk factors for long-term diabetic complications [28, 29]. In these centers, all adult individuals with type 1 diabetes were invited to participate in the study during the active recruitment period. The study currently includes over 8000 Finnish individuals with type 1 diabetes. The clinical characterization of the participants and the recruitment has been described earlier [29]. In brief, data on diabetic complications, history of cardiovascular event(s), and prescribed medications were registered using standardized questionnaires, and blood and urine samples were collected during a standard visit to the attending physician. DNA was extracted from blood. WES data were available for 481 participants [11], and WGS was performed for 598 participants, non-overlapping with the WES individuals. Furthermore, the study includes GWAS data for 6449 participants [30, 31] overlapping with the individuals with WES or WGS; the non-overlapping GWAS participants were used for replication of the lead findings from WES and WGS.

### Study design

**Fig. 1:**
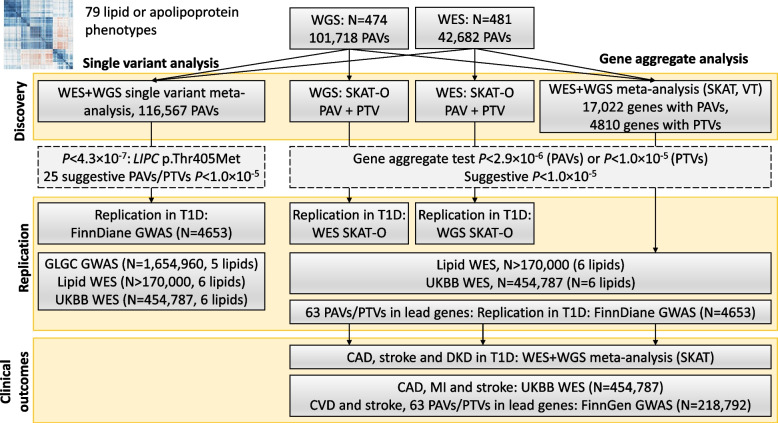
Flowchart of the study design. PTVs: protein-truncating variants, i.e., exon loss, frameshift, stop or start gained or lost, splice acceptor, and donor variants. PAVs: protein-altering variants, defined as PTV plus missense variants, inframe insertions, and deletions. T1D, type 1 diabetes. GLGC: Global Lipids Genetics Consortium. UKBB: UK Biobank. CAD, coronary artery disease. MI, myocardial infarction

We examined the exon content of WES and WGS data available for 481 and 474 FinnDiane participants with type 1 diabetes, respectively, in order to identify low-frequency and rare PAVs and PTVs associated with lipid and lipoprotein measurements (Fig. 1). Replication was sought in the GWAS data for additional FinnDiane participants with the same lipid variables [30], and using the available eight standard lipid phenotypes from the Global Lipids Genetics Consortium (GLGC) GWAS results for 1,654,960 individuals [32], from lipid exome sequencing of >170,000 individuals [13], and exome sequencing of ~450,000 UK Biobank participants [9]. Association with cardiometabolic endpoints were queried in the Finnish general population GWAS data from the FinnGen study [33] and in the UK Biobank exome sequencing data [9].

### Phenotypes

Type 1 diabetes was defined as an onset of diabetes before the age of 40 and the initiation of permanent insulin treatment during the first year after diagnosis. Among the 955 WES and WGS participants, 51% were men, mean age was 45.2 (standard deviation [sd] 10.5) years, and mean diabetes duration was 32.0 (sd 8.71) years (Additional file 1: Table S1).

Serum lipid and apolipoprotein concentrations were determined at the central research laboratory (CL) of Helsinki University Hospital, Finland [34], with more detailed methods in Additional file 1: Table S2.

Proton NMR spectroscopy was utilized to quantify numerous lipoprotein subclasses and their contents along with several metabolites from the serum of 3544 FinnDiane participants at the University of Eastern Finland (Kuopio, Finland) as detailed earlier [35]. Lipoproteins were classified according to their diameter into VLDL, IDL, LDL, and HDL particles. These were further subdivided as described earlier [36]. The spectroscopy was tailored to target three molecular windows: lipoprotein lipids, low molecular weight compounds [37], and serum lipid extracts [38]. The method has been shown to result in consistent lipid–gene associations [39], and many of these measures have been validated by a related NMR biomarker profiling platform developed by the commercial successor of the University of Eastern Finland NMR laboratory, Nightingale Health Plc [40, 41]. The NMR spectroscopy was performed in four different batches. We included in the study 65 NMR lipid phenotypes available for ≥400 individuals with WES or WGS data (Additional file 1: Table S2).

Lipid-lowering medication, defined as the use of statins, was accounted for by using a similar approach previously adopted by others [8, 42]. We divided total cholesterol by 0.8 to account for the 20% reduction in serum total cholesterol induced by statins [43]. We used this adjusted value to calculate LDLC with the Friedewald formula [44]. We divided the subgroups of NMR-measured LDLC by 0.7 to account for a 30% reduction in LDLC. As statins also affect the VLDL particles, the NMR VLDL cholesterol measurements were divided by 0.8 [45]. We left serum triglyceride and HDLC measurements unadjusted, as heritability estimates do not significantly improve when adjusting for statin use [46]. All other lipid variables were left unadjusted, as the exact effect of statins remains unclear.

We performed principal component analysis (PCA) on the 79 lipid and lipoprotein traits with FactoMineR v2.4 R package [47] after imputing the missing values with missMDA v1.18 R package [48] and estimated the number of independent phenotypes based on the eigenvalues.

The diagnosis of CAD was based on data from Statistics Finland and the National Care Register for Health Care using the ICD-10 codes I21, I22, and I23 for myocardial infarction, and the Nordic Classification of Surgical Procedure codes for coronary bypass surgery or coronary balloon angioplasty [49]. The kidney status was based on albuminuria status, and subjects were classified as having normal albumin excretion rate (AER <20 μg/min), microalbuminuria (20–199 μg/min), macroalbuminuria (≥200 μg/min), or renal failure requiring dialysis or kidney transplant.

### Whole-exome and whole-genome sequencing data

The WES study design was initially optimized for DKD, such that half of the individuals had normal AER despite long (≥32 years) diabetes duration, half had severe DKD, i.e., macroalbuminuria and/or renal failure at the end of the follow-up. The sequencing process, variant calling, annotation, and quality control have been described earlier [11, 12]. In brief, sequencing was performed with Illumina HiSeq2000 platform at the University of Oxford, UK, with an average requirement of 20× target capture with an above 80% coverage, resulting in mean sequencing depth of 54.97 bases per position. Variant calling was performed with Genome analysis toolkit (GATK) v2.1 [50], with human genome assembly GRCh37 as reference. Variants were updated to the GRCh38 assembly using the UCSC liftOver tool [51] with default parameters and a hg19 to hg38 chain file.

Similar to WES, the WGS data included 292 controls with normal AER and long diabetes duration (≥35 years) and 291 cases with severe DKD at the end of the follow-up. The sequencing was performed using an Illumina HiSeq X platform (Macrogen Inc., Rockville, MD, USA). Variant calling was done using Broad Institute’s best practices guidelines with GATK v4 [52]. The human genome assembly GRCh38 was used as reference. Variants were filtered to those with variant call rate >98% and in Hardy Weinberg equilibrium (HWE; *p*-value >10^−10^, or >10^−50^ in HLA region, as all had type 1 diabetes). The final data included 21.92 million variants. A total of 573 samples passed the quality control filters, including the percentage of mapped de-duplicated reads and excess heterozygosity. Principal component analysis indicated no population outliers. Lipid-related phenotypes were available for 474 individuals.

All WGS and WES variants were annotated for their functional effects with the SnpEff v4.3 [53] and GrCh38.86 database. Variants classified by SnpEff as PTV (exon loss, frameshift, stop or start gained or lost, splice acceptor, and donor variants) and PAVs (PTV plus missense variants, and inframe insertions or deletions) were included in the analyses.

### Single-variant analysis for WES and WGS variants

All PAVs were tested for association with the lipid and apolipoprotein phenotypes, separately for WES and WGS data sets, using the Rvtests v. (2019-02-09) score test [54]. Analyses were adjusted for sex, age, and the two first genetic principal components. The NMR-measured phenotypes were additionally adjusted for the NMR measurement batch. Inverse normal transformation was performed for all trait residuals. Finally, single-variant meta-analysis of WES and WGS cohorts was performed with RAREMETAL [55] (Fig. 1). Exome-wide significance was defined as *p*<4.3×10^−7^, adjusted for 116,567 tested variants (Bonferroni correction for multiple testing with *α*=0.05 significance level). *P*-values < 1×10^−5^ were considered suggestive. Detailed single-variant statistical analyses and plotting, including survival models for CVD phenotypes, were performed in R using the survival package [56]. Power calculations were performed with R genpwr package [57] for lipid associations, and with R survSNP [58] v0.25 for survival analysis.

We used Sanger sequencing to confirm the 21bp deletion in the *RBM47* gene in seven heterozygotes with lipid data. We designed the primers with Primer3 software [59] and ordered them from Sigma-Aldrich Company Ltd (Haverhill, UK), and sequencing was performed at FIMM (Institute for Molecular Medicine Finland, Helsinki, Finland).

### Single-variant replication

Variants with a *P*-value <1×10^−5^ from the single-variant meta-analysis were chosen for replication in the FinnDiane GWAS data with 6449 individuals, genotyped with Illumina HumanCoreExome Bead arrays, genotypes called with zCall algorithm [60], and initial quality control performed at the University of Virginia [31]. Genotyping data were lifted over to build version 38 (GRCh38/hg38), and data from the four genotyping batches were merged. In sample-wise quality control, individuals with high genotype missingness (>5%), excess heterozygosity (±4 standard deviations), and non-Finnish ancestry (none) were removed. In variant-wise quality control, variants with high missingness (>2%), low HWE *p*-value (<10^−6^), or minor allele count (MAC) <3 were removed. Chip genotyped samples were pre-phased with Eagle 2.3.5 [61], and genotype imputation was performed with Beagle 4.1 (version 08Jun17.d8b) [62] based on the population-specific SISu v3 imputation reference panel with WGS data for 3775 Finnish individuals [63]; only variants with good imputation quality of *r*^*2*^>0.8 were included. Depending on the phenotype, data were available for up to 4653 individuals for total cholesterol after excluding the FinnDiane WES and WGS individuals to ensure independent replication. Rvtests software [54] was used, and analyses with score test were adjusted for sex, age, and the kinship matrix.

Furthermore, replication was sought in three additional general population data sets with a total of eight lipid phenotypes available: The GLGC consortium GWAS data [32] (total cholesterol, HDLC, LDLC, triglycerides, and non-HDLC), UK Biobank WES of 3994 health traits in 454,787 individuals [9] (total cholesterol, HDLC, LDLC, triglycerides, apolipoprotein A, apoB), and lipid WES [13] (total cholesterol, HDLC, LDLC, triglycerides, TG-to-HDLC ratio, and non-HDLC).

### WES and WGS gene-based analysis

Gene-based tests were performed for WES and WGS data using the optimized sequence kernel association test (SKAT-O) [64]. We analyzed the burden of PAVs or PTVs with a minor allele frequency (MAF) < 5% using Rvtests [54] --kernel skato option. Analyses were adjusted for age, sex, and two genetic principal components. NMR phenotypes were further adjusted for the measurement batch. Statistical significance for the burden of PAVs and PTVs were defined as 2.9×10^−6^ and 1.0×10^−5^, respectively (adjusted for up to 17,022 genes with PAVs, and 4810 genes with PTVs in the WES-WGS meta-analysis; Bonferroni correction with *α*=0.05). Significant WES SKAT-O results were internally replicated with WGS SKAT-O results, and vice versa (Fig. 1). Replication was defined as *P*<0.05.

Meta-analysis of the gene-based enrichment of PAVs and PTVs in WES and WGS data was performed with SKAT [65] and variant threshold (VT) tests implemented in RAREMETAL [55] based on the single-variant score test results (described above) and covariance matrices from Rvtests [54]. The pooled variants were re-annotated with the anno tool in RAREMETAL before analysis. Again, variants were limited to those with MAF <5% and analyzed for all PAVs, or PTV variants only. In addition, gene aggregate findings were limited to genes with a cumulative minor allele count (CMAC) of ≥5 (i.e., total aggregated number of the minor allele counts of the eligible variants in a gene; 12,686 genes with PAVs with MAF<5% and CMAC ≥5; and 1418 genes with PTVs with MAF<5% and CMAC ≥5). A significant burden of PAVs or PTVs was defined with the same thresholds as for WES and WGS SKAT-O analysis.

For *CYP3A43*, single-variant and SKAT gene aggregate test meta-analysis were performed similarly with Rvtests [54] and RAREMETAL [55], stratified by the use of statins.

### Replication of gene aggregate findings

Replication for gene aggregate findings was sought from the UK Biobank WES [9] and lipid WES [13] utilized also for the single-variant replication. For UK Biobank, we selected the tests including predicted deleterious PAVs and the putative loss-of-function variant of 1% (M1.1 and M3.1); for the lipid WES, we used the BURDEN and SKAT test results for deleterious PAVs of <1%. We further tested replication of the single variants within the gene aggregate findings using the FinnDiane GWAS data of non-overlapping individuals, similar to the single-variant replication described above.

### Gene-level association with cardiovascular endpoints

The lead genes were tested for association with any DKD (micro- or macroalbuminuria or renal failure vs. normal AER), severe DKD (macroalbuminuria or renal failure vs. normal AER), renal failure vs normal AER, and CVD in the FinnDiane WES + WGS data with SKAT meta-analysis implemented with Rvtests [54] and RAREMETAL [55] similar to the lipid phenotypes. Furthermore, gene aggregate associations with cardiovascular endpoints (CAD, myocardial infarction, stroke, hyperlipidemia) were queried from the UK Biobank WES data [9]. For the identified PAVs in the lead genes, we sought for variant associations with cardiovascular endpoints in the FinnGen study GWAS results for stroke (two definitions), CVD, hypertension, and statin medication phenotypes constructed from ICD codes for 218,792 individuals (release 5) [33]. Wider search was performed based on all 109 “Diseases of the circulatory system” phenotypes for 176,899 Finnish individuals (freeze 4, accessed 11 March 2021; freeze r7 for the VT lead genes *RYR3* and *MARCHF10*, accessed 27 June 2022). Variant enrichment estimates in the Finnish population vs. the gnomAD non-Finnish-non-Estonian European samples were available in the same data.

### Functional annotation

Ensembl Variant Effect Predictor [66] was used to predict the effect of the identified variants, based on SIFT [67] and PolyPhen-2 [68] scoring. Gene expression in various tissues was used to annotate identified genes and studied in the Human Protein Atlas [69].

## Results

The WES and WGS data included 42,682 and 101,718 PAVs, respectively, available for participants with lipid data (Additional file 1: Table S3); 79–82% were low-frequency variants with MAF<5%. A total of 2240 and 9577 variants in WES and WGS, respectively, were annotated as PTV likely to disrupt the protein structure; defined here as frameshift, stop or start gained or lost, exon loss, or splice site acceptor and donor variants. The vast majority, 82–90% of the PTVs, had MAF<5%. For the standard lipid measurements (*N*~920), the effect size required for 80% statistical power to obtain an exome-wide significant *p*-value of <4.3×10^−7^ for a variant with a MAF of 5%, 1%, or 0.1% was of 0.62 standard deviations (sd), 1.37 sd, and 4.31 sd on the lipid distribution, respectively (Additional file 1: Fig. S1). The studied lipid values were correlated with each other (Additional file 1: Fig. S2), and principal component analysis suggested that 12 components were sufficient to explain 95% of the phenotypic variance.

### Single-variant association analysis

**Fig. 2:**
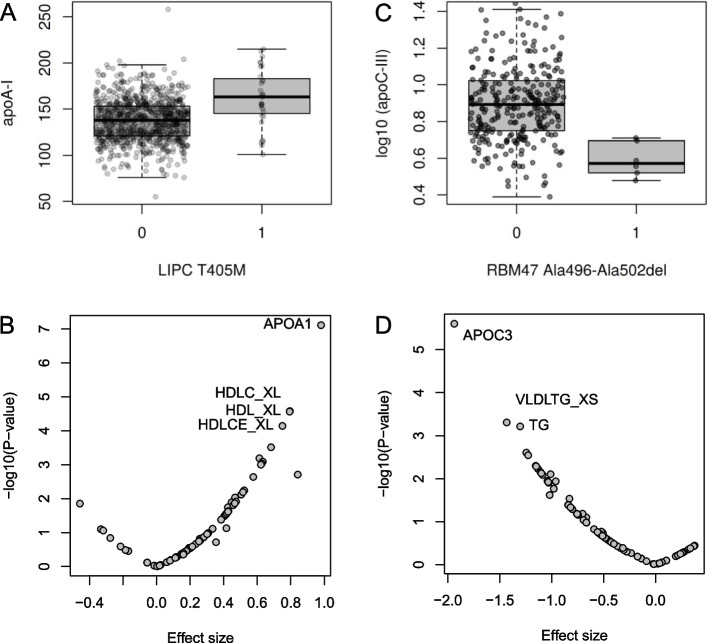
Rare variants in *LIPC* and *RBM47* are associated with serum apoA1 and apoC-III concentrations, respectively. **A ***LIPC* p.Thr405Met (rs113298164) is associated with higher apoA1 (*p*=7.8×10^−8^; multivariable ANOVA *p*= 1.46×10^−9^; *N*=887 carriers, 31 non-carriers). Group number in **A** and **C** indicates the number of rare variants, i.e., 0 refers to non-carriers, 1 refers to heterozygous variant carriers. **B ***LIPC* p.Thr405Met associations across all studied phenotypes. **C** Serum apoC-III concentrations are reduced in the *RBM47* p.Ala496-Ala502del (rs564837143) carriers (*p*=2.49×10^−6^, multivariable ANOVA *p*=2.92×10^−4^; *N* = 288 non-carriers, 6 carriers). **D ***RBM47* p.Ala496-Ala502del associations across all studied phenotypes

In the WES-WGS meta-analysis, a missense variant rs113298164 (p.Thr405Met, MAF 1.7%) in the *LIPC* gene was associated with higher serum apolipoprotein A1 (apoA1) concentrations (*p*=7.8×10^−8^; Table 1, Fig. 2A). In p.Thr405Met carriers (*n*=31), the median serum apoA1 was 163 mg/dl (inter-quartile range [IQR] 145–183) mg/dl, vs. 138 (IQR 121–153) mg/dl in the non-carriers (multivariable ANOVA *p*=1.46×10^−9^). In Cox proportional-hazard models, p.Thr405Met was not associated with CAD, nor with stroke (Additional file 1: Fig. S3). However, we had only 35% power to detect an association with a hazard ratio [HR] of 1.5.

**Table 1:** Single-variant association results for variants reaching exome-wide significance (*p*<4.3×10^−7^), or with evidence of replication in type 1 diabetes (*p*<0.05) or in the general population (*p*<0.05/27/8=2.3×10^−4^)

Gene	Variant	MAF	SIFT/PolyPhen	Phenotype	***N***	***P***-value	Beta (se)	***P***_**Repl**_	***P*** lipids
*LIPC*	15:58563549 C>T p.Thr405Met rs113298164	0.017	Deleterious, probably damaging	apoA1	918	7.8×10^−8^	0.98 (0.18)	0.89	apoA (UKBB): *p*=9.3×10^−46^
*GTF3C5*	9:133054787 C>T p.Ala382Val rs202207045	0.007^b^	Tolerated, benign	LDLC	894	1.3×10^−6^	−1.35 (0.28)	0.02	LDLC (Hindy): *p*=0.125
*GTF3C5*	9:133054787 C>T p.Ala382Val rs202207045	0.007^b^	Tolerated, benign	Non-HDLC	919	7.3×10^−7^	−1.38 (0.28)	0.02	Non-HDLC (Hindy) *p*=0.199
*FNDC3A*	13:49201861 A>G p.Thr1017Ala rs45604939	0.064	Deleterious, probably damaging	CHOL^a^	748	8.8×10^−6^	0.46 (0.10)	0.04	CHOL (Hindy) *p*=0.35
*PPIC*	5:123023945 T>C p.Asn190Ser rs451195	0.156	Deletorious, benign	HDLFC L	487	5.0×10^−6^	0.43 (0.09)	0.87	HDLC (GLGC) *p*=2.1×10^−7^
*ZNF274*	19:58206912 A>G p.Gln118Arg rs45580533	0.021	Tolerated, benign	VLDL L	487	9.3 ×10^−6^	−1.02 (0.23)	0.87	LDL (GLGC) *p*=3.0×10^−13^
*ZNF274*	19:58206912 A>G p.Gln118Arg rs45580533	0.021	Tolerated, benign	VLDLTG L	748	5.5 ×10^−6^	−10.5 (0.23)	0.81	TG (GLGC) *p*=0.003

*Variant* chromosome:base pair position with REF>ALT alleles, amino acid change, and rs identifier, *MAF* minor allele frequency, *SIFT/Polyphen* predicted effect, *Beta (se)* effect size beta and standard error, *P*_*Repl*_*P*-value for replication in GWAS data, *P lipids* the lowest *p*-value within the GLGC GWAS, UKBB WES, and lipid WES (Hindy et al.) for the corresponding or closest matching lipid phenotype

^a^rs45604939 association with total cholesterol was obtained for the NMR measured total cholesterol; replication with standard laboratory total cholesterol

^b^rs202207045 MAF in Hindy et al. was markedly lower, 2.5×10^−5^

Furthermore, 25 variants were suggestively associated with lipid, apolipoprotein, and lipoprotein phenotypes (*p*<1×10^−5^; Additional file 1: Table S4). One of the variants was a 21-bp inframe deletion in the *RBM47* gene (p.Ala496-Ala502del, rs564837143, MAF=1.0%, *p*=2.5×10^−6^) found in the WGS data only, and associated with lower serum apoC-III concentrations, with median apoC-III of 3.74 (IQR=3.38–4.69) mg/dl in the six p.Ala496-Ala502del carriers vs. 7.79 (IQR=5.62–10.51) mg/dl in the non-carriers (Fig. 2C). The variant was nominally associated with TG and VLDL phenotypes (Fig. 2D; Additional file 1: Table S5). In the subsequent analysis of the full WGS data with nine p.Ala496-Ala502del carriers (with or without apoC-III available), three experienced a CAD event during the full study period, not significantly different from the non-carriers (Additional file 1: Fig. S3).

While not reaching our threshold for suggestive significance, we also observed associations for many well-known coding variants associated with lipid traits, e.g., the protective *PCSK9* p.Arg46Leu loss-of-function variant [70] associated with lower cholesterol concentrations (*p*=2×10^−4^; Additional file 1: Table S6).

### Replication of single-variant associations

The FinnDiane GWAS dataset contained 25 of the 26 lead variants with good imputation quality (*r*^*2*^>0.8). Two of these were replicated with nominal significance: p.Thr1017Ala (rs45604939) in *FNDC3A* was associated with higher total cholesterol (MAF=0.063, *p*=0.04); and p.Ala382Val (rs202207045) in *GTF3C5* with lower LDLC and non-HDLC (MAF 0.008, *p*=0.02 for both; Table 1, Additional file 1: Table S4). Furthermore, replication in the GLGC GWAS data, UK Biobank WES, and lipid WES for available standard lipid measurements indicated that *LIPC* p.Thr405Met was significantly associated with apolipoprotein A (apoA; *p*=9.3×10^−46^) and other lipid phenotypes (*p*<0.05/27/8=2.3×10^−4^), rs451195 (p.Asn190Ser) in *PPIC* with HDLC (*p*=2.1×10^−7^), and rs45580533 (p.Gln118Arg) in *ZNF247* with total cholesterol, LDLC, and non-HDLC (*p*<3.0×10^−13^). A total of 15 variants reached a nominal *p*<0.05 for at least one of the studied phenotypes (Additional file 1: Table S7).

### WES and WGS gene-based analysis

We performed SKAT-O gene aggregate tests to identify genes enriched for low-frequency (MAF≤5%) PAVs and PTVs. In WES, PAVs in *AKAP3* were significantly associated (*p*<2.9×10^−6^, adjusted for 17,022 genes) with the triglyceride content of the extremely large VLDL particles (*p*=1.4×10^−7^; Table 2). Furthermore, PTVs in *PTGER3* were significantly associated (*p*<1.0×10^−5^, adjusted for 4810 genes) with free cholesterol in medium-sized HDL particles (*p*=9.8×10^−6^). Two additional genes reached a suggestive *p*-value <1×10^−5^ for PAVs (Table 2). In WGS, SKAT-O analysis revealed that PAVs in *RBM47* were associated with serum apoC-III concentrations (*p*=2.2×10^−6^). Of note, the association was driven by the 21 bp inframe deletion of the *RBM47* gene identified in the WGS single-variant analysis (SKAT *p*=0.28 when p.Ala496-Ala502del excluded). Furthermore, in WGS, PTVs in *SBDS* were also associated with serum apoC-III concentrations (stop gain, and a splice donor variant; *p*=5.0×10^−6^). Finally, a splice donor PTV in the *DEFT1P/DEFT1P2* genes was associated with phospholipids in extra-large VLDL particles (*p*=1.3×10^−6^). Four additional genes had PAVs suggestively associated with lipid phenotypes (*p*<1×10^−5^; Table 2).

**Table 2:** WES and WGS SKAT-O results and the internal replication in the other data set

				Discovery			Replication		
Gene	Gene position	Type	Pheno	***N***	Nvar	***P***	***N***	Nvar	***P***
**Discovery study: WGS**									
*DEFT1P/ DEFT1P2*	8:7006280-7008824	PTV	VLDLPL XL	187	1	***1.3×10***^−***6***^			
*SBDS*	7:66987676-66995696	PTV	apoC-III	294	2	***5.0×10***^−***6***^			
*RBM47*	4:40423254-40629866	PAV	apoC-III	294	3	***2.0×10***^−***6***^	323	1	0.398
*TRMT5*	14:60971448-60981690	PAV	CHOL	329	8	3.5×10^−6^	419	4	0.322
			VLDLPL XS	329	8	5.9×10^−6^	419	4	***0.015***
			IDLFC	329	8	6.8×10^−6^	419	4	***0.019***
			LDLC L	329	8	9.3×10^−6^	419	4	0.436
*CCAR1*	10:68721143-68792377	PAV	VLDL XL	329	2	7.4×10^−6^	419	3	0.555
*CYP3A43*	7:99828012-99866106	PAV	LDLCE M	329	6	8.7×10^−6^	419	3	0.067
			LDLCE L	329	6	8.7×10^−6^	419	3	***0.038***
			LDL M	329	6	9.2×10^−6^	419	3	0.074
*DIPK1A*	1:92832728-92961522	PAV	TG	451	4	9.9×10^−6^	469	3	0.848
**Discovery study: WES**									
*PTGER3*	1:70852352-71047808	PTV	HDLFC M	419	1	***9.8×10***^−***6***^	329	1	0.119
*AKAP3*	12:4615507-4649047	PAV	VLDLTG XXL	304	5	***6.0×10***^−***7***^	245	5	0.766
*TTYH1*	19:54415430-54436719	PAV	HDLCE L	419	3	5.7×10^−6^			
*ATP4A*	19:35550192-35563658	PAV	HDLTG XL	419	3	8.1×10^−6^	329	3	0.810

All genes reaching a suggestive *p*-value <1×10^−5^ are shown. *P*-values <2.9×10^−6^ for PAVs, and *p*-values <10^−5^ for PTV burden were defined as statistically significant, highlighted in italics; in replication, *p*-values <0.05 are highlighted in italics

*Discovery* exome-wide analysis of either WGS or WES data, *Replication* for WGS, replication in WES data; for WES, replication in WGS data, *Gene position* chromosome number:start-end; if multiple gene isoforms exist, only one set of coordinates are given, *Type* burden of PAVs or PTVs, *Nvar* number of variants of the given variant type in the gene in discovery/ replication data, *apoC-III* serum apolipoprotein C-III, *CHOL* total cholesterol, *TG* triglycerides, *HDLCE L* cholesterol ester in large HDL, *HDLFC M* free cholesterol in medium HDL, *HDLTG XL* TG in extra-large HDL, *IDLFC* free cholesterol in IDL particles, *LDL M* total lipids in medium LDL, *LDLC L* cholesterol in large LDL, *LDLCE M/L* cholesterol esters in medium/large LDL, *VLDL XL* total lipids in extra-large VLDL, *VLDLPL XS/XL* phospholipid in extra small/extra-large VLDL, *VLDLTG XXL* TG in extremely large VLDL

Given the lack of available WES studies of individuals with type 1 diabetes and with rich lipidomic data, we sought for replication of the suggestive SKAT-O results by performing an internal replication between the two data sets. The PAVs of the *TRMT5* gene were suggestively associated in WGS with free cholesterol in IDL particles (*p*=6.8×10^−6^) and with phospholipids in extra small VLDL particles (*p*=5.9×10^−6^), and these associations were replicated in WES (*p*=0.019 and *p*=0.015, respectively; Table 2). In addition, the suggestive association between PAVs in *CYP3A43*, and cholesterol esters in large LDL particles in WGS (*p*=8.7×10^−6^), was replicated in WES (*p*=0.038). *CYP3A43* encodes a member of the cytochrome P450 proteins, which metabolize endogenous compounds and xenobiotics; in special, the cholesterol-lowering statins are extensively metabolized by two other CYP3A family members *CYP3A4* and *CYP3A5* [71]. Analysis stratified by the use of statins suggested that PAVs in *CYP3A43* were associated with lower cholesterol esters in large LDL particles among those using statin medication in particular (Additional file 1: Fig. 4A).

### Gene-level meta-analysis

**Fig. 3:**
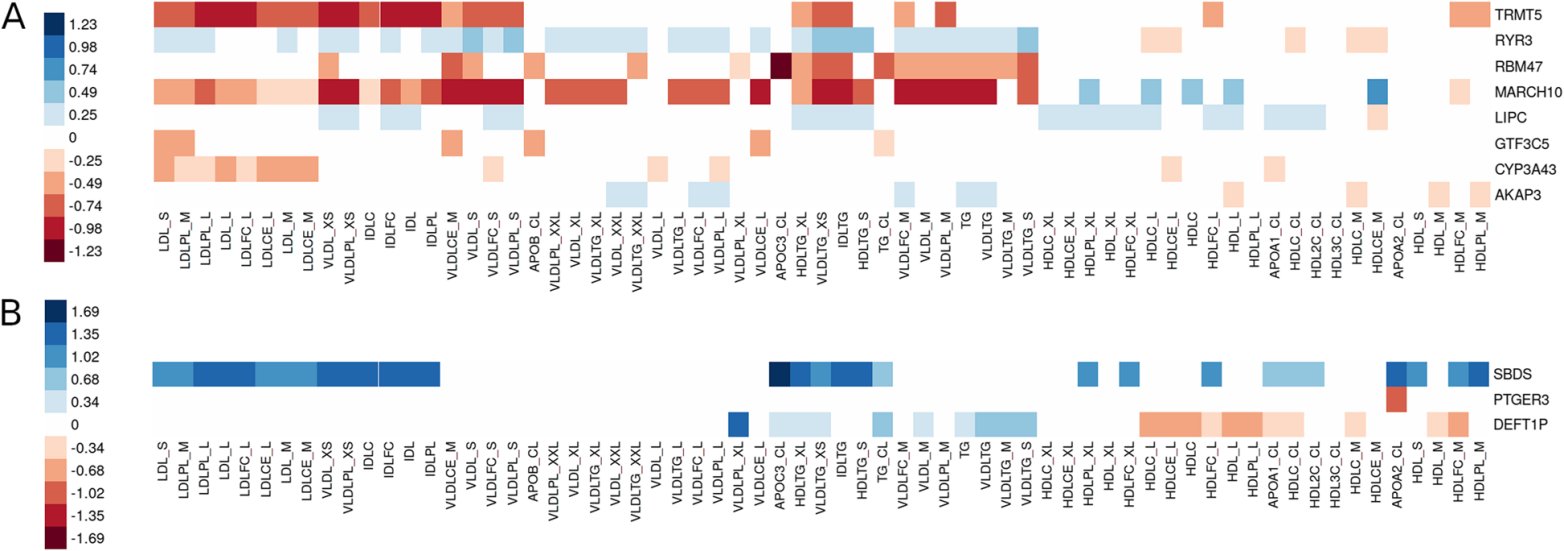
Associations across all studied lipid phenotypes for the lead genes. Only nominally significant associations (WES-WGS SKAT meta-analysis *p*<0.05) are shown. The color indicates the effect size estimate of the gene–phenotype association calculated with a burden test meta-analysis for normalized distributions assuming same effect direction for all variants. **A** WES-WGS PAV meta-analysis across phenotypes. **B** WES-WGS PTV meta-analysis across phenotypes. The phenotypes are ordered according to their similarity in clustering of the phenotype data

Finally, to increase the statistical power, we performed gene aggregate analysis in the combined WES and WGS data by applying SKAT meta-analysis for PAVs and PTVs with MAF ≤5%. The burden of PAVs was significantly associated (*p*<2.9×10^−6^) with lipid phenotypes in four genes, *LIPC*, *RBM47*, *TRMT5*, and *GTF3C5* (Table 3; Manhattan and QQ-plots in Additional file 1: Fig. S5). PAVs in the *LIPC* gene—including rs113298164 from the single-variant meta-analysis—were associated with serum apoA1 concentrations (*p*=1.48×10^−7^). The PAVs in *RBM47* were associated with serum apoC-III concentrations also in the WES-WGS SKAT meta-analysis (*p*=1.33×10^−6^), and PAVs in *TRMT5* were associated with phospholipids in extra small VLDL particles (*p*=7.87×10^−7^). The *TRMT5* PAVs were nominally associated also with multiple IDL phenotypes (Fig. 3). Finally, PAVs found in the *GTF3C5* gene were associated with total cholesterol, LDLC, and non-HDLC.

**Table 3:** Significant WES-WGS SKAT meta-analysis results for genes enriched for PAVs (*p*<2.9×10^−6^) or PTVs (*p*<1×10^−5^) using SKAT or VT algorithms

				SKAT		VT			
GENE	Type	Pheno	***N***	Nvar	***P***_**Liu**_	Nvar	MAF cutoff	Effect	***P***
*DEFT1P*	PTV	VLDLPL XL	445	1	**1.23×10**^−**6**^	1	0.020	1.157	**1.23×10**^−**6**^
*SBDS*	PTV	apoC-III	617	2	**5.37×10**^−**6**^	2	0.005	1.692	1.86×10^−5^
*LIPC*	PAV	apoA1	918	6	**1.48×10**^−**7**^	4	0.017	1.004	**2.12×10**^−**8**^
*GTF3C5*	PAV	Non-HDLC	919	8	**6.86×10**^−**7**^	8	0.007	−0.536	0.014
		LDL Friedewald	920	8	**9.27×10**^−**7**^	8	0.007	−0.705	1.35×10^−3^
		Total cholesterol	920	8	**1.30×10**^−**6**^	8	0.007	−0.522	0.016
*TRMT5*	PAV	VLDLPL XS	738	8	**7.87×10**^−**7**^	8	0.007	−0.985	**3.20×10**^−**7**^
*RBM47*	PAV	apoC-III	617	3	**1.33×10**^−**6**^	3	0.005	−1.228	7.20×10^−4^
*RYR3*	PAV	VLDLTG XS	748	25	1.30×10^−4^	24	0.009	0.530	**2.08×10**^−**6**^
*MARCH10*	PAV	VLDLPL XS	748	5	1.14×10^−5^	4	0.003	−1.498	**2.24×10**^−**6**^

*Type* burden of PAVs or PTVs, *Nvar* number of variants of the given variant type, *P*_*Liu*_*P*-value for SKAT gene burden, calculated with Liu method, *Effect* pooled effect size estimate *β* from the VT test, *VLDLPL XS/XL* phospholipid in extra small/extra-large VLDL, *VLDLTG XS* triglycerides in extra small VLDL particles

To capture genes with rare variants associated with lipid traits, we additionally performed variant threshold (VT) gene burden test. For most of the SKAT lead genes, the VT selected the same number of variants. In addition, rare variants in two genes, *RYR3* and *MARCH10*, were associated with phospholipid and triglyceride content in extra small VLDL particles (Table 3).

### Replication of gene-level analysis results

Replication of the gene aggregate results was sought from the lipid WES by Hindy et al .[13] and UK Biobank WES [32] for available standard lipids. Variants in *LIPC* were associated with apoA (*p*=4.9×10^−110^); variants in *RBM47* with apoB (*p*=7.8×10^−4^) and other lipid traits (Table 4). Furthermore, variants in *CYP3A43*, *GTF3C5*, *AKAP3*, and *RYR3* were nominally associated with lipid traits (*p*<0.05).

**Table 4:** Replication of the gene-level associations in the lipid WES by Hindy et al. [13], and in the UK Biobank WES [32] (associations with *p*<0.05)

		Total CHOL		HDL		LDL		TG		TG-to-HDL		apoA		apoB	
GENE	Test	*P*	beta	*P*	beta	*P*	beta	*P*	beta	*P*	beta	*P*	beta	*P*	beta
*LIPC*	Burden	**0.0012**	**2.86**	**1.6×10**^−**25**^	**2.62**	0.046	−1.59	**2.5×10**^−**7**^	**0.05**						
*LIPC*	SKAT			**3.4×10**^−**20**^	**2.62**	**0.0015**	−**1.59**	0.014	0.05						
*LIPC*	UK M1	**5.5×10**^−**4**^	**0.13**	**9.0×10**^−**32**^	**0.41**			**5.3×10**^−**4**^	**0.13**			**1.4×10**^−**35**^	**0.45**		
*LIPC*	UK M3	**3.4×10**^−**15**^	**0.12**	**7.7×10**^−**95**^	**0.29**			**1.6×10**^−**18**^	**0.13**			**4.9×10**^−**110**^	**0.33**	0.038	0.03
*RBM47*	Burden							**0.0013**	−**0.04**	**0.0028**	−**0.05**				
*RBM47*	SKAT							**0.0058**	−**0.04**	**0.0071**	−**0.05**				
*RBM47*	UK M1	0.045	0.24			**0.0027**	**0.37**							**7.8×10**^−**4**^	**0.41**
*CYP3A43*	Burden							0.019	0.02	0.019	0.03				
*CYP3A43*	SKAT							0.0056	0.02	0.013	0.03				
*GTF3C5*	UK M1	0.027	−0.12			0.029	−0.12							0.042	−0.11
*AKAP3*	UK M1											0.032	−0.10		
*RYR3*	UK M3			0.036	−0.02										

*Burden and SKAT* burden/SKAT test in lipid WES by Hindy et al. [13], for PAVs predicted to be deleterious; *UK M1/M3* UK Biobank gene aggregate test for putative loss-of-function variants/deleterious PAVs of 1% [32]. Associations significant after correction for 12 genes are highlighted with bold type. No associations were significant for non-HDLC

We further sought replication for the individual variants contributing to the gene-level meta-analysis results. Among the 63 PAVs found in these lead genes, 34 were found with good imputation quality in the FinnDiane GWAS data. In addition to the abovementioned *GTF3C5* rs202207045 variant association in the GWAS replication data (*p*=0.02 for LDLC and non-HDLC), a *LIPC* p.Phe368Leu (rs3829462) variant was associated with higher apoA1 (MAF=0.046, *p*=0.02), along with a rare (MAF=0.0002, MAC=1.5) low imputation quality (0.37) *LIPC* p.Ser301Phe variant (*p*=0.04; Table 5, Additional file 1: Table S8).

**Table 5:** Association with cardiometabolic endpoints for lead gene variants significant in WES+WGS meta-analysis or GWAS replication

						Stroke + TIA		Stroke		CVD	Statin medication		
GENE	Variant	Consequence	Pheno	***P***_**WES+WGS**_	***P***_**GWAS**_	OR	***P***	OR	***P***	OR	***P***	OR	***P***
*DEFT1P*	rs797006828	splice donor	VLDLPL XL	**1.2×10**^−**6**^									
*SBDS*	rs113993993	splice donor	apoC-III	**5.9×10**^−**6**^	0.19	1.04	0.62	1.04	0.60	0.99	0.81	1.03	0.51
*SBDS*	rs113993991	stop gain	apoC-III	0.55									
*LIPC*	rs121912502	p.Ser301Phe	apoA1	0.35	0.04*	**2.01**	**0.0024**	1.58	0.07	**1.54**	**0.035**	0.97	0.84
*LIPC*	rs3829462	p.Phe368Leu	apoA1	0.99	**0.02**	1.04	0.18	1.03	0.33	1.02	0.40	1	0.91
*LIPC*	rs113298164	p.Thr417Met	apoA1	**7.8×10**^−**8**^	0.89	0.93	0.15	0.98	0.67	0.97	0.43	1.02	0.52
*GTF3C5*	rs189383196	start lost, p.Met126Thr	Non-HDLC	0.38	0.17	0.99	0.81	0.96	0.48	1.02	0.75	**1.12**	**0.0058**
*GTF3C5*	rs202207045	p.Ala382Val	Non-HDLC	**7.3×10**^−**7**^	**0.02**	0.93	0.36	0.96	0.59	0.92	0.21	0.98	0.77
			LDL Fried	**1.3×10**^−**6**^	**0.02**								
			CHOL CL	**1.2×10**^−**6**^	0.11								
*TRMT5*	rs45604437	p.Ala456Val	VLDLPL XS	**5.8×10**^−**4**^	0.53	0.9	0.28	**0.73**	**0.0035**	0.88	0.11	1.08	0.25
*TRMT5*	rs115400838	p.Ser185Cys	VLDLPL XS	**1.6×10**^−**4**^	0.75	**1.17**	**0.0010**	**1.21**	**6×10**^−**4**^	**1.12**	**0.012**	1.06	0.12
*RBM47*	rs564837143	p.Ala496-Ala502del	apoC-III	**2.5×10**^−**6**^	0.08	1.03	0.67	0.97	0.66	1.02	0.70	1.05	0.33
*MARCHF10*	rs147046907	p.Thr560Ile	VLDLPL XS	**1.07×10**^−**6**^	0.76	1.08	0.30	1.09	0.32	0.99	0.89	0.99	0.87
*MARCHF10*	rs916315847	p.Gly143Arg	VLDLPL XS	**0.03**									
*RYR3*	rs2229119	p.Asn898Ser	VLDLTG XS	**7.42×10**^−**3**^	0.22	0.99	0.86	1.04	0.55	1.03	0.50	1.08	**0.0300**
*RYR3*	rs200294137	p.Ile2417Val	VLDLTG XS	**0.04**		0.87	0.41	0.92	0.66	1.05	0.71	1.06	0.56
*RYR3*	rs61996335	p.Pro3085Arg	VLDLTG XS	**0.03**	0.22	0.92	0.27	0.86	0.05	1.02	0.81	1.04	0.38
*RYR3*	rs146201205	p.Met3641Val	VLDLTG XS	**1.39×10**^−**3**^	0.66	0.98	0.70	0.97	0.62	1.01	0.83	0.94	0.08
*RYR3*	rs202181075	p.Asn3849His	VLDLTG XS	**0.01**	0.68	0.97	0.55	0.96	0.36	1.04	0.25	1.08	**0.0082**

The table includes PTVs and variants with *p*<0.05 either in the WES+WGS single-variant meta-analysis or in the FinnDiane GWAS replication for the genes significant in the WES+WGS gene-level meta-analysis. Clinical endpoints were queried in the FinnGen GWAS data: *Stroke + TIA* wide stroke definition including transient ischemic attack (TIA), *Stroke* “stroke, including SAH (no controls excluded)”, *CVD* “Hard cardiovascular diseases” including coronary revascularization event, myocardial infarctions, and strokes excluding subarachnoid hemorrhages

### Association with cardiovascular outcomes

Since dyslipidemia is a major risk factor for diabetic complications, as well as a cardiovascular risk factor in the general population, we investigated whether the lead genes were associated with cardiovascular and kidney outcomes. In the discovery study SKAT meta-analysis of the WES and WGS data for DKD and CVD, PAVs in *CYP3A43* were associated with DKD (*p*=0.004, rank 43/17,578 genes, i.e., top 0.3%: Additional file 1: Table S9). In the UK Biobank WES [9], putative loss-of-function variants (MAF≤1%) in *GTF3C5* were associated with CAD (OR 1.89, 95% CI 1.26–2.84, *p*=0.0022; significant after correction for 12 lead genes, but not for three investigated phenotypes; Additional file 1: Table S10).

In the FinnGen general population GWAS data, among the significant or replicated variants within the lead genes, the *LIPC* p.Ser301Phe variant, as well as the *TRMT5* p.Ala456Val and p.Ser185Cys variants, was associated with the stroke and CVD phenotypes (*LIPC* p.Ser301Phe *p*=0.0024 for the wide stroke definition; *TRMT5* p.Ser185Cys *p*=0.0010 for the wide stroke definition; Table 5). We then extended the FinnGen study GWAS data queries to all identified PAVs in the gene-level meta-analysis lead genes and all 109 cardiovascular endpoints. The strongest evidence of association was found for a rare (MAF=0.004) deleterious start-loss variant rs189383196 in *GTF3C5*, 80-fold enriched in the Finnish population, and associated with non-ischemic cardiomyopathy (*p*=2.8×10^−5^), hypertension (*p*=6.7×10^−4^), and 18 other circulatory phenotypes (*p*<0.05; Additional file 1: Table S11). Also, another rare (MAF=0.001) deleterious rs369889499 (p.Tyr347Cys) variant in *GTF3C5* was 77-fold enriched in the Finns- and associated with multiple phenotypes, including angina pectoris (*p*=9.20×10^−5^) and ischemic heart disease (*p*=6.10×10^−4^). In *MARCHF10*, rs199705946 suggestively associated with lower phospholipid concentrations in the VLDL particles (*p*=0.07) was exclusively found in the Finnish population with MAF of 0.3%, predicted deleterious by SIFT and PolyPhen-2, and was associated with cardiomyopathy (*p*=3.40×10^−5^, OR=3.7). In the *TRMT5* gene, the variant with the strongest individual association, rs115400838 (p.Ser185Cys), was associated with multiple stroke phenotypes, e.g., “stroke, excluding subarachnoid hemorrhage” (*p*=1.90×10^−4^).

### Association for genes causing monogenic forms of dyslipidemia

Previously, rare variants in multiple genes have been associated with severe monogenic forms of dyslipidemia. We studied the PAV and PTV burden in 19 genes causing monogenic dyslipidemias and overlapping previous lipid GWAS loci, including the *LIPC* gene (*p*<0.05/19 = 0.0026 considered significant after correction for multiple testing; Additional file 1: Table S6) [72]. In the hypercholesteremia-causing *APOB* gene, we identified two frameshift PTVs in exon 26/29 (rs1232943044 (p.Ala3215fs) and rs1407451220 (p.Ser1943fs)), associated with low serum non-HDLC (*p*=4.8×10^−4^), apoB (*p*=5.6×10^−4^), and LDLC (*p*=9.5×10^−4^) concentrations (Additional file 1: Fig. S6), as well as with triglyceride content in small VLDL particles (*p*=0.001; Additional file 1: Fig. S7, Additional file 1: Table S6). These PTVs have not been previously associated with lipid traits. In addition to the abovementioned *LIPC* PAV association with serum apoA1 concentrations, the PAVs in *LIPC* were associated with total HDLC and five other lipid phenotypes (Additional file 1: Fig. S7, Additional file 1: Table S6). In the *CETP* gene, known for genetic disorders of the HDL metabolism, PAVs were associated with serum apoA1 concentrations (*p*=6.9×10^−5^), total HDLC (*p*=4.0×10^−5^), and seven other lipid measurements in HDL particles, driven by two low-frequency missense variants, rs5880 and rs1800777 previously associated with low HDLC [73]. PAVs in the hypercholesterolemia-associated *APOE* gene were associated with apoB (*p*=3.5×10^−4^), total HDLC (*p*=8.0×10^−4^), and total cholesterol and cholesterol esters in LDL particles, with large negative effects observed for the previously reported rare p.Glu57Lys (rs201672011) variant [74]. Finally, the three previously reported PAVs in the *PCSK9* gene, including the protective rs11591147 (p.Arg46Leu) loss-of-function mutation [70] were associated with total cholesterol (*p*=3.0×10^−4^), LDLC (*p*=0.0014), and non-HDLC (*p*=4.8×10^−4^).

## Discussion

Dyslipidemia is a considerable risk factor for CVD. In addition to the standard clinical lipid laboratory measurements, here we have used apolipoproteins as well as NMR lipid and lipoprotein measurements, combined with exome sequencing to identify genetic variants associated with a total of 79 studied phenotypes. We identified associations in genes already implicated in lipid metabolism (e.g., rs113298164 in *LIPC*, two novel PTVs in *APOB*), as well as multiple novel genes for lipid phenotypes, e.g., *RBM47* and *SBDS* for apoC-III concentrations, *GTF3C5* for LDLC, and *TRMT5*, *MARCHF10*, and *RYR3* for phospholipids and triglycerides in VLDL particles.

The lead variant in the single-variant analysis, rs113298164 (*LIPC* p.Thr405Met), was associated with elevated apoA1 concentrations (*p*=7.8×10^−8^). In addition, the burden of PAVs in *LIPC* was associated with apoA1 concentrations even after Bonferroni correction for the number of genes and 12 estimated independent phenotypes (*p*<2.4×10^−7^). *LIPC* encodes the hepatic lipase, which is the enzyme responsible for triglyceride hydrolysis in IDL particles and, thus, the conversion of IDL to LDL particles. p.Thr405Met is predicted deleterious or probably damaging by SIFT and PolyPhen-2, and previous functional studies show that p.Thr405Met reduces hepatic lipase activity [75, 76]. With 1.7% MAF, it is over 4-fold enriched in the Finnish population. Previously, p.Thr405Met has been identified to cause hepatic lipase deficiency in a compound heterozygous state with another rare p.Ser301Phe mutation in *LIPC*, causing elevated total cholesterol, triglyceride, and triglyceride-enriched VLDL and LDL particles, followed by premature atherosclerosis [76]; in our GWAS data, also the rs121912502 (p.Ser301Phe) variant was nominally associated (*p*=0.04) with apoA1 despite low imputation quality (0.37) and low MAF (0.0002). ApoA1 is a key structural component of HDL particles—generally associated with a lower risk of CVD. While association with higher apoA1 and HDLC may seem contradictory to the association with high total cholesterol and hypertriglyceridemia, severe hepatic lipase deficiency is characterized by an increase in apoA1, HDLC, and HDL triglyceride content [77], all seen in our data as well.

Common variants in the *LIPC* gene are strongly associated with serum HDLC and apoA1 concentrations [22]. In a recent Mendelian randomization analysis, variants associated with elevated apoA1 concentrations were associated with lower risk of CAD in the univariate analysis; however, this effect disappeared when accounted for variants affecting apoB concentrations [22].

Importantly, we identified two PTVs in *APOB* associated with drastically low serum apoB concentrations (Additional file 1: Fig. S6); to our knowledge, these variants have not been previously associated with lipid traits, and they are not included in the GLGC GWAS [32], nor in the lipid WES by Hindy et al. [13] or UK biobank WES [9]. However, with only three individuals, we do not see any association with CVD endpoints.

In gene aggregate tests, we showed that *RBM47* was associated with lower apoC-III concentrations. This association was driven by rs564837143, a 21 bp inframe deletion (p.Ala496-Ala502del) found in the WGS data, located in the 6th exon. The variant was also associated with triglyceride concentrations, especially in the VLDL particles. We obtained external validation for the association, as the burden of rare deleterious variants in *RBM47* was associated with lower triglyceride levels (*p*=0.0013) and triglycerides-to-HDLC ratio (*p*=0.0028) in lipid WES of >170,000 individuals [13]. In UK Biobank WES, putative loss-of-function variants in *RBM47* were associated with higher apoB (*p*=7.8×10^−4^) and LDLC concentrations (*p*=0.0027). Furthermore, another rare missense variant was recently shown to have a large impact on blood pressure in a large meta-analysis [78]. *RBM47* encodes an RNA-binding protein essential for post-transcriptional modification of the apoB mRNA in particular. This modification creates a premature stop codon in the transcript, resulting in the production of the shorter intestinal isoform apoB-48 instead of the longer isoform apoB-100 produced by the liver [79]. Of note, we have previously shown that apoB-48 is elevated in individuals with type 1 diabetes both at fasting and postprandially [80]. In this study, we do not have apoB isoforms measured for these participants, but we saw a modest association also between *RBM47* variants and lower serum apoB concentrations. Whereas one copy of apoB is firmly embedded within the surface of each TRL (i.e., chylomicrons, VLDL, and IDL) and LDL particle, apoC-III is dynamically redistributed between these and HDL particles in the circulation [81]. ApoC-III is an important regulator of triglyceride metabolism that impairs the clearance of the atherosclerotic, apoB-containing TRLs and their remnants through multiple pathways. One key action of apoC-III is the inhibition of lipoprotein lipase, and to some extent, also hepatic lipase encoded by the *LIPC* gene [21]. There is increasing evidence—also from genetic studies of a rare *APOC3* loss-of-function variant [20, 23]—that apoC-III is an independent cardiovascular risk factor, and clinical trials on apoC-III lowering therapies have yielded positive results in those with high triglycerides. ApoC-III is an important CVD risk factor also in individuals with type 1 diabetes [20] and we recently showed that apoC-III concentrations are elevated in individuals with DKD and predict future DKD progression [82]. However, with a low number of the *RBM47* p.Ala496-Ala502 carriers, we did not have statistical power to observe any association with CVD in our data (Additional file 1: Fig. S3).

PAVs in *GTF3C5* were associated with total cholesterol, LDLC, and non-HDLC. Among the eight PAVs, six were predicted deleterious by SIFT and/or PolyPhen-2. One of them, chr9:133042147_C/T (p.His72Tyr), is a novel variant, with one heterozygous carrier found in our data (verified as good quality from the aligned BAM-file). Another variant, rs189383196, is either a high impact start-loss variant or a missense variant (p.Met126Thr), depending on the transcript, with over 80-fold enrichment in Finns. The association for the strongest individual variant, rs202207045 (p.Ala382Val), was replicated in the GWAS data (*p*=0.02 for LDLC and non-HDLC). The PAVs in this gene were associated with multiple circulatory phenotypes, e.g., non-ischemic cardiomyopathy (*p*=2.8×10^−5^) in the independent FinnGen general population GWAS data. Of note, this variant was not detected in the UK Biobank WES and had an MAF of 0.002% in the lipid WES by Hindy et al., and 0.07% in the GLGC GWAS. Interestingly, the strongest association within the *GTF3C5* region in the FinnGen GWAS data was at rs671412, 28 kbp downstream, with the use of statin medication (*p*=3.4×10^−7^). *GTF3C5* encodes a DNA-binding general transcription factor IIIC subunit 5, expressed in all tissues, and little is known about the function of this gene.

PAVs in *TRMT5* were associated with phospholipids in extra small VLDL particles, both in WES and WGS separately, as well as in WES-WGS SKAT-O meta-analysis. Among the eight identified variants, five were predicted deleterious. As supporting evidence, the deleterious missense variant with the strongest association with lower phospholipids in VLDL particles was associated with a higher risk of stroke in the FinnGen data (*p*=1.90×10^−4^). *TRMT5* encodes a tRNA methyltransferase 5 involved in mitochondrial tRNA methylation and has not previously been associated with lipid traits.

Other novel findings worth mentioning are PTVs in *SBDS*, as well as PAVs in *CYP3A43*, *PTGER3*, and *AKAP3*. Loss-of-function variants in S*BDS* cause autosomal recessive Shwachman-Diamond Syndrome 1, characterized by exocrine pancreatic dysfunction among other symptoms [83]. Our observed association between heterozygous *SBDS* PTVs and apoC-III may be affected by a similar pathway. PAVs in *CYP3A43* were associated with LDL cholesterol esters in WGS and replicated in WES; *CYP3A43* was the only gene with evidence of association with clinical outcome in our WES-WGS data (SKAT *p*=0.004 for DKD, rank 43/17,578 genes). While little is known about the gene, it encodes one of the cytochrome P450 proteins, which are involved in the synthesis of cholesterol, steroids, and other lipids and, importantly, metabolize most of the drugs and can cause toxic drug-drug interactions, e.g., with the statins [84].

It is of note that 460 of the study participants had DKD at the time of their lipid measurement; 239 of these had end-stage renal disease. This can affect the serum lipid concentrations, as DKD [34], and chronic kidney disease (CKD) in general, is associated with lipid concentrations. In particular, CKD is associated with low HDLC and elevated triglycerides due to delayed catabolism of TRLs [85]. In patients with nephrotic syndrome, serum VLDL cholesterol, IDL cholesterol, and triglyceride levels are further increased, e.g., due to impaired urinary clearance, acquired hepatic LDL receptor dysfunction [86], and increased biosynthesis [87]. Also the lipoprotein particle composition is altered in CKD, including elevated apoC-III levels [88], also seen among the FinnDiane participants with DKD [82]. This may have contributed positively to our capacity to detect associations for apoC-III and other lipid variables, but may also have confounded some associations.

One limitation of this study is the lack of replication in other type 1 diabetes studies. We have attempted replication of the findings in individuals with type 1 diabetes using our GWAS data and internal replication between the WES and WGS gene aggregate findings, but we note that these data sets have limitations for replication. While some of the observed associations may be specific to individuals with diabetes, e.g., through disturbances in the insulin signalling, we hypothesize that many of the associations observed in this high-risk population may be generalized to the wider population, as many of the single-variant and gene-level findings were nominally replicated in the general population data sets. On the contrary, lack of replication in the general population can indicate either a false positive finding, specificity to (type 1) diabetes, or lack of statistical power for replication, e.g., due to lower variant frequency in non-Finnish populations, and thus, we cannot elucidate whether these associations are specific to diabetes.

It is of note that the significance thresholds were only adjusted for the number of studied variants or genes, not for the number of phenotypes. After additional correction for 12 estimated independent phenotypes obtained from the PCA, only the *LIPC* gene aggregate association with apoA1 concentrations would remain significant (*p*<2.4×10^−7^); if considering only the number of genes with the required cumulative MAC of ≥5, also *TRMT5* and *DEFT1P* would remain significant after correction for the number of genes and 12 independent phenotypes. Finally, the number of individuals in the study remains moderate, with limited statistical power. Post hoc power calculations indicated that we had 65% power to detect the lead association on the *LIPC* gene with exome-wide significance; we had only moderate power to detect associations for low-frequency variants with smaller effect size. Nevertheless, we were able to identify multiple novel genetic associations, especially with the gene aggregate tests that increase the statistical power. Of note, many of the identified variants were markedly enriched in the Finnish population, e.g., the 80-fold enriched *GTF3C5* PAVs, providing one potential explanation why these variants have not been detected in earlier studies. It is of note that many previous, larger studies were either based on chip genotyping [8, 32] or included only the standard clinical lipid measurements such as total cholesterol, LDLC, HDLC, and triglyceride concentrations [13, 32]. While limited evidence of replication was found for the single-variant associations in the FinnDiane GWAS data, many of the identified PAVs or genes were associated with relevant metabolic traits and clinical endpoints in larger external data sets.

## Conclusions

This study represents the first comprehensive analysis of PAVs associated with detailed lipid, apolipoprotein, and lipoprotein phenotypes in individuals with type 1 diabetes. We identified both novel variant associations in known lipid genes, as well as novel genes implicated in lipoprotein metabolism. Previous studies suggest that apoC-III is an important, independent risk factor for CVD. While we identified a seven amino acid deletion in *RBM47* associated with lower apoC-III concentrations, further studies are needed to elucidate the biological mechanism that it exerts on the apolipoprotein concentrations.

## Supplementary Information

**Additional file 1: Supplementary material.** A combined document including all supplementary tables (Tables S1-S11), figures (Figs. S1-S7), and code (Text S1).

## Data Availability

The sequencing data supporting the current study have not been deposited in a public repository because of restrictions due to the study consent. The summary statistics of the 79 lipidomics phenotypes, including the single-variant results, as well as the SKAT and VT results for PAVs and PTVs are available in the figshare [89] and at the type 1 diabetes knowledge portal (https://t1d.hugeamp.org/downloads.html) and common metabolic diseases knowledge portal (https://md.hugeamp.org/downloads.html). The readers may propose collaboration to research the individual-level data with correspondence with the lead investigator. Example code to run WES/WGS single-variant and gene aggregate meta-analysis is given in Additional file 1: Text S1.

## Abbreviations

AER: Albumin excretion rate
apoA1: Apolipoprotein A1
apoB: Apolipoprotein B
apoC-III: Apolipoprotein C-III
CAD: Coronary artery disease
CL: Central research laboratory of Helsinki University Hospital, Finland
CVD: Cardiovascular disease
DKD: Diabetic kidney disease
FinnDiane: The Finnish Diabetic Nephropathy Study
GWAS: Genome-wide association study
HDLC: High-density lipoprotein cholesterol
HWE: Hardy Weinberg equilibrium
IDL: Intermediate-density lipoprotein
IQR: Inter-quartile range
LDL: Low-density lipoprotein
LDLC: Low-density lipoprotein cholesterol
Lp[a]: Lipoprotein(a)
MAC: Minor allele count
MAF: Minor allele frequency
NMR: Nuclear magnetic resonance
PAV: Protein-altering variant
PTV: Protein-truncating variant
TRL: Triglyceride-rich lipoproteins
T2D: Type 2 diabetes
VLDL: Very-low-density lipoprotein
WES: Whole-exome sequencing
WGS: Whole-genome sequencing
