# Supplementary material for: Whole-exome sequencing identifies novel protein-altering variants associated with serum apolipoprotein and lipid concentrations
Source: Genome Med. 2022 Nov 23;14:132. doi: 10.1186/s13073-022-01135-6 (PMC9685920; doi:10.1186/s13073-022-01135-6)
Supplement: Supplementary file 1 — Additional file 1: Supplementary material. A combined document including all supplementary tables (Tables S1-S11), figures (Figs. S1-S7), and code (Text S1). [file 13073_2022_1135_MOESM1_ESM.docx]

**Whole-exome sequencing identifies novel protein-altering variants associated with serum apolipoprotein and lipid concentrations**

Niina Sandholm*, Ronja Hotakainen*, Jani K Haukka, Fanny Jansson Sigfrids, Emma H Dahlström, Anni Antikainen, Erkka Valo, Anna Syreeni Elina Kilpeläinen, Anastasia Kytölä, Aarno Palotie, Valma Harjutsalo, Carol Forsblom, Per-Henrik Groop, on behalf of the FinnDiane Study Group

**Supplementary material**

**Table of contents**

[Table S1: Baseline clinical characteristics of the participants. 2](#_Toc117760522)

[Table S2. Lipid, lipoprotein, and apolipoprotein phenotypes. 3](#_Toc117760523)

[Table S3: Number of PAVs and PTVs in the WES and WGS data. Counts are calculated for the total cholesterol lab measurements. 6](#_Toc117760524)

[Table S4: Single variant WES+WGS meta-analysis results for associations with p<1×10^-5^ 7](#_Toc117760525)

[Table S5: Lipidome-wide association results for the *LIPC* p.Thr405Met (rs113298164) and *RBM47* p.Ala496-Ala502del (rs564837143) variants. 9](#_Toc117760526)

[Table S6. Significant associations (WES/WGS SKAT meta-analysis p<0.05/19 genes=0.0026) for PTVs and PAVs in known lipid genes. 11](#_Toc117760527)

[Table S7: Replication p-values for the single variant meta-analysis lead variants in the GLGC GWAS, UKBB WES, and Lipid WES. 15](#_Toc117760528)

[Table S8: Single variant score test association results for PTVs and PAVs within the lead genes from the WES+WGS SKAT or VT meta-analysis. 16](#_Toc117760529)

[Table S9: WES-WGS SKAT meta-analysis on coronary artery disease (CAD), Strokes, cardiovascular disease (CVD; defined as CAD or stroke), and diabetic kidney disease (DKD) for the lead genes. 19](#_Toc117760530)

[Table S10: Lead gene associations with cardiovascular outcomes in the UK Biobank WES by Backman et al. 2021. 20](#_Toc117760531)

[Table S11: Variant association with “Diseases of the circulatory system” phenotypes in the FinnGen GWAS data. 21](#_Toc117760532)

[Table S12: FinnDiane physicians and nurses participating in the collection of the FinnDiane study subjects 30](#_Toc117760533)

[Fig. S1: Power calculations. 33](#_Toc117760534)

[Fig. S2: Correlation plot of analyzed lipid phenotypes. 34](#_Toc117760535)

[Fig. S3: Survival analysis for coronary artery disease (CAD) and strokes for the *LIPC* p.Thr405Met and *RBM47* p.Ala496-Ala502del variants. 35](#_Toc117760536)

[Fig. S4: PAVs in *CYP3A43* are associated with lower cholesterol esters in large LDL particles. 36](#_Toc117760537)

[Fig. S5: WES+WGS SKAT meta-analysis results, Manhattan and QQ-plots. 37](#_Toc117760538)

[Fig. S6: *APOB* rs1407451223 (N=2) or rs1232943044 (N=1) protein truncating frameshift variants are associated with non-HDL and LDL cholesterol and Apolipoprotein B (apoB) concentrations. 39](#_Toc117760539)

[Fig. S7: WES-WGS SKAT meta-analysis results for genes previously associated with rare lipid disorders. 40](#_Toc117760540)

[References 41](#_Toc117760541)

[Text S1: Example code to run WES/WGS meta-analysis of the coding variants 42](#_Toc117760542)

# Table S1: Baseline clinical characteristics of the participants.

|  |  | **WES** | **WGS** | **Overall** |
| --- | --- | --- | --- | --- |
|  |  | **(N=443)** | **(N=436)** | **(N=879)** |
| Sex | N (%) | 210 (45.5%) | 259 (57.0%) | 469 (51.2%) |
| Age, years | Mean (SD) | 45.4 (10.9) | 45.2 (10.1) | 45.3 (10.5) |
|  | Median [Min, Max] | 46.6 [20.5, 81.8] | 44.4 [17.6, 76.6] | 45.4 [17.6, 81.8] |
| Diabetes duration, years | Mean (SD) | 32.0 (9.17) | 32.2 (8.23) | 32.1 (8.72) |
|  | Median [Min, Max] | 32.3 [9.75, 59.5] | 31.8 [3.88, 58.5] | 32.0 [3.88, 59.5] |
| Age at diabetes onset, years | Mean (SD) | 13.4 (7.21) | 13.0 (8.04) | 13.2 (7.63) |
|  | Median [Min, Max] | 12.3 [1.26, 34.9] | 11.7 [0.487, 39.7] | 11.9 [0.487, 39.7] |
| Calendar year of diabetes onset | Mean (SD) | 1970 (8.61) | 1970 (8.14) | 1970 (8.39) |
|  | Median [Min, Max] | 1970 [1940, 1990] | 1970 [1950, 2000] | 1970 [1940, 2000] |
| Lipid lowering medication | N (%) | 96 (20.8%) | 109 (24.0%) | 205 (22.4%) |
|  | Missing | 1 (0.2%) | 7 (1.5%) | 8 (0.9%) |
| Diabetic nephropathy stage | Normal AER | 233 (50.4%) | 220 (48.5%) | 453 (49.5%) |
|  | Microalbuminuria | 4 (0.9%) | 19 (4.2%) | 23 (2.5%) |
|  | Macroalbuminuria | 122 (26.4%) | 89 (19.6%) | 211 (23.0%) |
|  | Kidney failure | 103 (22.3%) | 121 (26.7%) | 224 (24.5%) |
|  | Unknown/ other | 0 (0%) | 5 (1.1%) | 5 (0.5%) |
| Body mass index, kg/m2 | Mean (SD) | 25.0 (3.45) | 24.8 (3.87) | 24.9 (3.67) |
|  | Median [Min, Max] | 24.8 [15.0, 40.9] | 24.2 [16.7, 39.6] | 24.6 [15.0, 40.9] |
|  | Missing | 8 (1.7%) | 11 (2.4%) | 19 (2.1%) |
| Systolic blood pressure | Mean (SD) | 141 (20.2) | 142 (22.4) | 142 (21.3) |
|  | Median [Min, Max] | 140 [86.5, 255] | 140 [91.0, 224] | 140 [86.5, 255] |
|  | Missing | 11 (2.4%) | 19 (4.2%) | 30 (3.3%) |
| Diastolic blood pressure | Mean (SD) | 80.7 (11.0) | 80.1 (10.8) | 80.4 (10.9) |
|  | Median [Min, Max] | 80.0 [50.0, 120] | 80.0 [47.0, 112] | 80.0 [47.0, 120] |
|  | Missing | 12 (2.6%) | 19 (4.2%) | 31 (3.4%) |

# Table S2. Lipid, lipoprotein, and apolipoprotein phenotypes.

| **Phenotype** | **Abbreviation** | **N WES** | **N WGS** | **N Total** | **Mean (SD)** | **Median [Min, Max]** | **Method** |
| --- | --- | --- | --- | --- | --- | --- | --- |
| Serum apolipoprotein A-I [mg/dl] | APOA1 | 469 | 449 | 918 | 138 (23.5) | 138 [55.0, 258] | Cobas Mira analyzer (Boehringer-Mannheim) until January 2002, Wako Chemicals GmbH (Neuss, Germany) until January 2006,Konelab 60i analyser and commercial kit (Thermo Fischer Scientific, Waltham, MA, USA) from 2006 onward. |
| Serum apolipoprotein A-II [mg/dl] | APOA2 | 455 | 383 | 838 | 32.3 (8.24) | 31.0 [12.0, 78.0] | Cobas Mira analyzer using immunoprecipitation (Boehringer-Mannheim) until August 2001, and thereafter with a polyclonal antibody. |
| Serum apolipoprotein B-100 [mg/dl] | APOB | 469 | 449 | 918 | 89.9 (25.4) | 88.0 [25.0, 252] | Immunoassay (Orion Diagnostica, Espoo, Finland) until January 2006, and since then with Konelab 60i analyser and a commercial kit (Thermo Fischer Scientific, Waltham, MA, USA). |
| Serum Apolipoprotein C-III [mg/dl] | APOC3 | 323 | 294 | 617 | 8.41 (4.50) | 7.38 [2.34, 33.4] | Immunoassay (Kamiya Biomedical Company, Tukwila, WA 98168, USA). |
| Serum total cholesterol [mmol/l] | CHOL CL | 469 | 451 | 920 | 5.40 (1.25) | 5.23 [2.20, 13.9] | Autoanalyzer with an enzymatic technique (Hoffman-La Roche, Basel, Switzerland until November 2001; ABX Diagnostics, Montpellier, France until January 2006; and Konelab 60i analyser and a commercial kit (Thermo Fischer Scientific, Waltham, MA, USA) from this point onward. |
| Serum HDL cholesterol [mmol/l] | HDLC | 469 | 450 | 919 | 1.33 (0.410) | 1.29 [0.280, 3.21] | Enzymatic method, described in Tolonen, Forsblom et al. 2008. |
| Serum HDL_2_ cholesterol [mmol/l] | HDL2C | 463 | 441 | 904 | 0.584 (0.309) | 0.546 [0.0310, 1.94] | Calculated by subtracting of HDL3-cholesterol from the total HDL-cholesterol. |
| Serum HDL_3_ cholesterol [mmol/l] | HDL3C | 463 | 441 | 904 | 0.756 (0.209) | 0.740 [0.151, 1.68] | Enzymatic method, described in Tolonen, Forsblom et al 2008. |
| Serum triglycerides [mmol/l] | TG | 469 | 451 | 920 | 1.40 (0.962) | 1.09 [0.320, 9.07] | Autoanalyzer with an enzymatic technique (Hoffman-La Roche, Basel, Switzerland until November 2001; ABX Diagnostics, Montpellier, France until January 2006; and Konelab 60i analyser and a commercial kit (Thermo Fischer Scientific, Waltham, MA, USA) from this point onward. |
| LDL cholesterol [mmol/l] calculated with Friedewald equation,  TotalChol - (Triglyceride / 5) - HDL | LDL friedewald | 469 | 451 | 920 | 3.78 (1.19) | 3.61 [0.768, 10.8] | Friedewald equation described in Friedewald, Levy et al. 1972. |
| Non-HDL cholesterol: TOTCHOL- HDLCHOL [mmol/l] | NonHDLC | 469 | 450 | 919 | 4.07 (1.30) | 3.88 [0.988, 11.8] | Calculated by subtracting of serum HDL-cholesterol from the serum total cholesterol. |
| Remnant cholesterol:  TotalChol - HDL - LDL [mmol/l] | Remnant chol | 469 | 450 | 919 | 0.272 (0.174) | 0.218 [0.0640, 1.68] | Calculated by subtracting of HDL-and LDL-cholesterol from the serum total cholesterol described in Varbo, benn et al. 2014. |
| Total cholesterol | CHOL | 419 | 329 | 748 | 5.27 (1.27) | 5.10 [1.53, 13.8] | NMR |
| Total triglycerides | TG | 419 | 329 | 748 | 1.46 (0.788) | 1.25 [0.407, 8.08] | NMR |
| Total lipids in extremely large VLDL | VLDL XXL | 419 | 329 | 748 | 0.0922 (0.144) | 0.0483 [0, 1.68] | NMR |
| Phospholipid in extremely large VLDL | VLDLPL XXL | 419 | 329 | 748 | 0.356 (1.08) | 0.00487 [0, 12.8] | NMR |
| Triglycerides in extremely large VLDL | VLDLTG XXL | 304 | 245 | 549 | 0.0284 (0.0401) | 0.0160 [0, 0.404] | NMR |
| Total lipids in extra large VLDL | VLDL XL | 419 | 329 | 748 | 1.48 (4.02) | 0.119 [0, 43.5] | NMR |
| Phospholipid in extra large VLDL | VLDLPL XL | 258 | 187 | 445 | 0.0217 (0.0167) | 0.0188 [0, 0.124] | NMR |
| Triglycerides in extra large VLDL | VLDLTG XL | 419 | 319 | 738 | 0.393 (0.966) | 0.0742 [0, 11.1] | NMR |
| Total lipids in large VLDL | VLDL L | 419 | 319 | 738 | 0.513 (0.709) | 0.323 [0, 8.35] | NMR |
| Cholesterol in large VLDL | VLDLC L | 419 | 319 | 738 | 0.130 (0.160) | 0.0884 [0, 1.77] | NMR |
| Cholesterol ester in large VLDL | VLDLCE L | 419 | 319 | 738 | 0.0541 (0.0495) | 0.0430 [0, 0.546] | NMR |
| Free cholesterol in large VLDL | VLDLFC L | 419 | 319 | 738 | 0.0767 (0.123) | 0.0408 [0, 1.40] | NMR |
| Phospholipid in large VLDL | VLDLPL L | 419 | 319 | 738 | 0.105 (0.183) | 0.0500 [0, 2.21] | NMR |
| Triglycerides in large VLDL | VLDLTG L | 419 | 319 | 738 | 0.285 (0.397) | 0.176 [0, 4.80] | NMR |
| Total lipids in medium VLDL | VLDL M | 419 | 319 | 738 | 0.532 (0.292) | 0.476 [0.0480, 2.95] | NMR |
| Cholesterol in medium VLDL | VLDLC M | 419 | 319 | 738 | 0.203 (0.0859) | 0.187 [0.00994, 0.794] | NMR |
| Cholesterol ester in medium VLDL | VLDLCE M | 419 | 319 | 738 | 0.107 (0.0367) | 0.102 [0, 0.337] | NMR |
| Free cholesterol in medium VLDL | VLDLFC M | 419 | 319 | 738 | 0.0786 (0.0457) | 0.0691 [0, 0.487] | NMR |
| Phospholipid in medium VLDL | VLDLPL M | 419 | 319 | 738 | 0.103 (0.0563) | 0.0903 [0, 0.577] | NMR |
| Triglycerides in medium VLDL | VLDLTG M | 419 | 319 | 738 | 0.244 (0.161) | 0.212 [0, 1.61] | NMR |
| Total lipids in small VLDL | VLDL | 419 | 319 | 738 | 0.779 (0.272) | 0.724 [0.134, 3.06] | NMR |
| Cholesterol in small VLDL | VLDLC | 419 | 319 | 738 | 0.362 (0.127) | 0.341 [0, 1.28] | NMR |
| Free cholesterol in small VLDL | VLDLFC | 419 | 319 | 738 | 0.122 (0.0430) | 0.115 [0, 0.482] | NMR |
| Phospholipid in small VLDL | VLDLPL | 419 | 319 | 738 | 0.172 (0.0643) | 0.161 [0, 0.700] | NMR |
| Triglycerides in small VLDL | VLDLTG | 419 | 319 | 738 | 0.266 (0.126) | 0.236 [0.0363, 1.36] | NMR |
| Total lipids in extra small VLDL | VLDL XS | 419 | 319 | 738 | 0.561 (0.188) | 0.527 [0, 2.21] | NMR |
| Phospholipid in extra small VLDL | VLDLPL XS | 419 | 319 | 738 | 0.133 (0.0496) | 0.127 [0, 0.571] | NMR |
| Triglycerides in extra small VLDL | VLDLTG XS | 419 | 319 | 738 | 0.112 (0.0481) | 0.100 [0.0233, 0.457] | NMR |
| Triglycerides in VLDL particles | VLDLTG | 419 | 319 | 738 | 0.937 (0.570) | 0.804 [0.0933, 5.68] | NMR |
| Total lipid in IDL particles | IDL | 419 | 319 | 738 | 1.20 (0.343) | 1.17 [0, 3.63] | NMR |
| Cholesterol in IDL particles | IDLC | 419 | 319 | 738 | 0.812 (0.241) | 0.792 [0, 2.23] | NMR |
| Free cholesterol in IDL particles | IDLFC | 419 | 319 | 738 | 0.212 (0.0661) | 0.207 [0, 0.642] | NMR |
| Phospholipid in IDL particles | IDLPL | 419 | 319 | 738 | 0.277 (0.0800) | 0.269 [0, 0.838] | NMR |
| Triglyceride in IDL particles | IDLTG | 419 | 319 | 738 | 0.0981 (0.0469) | 0.0887 [0, 0.485] | NMR |
| Total lipid in large LDL | LDL L | 419 | 319 | 738 | 1.54 (0.411) | 1.50 [0.405, 4.58] | NMR |
| Cholesterol in large LDL | LDLC L | 419 | 319 | 738 | 1.16 (0.360) | 1.11 [0.238, 3.92] | NMR |
| Cholesterol ester in large LDL | LDLCE L | 419 | 319 | 738 | 0.784 (0.225) | 0.770 [0.138, 2.37] | NMR |
| Free cholesterol in large LDL | LDLFC L | 419 | 319 | 738 | 0.293 (0.0741) | 0.288 [0.0676, 0.753] | NMR |
| Phospholipid in large LDL | LDLPL L | 419 | 319 | 738 | 0.351 (0.0859) | 0.343 [0.108, 0.964] | NMR |
| Total lipid in medium LDL | LDL M | 419 | 319 | 738 | 0.949 (0.260) | 0.926 [0.119, 3.06] | NMR |
| Cholesterol in medium LDL | LDLC M | 419 | 319 | 738 | 0.715 (0.231) | 0.677 [0.0579, 2.31] | NMR |
| Cholesterol ester in medium LDL | LDLCE M | 419 | 319 | 738 | 0.468 (0.141) | 0.457 [0.0205, 1.58] | NMR |
| Phospholipid in medium LDL | LDLPL M | 419 | 319 | 738 | 0.235 (0.0578) | 0.228 [0.0558, 0.683] | NMR |
| Total lipid in small LDL | LDL S | 419 | 319 | 738 | 0.675 (0.189) | 0.651 [0.0879, 2.27] | NMR |
| Cholesterol in small LDL | LDLC S | 419 | 319 | 738 | 0.486 (0.160) | 0.457 [0.0752, 1.58] | NMR |
| Cholesterol in LDL particles | LDLC | 419 | 319 | 738 | 2.27 (0.648) | 2.20 [0.367, 6.67] | NMR |
| Total lipid in extra large HDL | HDL XL | 419 | 319 | 738 | 0.555 (0.294) | 0.538 [0, 2.89] | NMR |
| Cholesterol in extra large HDL | HDLC XL | 419 | 319 | 738 | 0.382 (0.189) | 0.365 [0, 1.35] | NMR |
| Cholesterol ester in extra large HDL | HDLCE XL | 419 | 319 | 738 | 0.294 (0.161) | 0.274 [0, 1.06] | NMR |
| Free cholesterol in extra large HDL | HDLFC XL | 419 | 319 | 738 | 0.0760 (0.0627) | 0.0670 [0, 0.640] | NMR |
| Phospholipid in extra large HDL | HDLPL XL | 419 | 319 | 738 | 0.164 (0.125) | 0.143 [0, 1.19] | NMR |
| Triglyceride in extra large HDL | HDLTG XL | 419 | 319 | 738 | 0.0102 (0.00671) | 0.00918 [0, 0.0671] | NMR |
| Total lipid in large HDL | HDL L | 419 | 319 | 738 | 0.316 (0.207) | 0.285 [0, 1.32] | NMR |
| Cholesterol in large HDL | HDLC L | 419 | 319 | 738 | 0.243 (0.155) | 0.229 [0, 0.970] | NMR |
| Cholesterol ester in large HDL | HDLCE L | 419 | 319 | 738 | 0.214 (0.145) | 0.202 [0, 0.939] | NMR |
| Free cholesterol in large HDL | HDLFC L | 419 | 319 | 738 | 0.0312 (0.0247) | 0.0274 [0, 0.168] | NMR |
| Phospholipid in large HDL | HDLPL L | 419 | 319 | 738 | 0.108 (0.0782) | 0.0946 [0, 0.540] | NMR |
| Total lipid in medium HDL | HDL M | 419 | 319 | 738 | 0.438 (0.144) | 0.438 [0, 0.985] | NMR |
| Cholesterol in medium HDL | HDLC M | 419 | 319 | 738 | 0.249 (0.108) | 0.257 [0, 0.618] | NMR |
| Cholesterol ester in medium HDL | HDLCE M | 419 | 319 | 738 | 0.217 (0.0941) | 0.223 [0, 0.510] | NMR |
| Free cholesterol in medium HDL | HDLFC M | 419 | 319 | 738 | 0.0337 (0.0169) | 0.0330 [0, 0.109] | NMR |
| Phospholipid in medium HDL | HDLPL M | 419 | 319 | 738 | 0.189 (0.0591) | 0.187 [0, 0.445] | NMR |
| Total lipid in small HDL | HDL S | 419 | 319 | 738 | 0.881 (0.123) | 0.880 [0.277, 1.37] | NMR |
| Triglycerides in small HDL | HDLTG S | 419 | 319 | 738 | 0.00952 (0.00643) | 0.00837 [0, 0.0526] | NMR |
| Cholesterol in HDL particles | HDLC | 419 | 319 | 738 | 1.18 (0.307) | 1.17 [0.176, 2.74] | NMR |

N: Number of patients in WES and WGS cohorts. NMR: Nuclear Magnetic Resonance.

# **Table S3**: **Number of PAVs and PTVs in the WES and WGS data.** Counts are calculated for the total cholesterol lab measurements.

|  |  | PAV | | | PTV | | |
| --- | --- | --- | --- | --- | --- | --- | --- |
|  | N | MAF=50% | MAF=5% | MAF=1% | MAF=50% | MAF=5% | MAF=1% |
| WGS | 451 | 101,718 | 80,700 | 65,534 | 9,577 | 7,866 | 6,497 |
| WES | 469 | 42,682 | 34,881 | 27,257 | 2,240 | 2,009 | 1,749 |
| Meta-analysis, all | 920 | 115,923 | 97,159 | 82,428 | 7,554 | 6,940 | 6,197 |
| Meta-analysis, MAC ≥ 5 | 920 | 48,077 | 29,261 | 14,543 | 2,301 | 1686 | 943 |
|  |  |  | proportion of PAV | | proportion of PAV | Proportion of PTV | |
| WGS |  |  | 79 % | 64 % | 9 % | 82 % | 68 % |
| WES |  |  | 82 % | 64 % | 5 % | 90 % | 78 % |
| Meta-analysis, all |  |  | 84 % | 71 % | 7 % | 92% | 82% |
| Meta-analysis, MAC≥5 |  |  |  |  | 5% | 73% | 41% |

PTV= Protein-truncating variant. PAV= Protein-altering variant. MAC= Minor allele count

# Table S4: Single variant WES+WGS meta-analysis results for associations with p<1×10^-5^

| **Gene** | **Variant** | **MAF** | **MAC** | **Phenotype** | **Beta** | **SE** | **P** | **Effect** | **Dir** | **P GWAS** |
| --- | --- | --- | --- | --- | --- | --- | --- | --- | --- | --- |
| *CAPZB* | 1:19448881:G:A  (rs79308175,missense) | 0.013 | 19  19  19  19 | TG  VLDLPL M  VLDLTG XS  VLDLTG S | -1.09  -1.07  -1.06  -1.06 | 0.24  0.24  0.24  0.24 | 7.2×10^-6^  5.9×10^-6^  7.2×10^-6^  4.0×10^-6^ | Benign,  Possibly deleterious | ?-  ?-  ?-  ?- | 0.41  0.33  0.32  0.41 |
| *RBM47* | 4:40432687:  AGCGGCTGCGGCGGCTGCGGCC:A | 0.005 | 6 | APOC3 | -1.94 | 0.41 | 2.5×10^-6^ | unknown | ?- | 0.08 |
| *NOCT* | 4:139045355:G:A  (rs144899070, missense) | 0.005 | 9 | Total Cholesterol | 1.55 | 0.34 | 3.8×10^-6^ | Benign,  Tolerated | ++ | 0.63 |
| *PPIC* | 5:123023945:T:C  (rs451195, missense) | 0.156 | 152 | HDLFC L | 0.43 | 0.09 | 5.0×10^-6^ | Benign  Deleterious | ?+ | 0.87 |
| *CFAP206* | 6: 87415755:A:G  (rs35978098, missense) | 0.005 | 7 | HDLC | 1.77 | 0.38 | 3.6×10^-6^ | Probably damaging, Tolerated | ?+ | 0.99 |
| *SBDS* | 7:66994210:A:G  (rs113993993, splice donor) | 0.005 | 6 | APOC3 | 1.87 | 0.41 | 5.9×10^-6^ | Pathogenic (ClinVar) | ?+ | 0.19 |
| *DEFT1P* | 8:6989658: C:T  (rs797006828, splice-donor) | 0.020 | 17 | VLDLPL XL | 1.16 | 0.24 | 1.2×10^-6^ | Unknown | ?+ | - |
| *NRG1* | 8:32754452:G:T  (rs74942016, missense) | 0.015 | 23 | LDLPL M | 0.94 | 0.21 | 9.0×10^-6^ | Possibly damaging,  Deleterious | ++ | 0.62 |
| *IFNW1* | 9:21141554:G:C  (rs201154486, missense) | 0.005 | 7 | HDLTG XL | 1.75 | 0.38 | 3.7×10^-6^ | Benign,  Deleterious | ++ | 0.43 |
| *GTF3C5* | 9:133054787:C:T  (rs202207045, missense) | 0.007 | 13  13  13 | LDL Friedewald,  Non-HDLC  Total Cholesterol | -1.35  -1.38  -1.35 | 0.28  0.28  0.28 | 1.3×10^-6^  7.3×10^-7^  1.2×10^-6^ | Benign,  Tolerated | --  --  -- | **0.02 (-)**  **0.02 (-)**  0.11 |
| *TET1* | 10:68572915:T:A  (rs12773594, missense) | 0.091 | 136 | LDL M | -0.42 | 0.10 | 8.3×10^-6^ | Benign,  Tolerated | ?- | 0.57 |
| *ANO9* | 11:433867:G:A  (rs12575508, missense) | 0.185 | 277 | HDLC | 0.31 | 0.07 | 5.7×10^-6^ | Benign, Tolerated | ++ | 0.33 |
| *OVCH1* | 12:29464617:G:A  (rs11050243, missense) | 0.191 | 341 | Remnant | 0.28 | 0.06 | 2.9×10^-6^ | Possibly damaging, Deleterious | ++ | 0.83 |
| *FNDC3A* | 13:49201861:A:G  (rs45604939, missense) | 0.064 | 96  69  69  69  69  69 | CHOL CL  HDLPL M  LDLPL L  VLDL S  VLDL XS  VLDLFC S | 0.46  0.47  0.47  0.46  0.47  0.47 | 0.10  0.10  0.10  0.10  0.10  0.10 | 8.8×10-^6^  5.2×10^-6^  5.7×10^-6^  8.0×10^-6^  6.1×10^-6^  7.0×10^-6^ | Benign,  Deleterious | ++  ++  ++  ++  ++  ++ | **0.04 (+)**  0.03 (-)  0.64  0.22  0.45  0.40 |
| *SYNE3* | 14:95417986:G:A  (rs12434757,missense) | 0.650 | 972 | IDLC | 0.24 | 0.05 | 9.0×10^-6^ | Benign,  Tolerated | ++ | 0.64 |
| *GABRG3* | 15:27271593: GAGTC:G  (5’ UTR variant in 2 alternative transcripts) | 0.299 | 448  448 | VLDL XL  VLDLTG XL | -0.38  -0.38 | 0.08  0.08 | 4.9×10^-6^  4.1×10^-6^ | Unknown | ?-  ?- | 0.19  0.21 |
| *LIPC* | 15:58563549:C:T  (rs113298164, missense) | 0.017 | 31 | APOA1 | 0.98 | 0.18 | 7.8×10^-8^ | Probably damaging  Deleterious | ++ | 0.89 |
| *METTL16* | 17: 2475244:T:G  (rs2028600, missense) | 0.068 | 101 | LDLPL L | 0.48 | 0.11 | 8.2×10^-6^ | Benign,  Tolerated | ?- | 0.96 |
| *MARCHF10* | 17:62736189:G:A  (rs147046907, missense) | 0.003 | 5  5  5  5  5  5  5 | IDLFC  IDLPL  LDLPL M  VLDL M  VLDLFC M  VLDLPL M  VLDLPL_XS | -1.99  -2.01  -2.01  -2.18  -2.01  -2.15  -2.19 | 0.45  0.45  0.45  0.45  0.45  0.45  0.45 | 9.3×10^-6^  7.4×10^-6^  7.5×10^-6^  6.9×10^-6^  1.2×10^-6^  1.6×10^-6^  1.1×10^-6^ | Possibly damaging, Deleterious | --  --  --  --  --  --  -- | 0.90  1.00  0.63  0.73  0.67  0.59  0.76 |
| *MYO15B* | 17:75591977:G:T  (rs736522, missense) | 0.252 | 377 | HDLTG S | 0.28 | 0.06 | 1.4×10^-6^ | Possibly damaging  Tolerated | ++ | 0.95 |
| *ZNF274* | 19: 58206912:A:G  (rs45580533, missense) | 0.021 | 20  20 | VLDL L  VLDLTG L | -1.02  -1.05 | 0.23  0.23 | 9.3×10^-6^  5.5×10^-6^ | Benign,  Tolerated | ?- | 0.87  0.81 |
| *LBP* | 20:38346542:C:T  (rs2232580, missense) | 0.052 | 93 | LDL Friedewald | 0.49 | 0.11 | 4.9×10^-6^ | Benign,  Tolerated | ++ | 0.49 |
| *AP000233.3* | 21:25135053:C:T  (rs2829553, splice-donor) | 0.223 | 409 | APOA1 | -0.31 | 0.07 | 2.1×10^-6^ | Low confidence pLoF | ?- | 0.25 |
| *KRTAP13-1* | 21:30396529:G:A  (rs151147550,missense) | 0.007 | 9 | APOC3 | 1.53 | 0.34 | 4.9×10^-6^ | Benign,  Tolerated | ++ | 0.76 |

MAF: Minor allele frequency, Effect: SIFT and Polyphen-2 predictions of the effect of the variant. P SKAT/SKATO: P-value of the SKAT or SKAT-O association test. Dir: Effect direction (for ALT allele) in WES and WGS, respectively. P GWAS: P-value from GWAS analysis, with direction of effect (+/-) indicated for associations with p-value <0.05. Associations with p-value < 0.05 and consistent direction of effect are highlighted with bold.

# Table S5: Lipidome-wide association results for the *LIPC* p.Thr405Met (rs113298164) and *RBM47* p.Ala496-Ala502del (rs564837143) variants.

| ***LIPC* p.Thr405Met (rs113298164) associations** | | | | | |  | ***RBM47* p.Ala496-Ala502del (rs564837143) associations** | | | | | |
| --- | --- | --- | --- | --- | --- | --- | --- | --- | --- | --- | --- | --- |
| **Phenotype** | **N** | **DIR** | **Effect** | **SD** | **P-value** |  | **Phenotype** | **N** | **Dir** | **Effect** | **SD** | **P-value** |
| APOA1_CL | 918 | ++ | 0.980 | 0.182 | 7.8E-08 |  | APOC3_CL | 617 | ?- | -1.938 | 0.412 | 2.5E-06 |
| HDLC_XL_NMR | 748 | ++ | 0.795 | 0.189 | 2.6E-05 |  | VLDLTG_XS_NMR | 748 | ?- | -1.433 | 0.411 | 4.9E-04 |
| HDL_XL_NMR | 748 | ++ | 0.793 | 0.189 | 2.7E-05 |  | TG_CL | 920 | ?- | -1.304 | 0.380 | 6.1E-04 |
| HDLCE_XL_NMR | 748 | ++ | 0.750 | 0.189 | 7.2E-05 |  | HDLTG_XL_NMR | 748 | ?- | -1.244 | 0.411 | 2.5E-03 |
| HDLFC_XL_NMR | 748 | ++ | 0.682 | 0.189 | 3.1E-04 |  | VLDLTG_NMR | 748 | ?- | -1.226 | 0.411 | 2.9E-03 |
| HDLC_CL | 919 | ++ | 0.613 | 0.180 | 6.5E-04 |  | TG_NMR | 748 | ?- | -1.154 | 0.411 | 5.0E-03 |
| HDLPL_XL_NMR | 748 | ++ | 0.633 | 0.189 | 8.1E-04 |  | VLDLTG_S_NMR | 748 | ?- | -1.150 | 0.411 | 5.2E-03 |
| IDLTG_NMR | 748 | ++ | 0.627 | 0.189 | 9.2E-04 |  | VLDLFC_M_NMR | 748 | ?- | -1.131 | 0.411 | 5.9E-03 |
| VLDL_XS_NMR | 748 | ++ | 0.622 | 0.189 | 1.0E-03 |  | VLDL_M_NMR | 748 | ?- | -1.107 | 0.411 | 7.1E-03 |
| HDLFC_L_NMR | 487 | ++ | 0.842 | 0.272 | 2.0E-03 |  | VLDLTG_M_NMR | 748 | ?- | -1.100 | 0.411 | 7.5E-03 |
| VLDLPL_XS_NMR | 748 | ++ | 0.576 | 0.189 | 2.3E-03 |  | APOB_CL | 918 | ?- | -1.011 | 0.380 | 7.9E-03 |
| HDLTG_XL_NMR | 748 | ++ | 0.523 | 0.189 | 5.7E-03 |  | IDLTG_NMR | 748 | ?- | -1.087 | 0.411 | 8.2E-03 |
| HDLC_L_NMR | 748 | ++ | 0.519 | 0.189 | 6.1E-03 |  | VLDLCE_M_NMR | 748 | ?- | -1.041 | 0.411 | 0.011 |
| IDL_NMR | 748 | ++ | 0.515 | 0.189 | 6.4E-03 |  | CHOL_CL_all | 920 | ?- | -0.961 | 0.380 | 0.012 |
| HDL_L_NMR | 748 | ++ | 0.505 | 0.189 | 7.6E-03 |  | VLDL_XS_NMR | 748 | ?- | -1.033 | 0.411 | 0.012 |
| HDL2C_CL | 904 | ++ | 0.468 | 0.180 | 9.2E-03 |  | VLDLPL_M_NMR | 748 | ?- | -1.028 | 0.411 | 0.012 |
| IDLFC_NMR | 748 | ++ | 0.474 | 0.189 | 0.012 |  | VLDL_S_NMR | 748 | ?- | -0.980 | 0.411 | 0.017 |
| CHOL_CL_all | 920 | ++ | 0.447 | 0.180 | 0.013 |  | VLDLPL_XL_NMR | 435 | ?- | -1.019 | 0.452 | 0.024 |
| VLDLTG_XS_NMR | 748 | ++ | 0.466 | 0.189 | 0.014 |  | VLDLTG_XXL_NMR | 549 | ?- | -1.115 | 0.503 | 0.027 |
| HDLCE_L_NMR | 748 | ++ | 0.464 | 0.189 | 0.014 |  | NonHDLC | 919 | ?- | -0.829 | 0.380 | 0.029 |
| VLDLFC_S_NMR | 748 | ++ | 0.457 | 0.189 | 0.016 |  | VLDLCE_L_NMR | 748 | ?- | -0.842 | 0.411 | 0.041 |
| HDL3C_CL | 904 | ++ | 0.425 | 0.180 | 0.018 |  | VLDLC_L_NMR_all | 748 | ?- | -0.840 | 0.411 | 0.041 |
| IDLPL_NMR | 748 | ++ | 0.429 | 0.189 | 0.023 |  | VLDLTG_L_NMR | 748 | ?- | -0.819 | 0.411 | 0.047 |
| VLDLPL_S_NMR | 748 | ++ | 0.426 | 0.189 | 0.024 |  | VLDL_L_NMR | 487 | ?- | -0.818 | 0.411 | 0.047 |
| IDLC_NMR | 748 | ++ | 0.420 | 0.189 | 0.026 |  | VLDLFC_L_NMR | 748 | ?- | -0.802 | 0.411 | 0.05 |
| VLDLC_S_NMR_all | 748 | ++ | 0.419 | 0.189 | 0.027 |  | VLDLPL_L_NMR | 748 | ?- | -0.758 | 0.411 | 0.07 |
| APOA2_CL | 838 | ++ | 0.411 | 0.189 | 0.029 |  | VLDL_XXL_NMR | 748 | ?- | -0.755 | 0.411 | 0.07 |
| HDLTG_S_NMR | 748 | ++ | 0.412 | 0.189 | 0.029 |  | VLDLPL_XS_NMR | 748 | ?- | -0.747 | 0.411 | 0.07 |
| HDLPL_L_NMR | 748 | ++ | 0.410 | 0.189 | 0.030 |  | LDL_friedewald | 894 | ?- | -0.667 | 0.380 | 0.08 |
| CHOL_NMR_all | 748 | ++ | 0.403 | 0.189 | 0.033 |  | VLDLFC_S_NMR | 748 | ?- | -0.694 | 0.411 | 0.09 |
| VLDLCE_M_NMR | 748 | ++ | 0.385 | 0.189 | 0.042 |  | VLDLPL_S_NMR | 748 | ?- | -0.667 | 0.411 | 0.10 |
| VLDLC_M_NMR_all | 476 | ++ | 0.416 | 0.233 | 0.07 |  | IDL_NMR | 748 | ?- | -0.665 | 0.411 | 0.11 |
| HDLC_NMR | 748 | ++ | 0.334 | 0.189 | 0.08 |  | LDL_S_NMR | 748 | ?- | -0.593 | 0.411 | 0.15 |
| HDLCE_M_NMR | 748 | +- | -0.332 | 0.189 | 0.08 |  | Remnant_chol | 894 | ?- | -0.520 | 0.380 | 0.17 |
| LDLFC_L_NMR | 748 | ++ | 0.308 | 0.189 | 0.10 |  | VLDLTG_XL_NMR | 748 | ?- | -0.559 | 0.411 | 0.17 |
| LDLPL_L_NMR | 748 | ++ | 0.303 | 0.189 | 0.11 |  | VLDLC_S_NMR_all | 748 | ?- | -0.550 | 0.411 | 0.18 |
| VLDL_S_NMR | 748 | ++ | 0.301 | 0.189 | 0.11 |  | LDL_M_NMR | 748 | ?- | -0.541 | 0.411 | 0.19 |
| VLDLC_L_NMR_all | 748 | ++ | 0.282 | 0.189 | 0.14 |  | LDLC_S_NMR_all | 748 | ?- | -0.527 | 0.411 | 0.20 |
| LDL_L_NMR | 748 | ++ | 0.281 | 0.189 | 0.14 |  | IDLPL_NMR | 748 | ?- | -0.527 | 0.411 | 0.20 |
| HDLC_M_NMR | 748 | +- | -0.276 | 0.189 | 0.14 |  | VLDL_XL_NMR | 748 | ?- | -0.511 | 0.411 | 0.21 |
| LDL_friedewald | 894 | ++ | 0.257 | 0.180 | 0.15 |  | LDLPL_M_NMR | 748 | ?- | -0.509 | 0.411 | 0.22 |
| VLDLCE_L_NMR | 748 | ++ | 0.262 | 0.189 | 0.17 |  | CHOL_NMR_all | 748 | ?- | -0.490 | 0.411 | 0.23 |
| LDLC_L_NMR_all | 748 | ++ | 0.260 | 0.189 | 0.17 |  | LDLPL_L_NMR | 748 | ?- | -0.486 | 0.411 | 0.24 |
| VLDLFC_L_NMR | 748 | ++ | 0.256 | 0.189 | 0.18 |  | LDL_L_NMR | 748 | ?- | -0.478 | 0.411 | 0.25 |
| VLDL_XL_NMR | 748 | ++ | 0.253 | 0.189 | 0.18 |  | LDLCE_M_NMR | 748 | ?- | -0.464 | 0.411 | 0.26 |
| TG_NMR | 748 | ++ | 0.250 | 0.189 | 0.19 |  | IDLC_NMR | 748 | ?- | -0.446 | 0.411 | 0.28 |
| VLDL_L_NMR | 487 | ++ | 0.354 | 0.272 | 0.19 |  | LDLC_NMR_all | 748 | ?- | -0.428 | 0.411 | 0.30 |
| VLDLTG_XL_NMR | 748 | ++ | 0.233 | 0.189 | 0.22 |  | IDLFC_NMR | 748 | ?- | -0.420 | 0.411 | 0.31 |
| HDL_S_NMR | 748 | +- | -0.215 | 0.189 | 0.26 |  | LDLC_M_NMR_all | 748 | ?- | -0.411 | 0.411 | 0.32 |
| VLDL_XXL_NMR | 748 | ++ | 0.213 | 0.189 | 0.26 |  | VLDLPL_XXL_NMR | 748 | ?- | -0.401 | 0.411 | 0.33 |
| VLDLPL_L_NMR | 748 | ++ | 0.204 | 0.189 | 0.28 |  | HDLCE_M_NMR | 748 | ?+ | 0.376 | 0.411 | 0.36 |
| NonHDLC | 919 | ++ | 0.193 | 0.180 | 0.28 |  | HDLC_M_NMR | 748 | ?+ | 0.368 | 0.411 | 0.37 |
| VLDLFC_M_NMR | 748 | ++ | 0.198 | 0.189 | 0.29 |  | LDLC_L_NMR_all | 748 | ?- | -0.367 | 0.411 | 0.37 |
| HDL_M_NMR | 748 | +- | -0.185 | 0.189 | 0.33 |  | LDLCE_L_NMR | 748 | ?- | -0.340 | 0.411 | 0.41 |
| LDLPL_M_NMR | 748 | ++ | 0.180 | 0.189 | 0.34 |  | HDL_S_NMR | 748 | ?+ | 0.335 | 0.411 | 0.42 |
| VLDLPL_XXL_NMR | 748 | ++ | 0.177 | 0.189 | 0.35 |  | HDLC_NMR | 748 | ?+ | 0.316 | 0.411 | 0.44 |
| Remnant_chol | 894 | -- | -0.168 | 0.180 | 0.35 |  | LDLFC_L_NMR | 748 | ?- | -0.306 | 0.411 | 0.46 |
| VLDLPL_M_NMR | 748 | ++ | 0.171 | 0.189 | 0.37 |  | APOA2_CL | 838 | ?- | -0.279 | 0.411 | 0.50 |
| LDLC_NMR_all | 748 | ++ | 0.165 | 0.189 | 0.38 |  | HDLTG_S_NMR | 748 | ?- | -0.278 | 0.411 | 0.50 |
| VLDLTG_L_NMR | 748 | ++ | 0.160 | 0.189 | 0.40 |  | HDLFC_L_NMR | 487 | ?+ | 0.275 | 0.411 | 0.50 |
| LDLCE_L_NMR | 748 | ++ | 0.160 | 0.189 | 0.40 |  | HDL_L_NMR | 748 | ?+ | 0.263 | 0.411 | 0.52 |
| LDL_S_NMR | 748 | ++ | 0.157 | 0.189 | 0.41 |  | HDLPL_L_NMR | 748 | ?+ | 0.256 | 0.411 | 0.53 |
| LDLC_S_NMR_all | 748 | ++ | 0.147 | 0.189 | 0.44 |  | VLDLC_M_NMR_all | 476 | ?- | -0.618 | 0.998 | 0.54 |
| VLDLTG_XXL_NMR | 549 | ++ | 0.160 | 0.208 | 0.44 |  | HDL3C_CL | 904 | ?- | -0.234 | 0.380 | 0.54 |
| LDL_M_NMR | 748 | ++ | 0.121 | 0.189 | 0.52 |  | HDLPL_XL_NMR | 748 | ?+ | 0.239 | 0.411 | 0.56 |
| APOB_CL | 918 | ++ | 0.115 | 0.182 | 0.53 |  | HDL_M_NMR | 748 | ?+ | 0.215 | 0.411 | 0.60 |
| LDLC_M_NMR_all | 748 | ++ | 0.118 | 0.189 | 0.53 |  | HDLC_L_NMR | 748 | ?+ | 0.194 | 0.411 | 0.64 |
| VLDL_M_NMR | 748 | ++ | 0.115 | 0.189 | 0.54 |  | HDLC_CL | 919 | ?- | -0.174 | 0.380 | 0.65 |
| VLDLTG_S_NMR | 748 | -+ | 0.112 | 0.189 | 0.56 |  | HDL2C_CL | 904 | ?- | -0.138 | 0.380 | 0.72 |
| TG_CL | 920 | ++ | 0.083 | 0.180 | 0.64 |  | HDLCE_XL_NMR | 748 | ?+ | 0.103 | 0.411 | 0.80 |
| VLDLTG_NMR | 748 | ++ | 0.080 | 0.189 | 0.67 |  | HDLCE_L_NMR | 748 | ?+ | 0.103 | 0.411 | 0.80 |
| HDLFC_M_NMR | 748 | +- | 0.061 | 0.189 | 0.75 |  | HDL_XL_NMR | 748 | ?- | -0.087 | 0.411 | 0.83 |
| VLDLTG_M_NMR | 748 | -+ | -0.056 | 0.189 | 0.77 |  | HDLFC_M_NMR | 748 | ?+ | 0.061 | 0.411 | 0.88 |
| HDLPL_M_NMR | 748 | +- | -0.014 | 0.189 | 0.94 |  | HDLPL_M_NMR | 748 | ?+ | 0.039 | 0.411 | 0.92 |
| VLDLPL_XL_NMR | 435 | +- | 0.018 | 0.247 | 0.94 |  | APOA1_CL | 918 | ?+ | 0.023 | 0.380 | 0.95 |
| APOC3_CL | 617 | -+ | 0.013 | 0.222 | 0.95 |  | HDLC_XL_NMR | 748 | ?- | -0.021 | 0.411 | 0.96 |
| LDLCE_M_NMR | 748 | -+ | 0.003 | 0.189 | 0.99 |  | HDLFC_XL_NMR | 748 | ?- | -0.015 | 0.411 | 0.97 |

# Table S6. Significant associations (WES/WGS SKAT meta-analysis p<0.05/19 genes=0.0026) for PTVs and PAVs in known lipid genes.

| GENE |  | Variants | Pheno | MAFs | SINGLEVAR EFFECTs | SINGLEVAR  PVALUEs | P-VALUE | Classification |
| --- | --- | --- | --- | --- | --- | --- | --- | --- |
| *APOB* | PTV | 2:21007222:AT:A (rs1232943044, frameshift)  2:21011037:TGA:T (rs1407451220, frameshift) | LDL Friedewald | 0.0006  0.001 | -0.46  -2.4 | 0.64  0.001 | 0.001 | Likely Pathogenic (PVS1, PM2)  Likely Pathogenic (PVS1, PM2) |
| *APOB* | PTV | 2:21007222:AT:A (rs1232943044, frameshift)  2:21011037:TGA:T (rs1407451220, frameshift) | Non-HDLC | 0.0005  0.001 | -0.64  -2.51 | 0.52  0.0004 | 0.0005 | Likely Pathogenic (PVS1, PM2)  Likely Pathogenic (PVS1, PM2) |
| *APOB* | PTV | 2:21007222:AT:A (rs1232943044, frameshift)  2:21011037:TGA:T (rs1407451220, frameshift) | APOB | 0.0005  0.001 | -1.63  -2.36 | 0.10  0.001 | 0.0006 | Likely Pathogenic (PVS1, PM2)  Likely Pathogenic (PVS1, PM2) |
| *APOB* | PTV | 2:21007222:AT:A (rs1232943044, frameshift)  2:21011037:TGA:T (rs1407451220, frameshift) | VLDLTG S | 0.0007  0.001 | -1.19  -2.32 | 0.23  0.001 | 0.001 | Likely Pathogenic (PVS1, PM2)  Likely Pathogenic (PVS1, PM2) |
| *PCSK9* | PAV | 1:55039974:G:T(rs11591147*)  1:55039995:C:T(rs11583680*)  1:55052701:C:T(rs148195424*) | LDL Friedewald | 0.02  0.05  0.001 | -0.56  -0.10  -1.14 | 0.001  0.37  0.11 | 0.001 | Benign (BP6, BS1, BS2, BP1)  Benign (BA1, BP6, BP4, BP1)  Benign (BS1, BS2, PP3, BP6, BP1) |
| *PCSK9* | PAV | 1:55039974:G:T(rs11591147*)  1:55039995:C:T(rs11583680*)  1:55052701:C:T(rs148195424*) | Non-HDLC | 0.02  0.05  0.001 | -0.61  -0.07  -0.93 | 0.0004  0.55  0.19 | 0.0005 | Benign (BP6, BS1, BS2, BP1)  Benign (BA1, BP6, BP4, BP1)  Benign (BS1, BS2, PP3, BP6, BP1) |
| *PCSK9* | PAV | 1:55039974:G:T(rs11591147*)  1:55039995:C:T(rs11583680*)  1:55052701:C:T(rs148195424*) | Total CHOL | 0.02  0.05  0.001 | -0.64  -0.06  -0.44 | 0.0002  0.63  0.54 | 0.0003 | Benign (BP6, BS1, BS2, BP1)  Benign (BA1, BP6, BP4, BP1)  Benign (BS1, BS2, PP3, BP6, BP1) |
| *LIPC* | PAV | 15:58541794:G:A (rs6078*)  15:58541843:C:T(rs562988299*)  15:58541944:G:A(rs201563586*) 15:58548387:C:T(rs121912502*)  15:58560880:A:C*  15:58563549:C:T(rs113298164*) | APOA1 | 0.03  0.0006  0.006  0.0006  0.05  0.02 | 0.17  0.88  1.80  0.94  0.001  0.98 | 0.20  0.38  0.07  0.35  0.99  7.78E-08 | 1.48E-07 | Benign (BA1, BP4, BP1, BP6)  Likely Benign (PM2, BP1, BP4)  VUS (PM2, PP3, BP1)  VUS (PP3, PM2, PP5, BP1)  -  Benign (BS1, BS2, BP1, PP3, PP5) |
| *LIPC* | PAV | 15:58541794:G:A (rs6078*)  15:58541843:C:T(rs562988299*)  15:58541944:G:A(rs201563586*) 15:58548387:C:T(rs121912502*)  15:58560880:A:C*  15:58563549:C:T(rs113298164*) | HDL CHOL | 0.03  0.0005  0.0005  0.0005  0.05  0.02 | 0.17  -0.51  0.09  0.22  0.04  0.61 | 0.21  0.61  0.93  0.82  0.75  0.001 | 0.002 | Benign (BA1, BP4, BP1, BP6)  Likely Benign (PM2, BP1, BP4)  VUS (PM2, PP3, BP1)  VUS (PP3, PM2, PP5, BP1)  -  Benign (BS1, BS2, BP1, PP3, PP5) |
| *LIPC* | PAV | 15:58541794:G:A (rs6078*)  15:58541843:C:T(rs562988299*)  15:58541944:G:A(rs201563586*) 15:58548387:C:T(rs121912502*)  15:58560880:A:C*  15:58563549:C:T(rs113298164*) | HDLCE XL | 0.03  0.0007  0.0007  0.0007  0.05  0.02 | 0.19  0.76  1.71  2.07  0.11  0.75 | 0.20  0.45  0.09  0.04  0.39  7.22E-05 | 0.0001 | Benign (BA1, BP4, BP1, BP6)  Likely Benign (PM2, BP1, BP4)  VUS (PM2, PP3, BP1)  VUS (PP3, PM2, PP5, BP1)  -  Benign (BS1, BS2, BP1, PP3, PP5) |
| *LIPC* | PAV | 15:58541794:G:A (rs6078*)  15:58541843:C:T(rs562988299*)  15:58541944:G:A(rs201563586*) 15:58548387:C:T(rs121912502*)  15:58560880:A:C*  15:58563549:C:T(rs113298164*) | HDLC XL | 0.03  0.0007  0.0007  0.0007  0.05  0.02 | 0.16  0.77  1.43  1.78  0.07  0.80 | 0.30  0.44  0.15  0.07  0.61  2.65E-05 | 8.12E-05 | Benign (BA1, BP4, BP1, BP6)  Likely Benign (PM2, BP1, BP4)  VUS (PM2, PP3, BP1)  VUS (PP3, PM2, PP5, BP1)  Benign (BS1, BS2, BP1, PP3, PP5) |
| *LIPC* | PAV | 15:58541794:G:A (rs6078*)  15:58541843:C:T(rs562988299*)  15:58541944:G:A(rs201563586*) 15:58548387:C:T(rs121912502*)  15:58560880:A:C*  15:58563549:C:T(rs113298164*) | HDLFC XL | 0.03  0.0007  0.0007  0.0007  0.05  0.02 | 0.04  0.47  0.57  0.87  -0.02  0.68 | 0.79  0.64  0.57  0.38  0.88  0.0003 | 0.002 | Benign (BA1, BP4, BP1, BP6)  Likely Benign (PM2, BP1, BP4)  VUS (PM2, PP3, BP1)  VUS (PP3, PM2, PP5, BP1)  -  Benign (BS1, BS2, BP1, PP3, PP5) |
| *LIPC* | PAV | 15:58541794:G:A (rs6078*)  15:58541843:C:T(rs562988299*)  15:58541944:G:A(rs201563586*) 15:58548387:C:T(rs121912502*)  15:58560880:A:C*  15:58563549:C:T(rs113298164*) | HDLTG XL | 0.03  0.0007  0.0007  0.0007  0.05  0.02 | 0.39  1.13  1.15  0.90  0.03  0.52 | 0.009  0.26  0.25  0.37  0.85  0.006 | 0.001 | Benign (BA1, BP4, BP1, BP6)  Likely Benign (PM2, BP1, BP4)  VUS (PM2, PP3, BP1)  VUS (PP3, PM2, PP5, BP1)  -  Benign (BS1, BS2, BP1, PP3, PP5) |
| *LIPC* | PAV | 15:58541794:G:A (rs6078*)  15:58541843:C:T(rs562988299*)  15:58541944:G:A(rs201563586*) 15:58548387:C:T(rs121912502*)  15:58560880:A:C*  15:58563549:C:T(rs113298164*) | HDL XL | 0.03  0.0007  0.0007  0.0007  0.05  0.02 | 0.15  0.65  0.70  1.19  0.01  0.79 | 0.30  0.52  0.48  0.23  0.92  2.74E-05 | 0.0001 | Benign (BA1, BP4, BP1, BP6)  Likely Benign (PM2, BP1, BP4)  VUS (PM2, PP3, BP1)  VUS (PP3, PM2, PP5, BP1)  -  Benign (BS1, BS2, BP1, PP3, PP5) |
| *LIPC* | PAV | 15:58541794:G:A (rs6078*)  15:58541843:C:T(rs562988299*)  15:58541944:G:A(rs201563586*) 15:58548387:C:T(rs121912502*)  15:58560880:A:C*  15:58563549:C:T(rs113298164*) | IDLTG | 0.03  0.0007  0.0007  0.0007  0.05  0.02 | 0.33  1.07  1.59  1.21  0.06  0.63 | 0.03  0.28  0.11  0.23  0.65  0.0009 | 0.0004 | Benign (BA1, BP4, BP1, BP6)  Likely Benign (PM2, BP1, BP4)  VUS (PM2, PP3, BP1)  VUS (PP3, PM2, PP5, BP1)  -  Benign (BS1, BS2, BP1, PP3, PP5) |
| *CETP* | PAV | 16:56975116:T:C*  16:56981179:G:C(rs5880*)  16:56983407:G:A(rs1800777*) | APOA1 | 0.0006  0.03  0.01 | 0.05  -0.58  -0.57 | 0.99  1.33E-05  0.006 | 5.62E-05 | Likely Benign (PM2, BP4, BP1)  Benign (BA1, BP6, BP1, BP4)  Benign (BA1, BP1, BP4, BP6) |
| *CETP* | PAV | 16:56975116:T:C*  16:56981179:G:C(rs5880*)  16:56983407:G:A(rs1800777*) | HDL2C | 0.0006  0.03  0.01 | -0.26  -0.48  -0.34 | 0.80  0.0003  0.12 | 0.002 | Likely Benign (PM2, BP4, BP1)  Benign (BA1, BP6, BP1, BP4)  Benign (BA1, BP1, BP4, BP6) |
| *CETP* | PAV | 16:56975116:T:C*  16:56981179:G:C(rs5880*)  16:56983407:G:A(rs1800777*) | HDL CHOL | 0.0005  0.04,  0.01 | -0.20  -0.52  -0.45 | 0.84  8.43E-05  0.03 | 0.0005 | Likely Benign (PM2, BP4, BP1)  Benign (BA1, BP6, BP1, BP4)  Benign (BA1, BP1, BP4, BP6) |
| *CETP* | PAV | 16:56975116:T:C*  16:56981179:G:C(rs5880*)  16:56983407:G:A(rs1800777*) | HDLCE M | 0.0007  0.03  0.008 | 0.94  -0.52  -0.78 | 0.35  0.001  0.008 | 0.0007 | Likely Benign (PM2, BP4, BP1)  Benign (BA1, BP6, BP1, BP4)  Benign (BA1, BP1, BP4, BP6) |
| *CETP* | PAV | 16:56975116:T:C*  16:56981179:G:C(rs5880*)  16:56983407:G:A(rs1800777*) | HDLC M | 0.0007  0.03  0.008 | 1.02  -0.57  -0.82 | 0.31  0.0004  0.005 | 0.0002 | Likely Benign (PM2, BP4, BP1)  Benign (BA1, BP6, BP1, BP4)  Benign (BA1, BP1, BP4, BP6) |
| *CETP* | PAV | 16:56975116:T:C*  16:56981179:G:C(rs5880*)  16:56983407:G:A(rs1800777*) | HDLC | 0.0007  0.03  0.008 | -0.02  -0.61  -1.02 | 0.99  0.0002  0.0005 | 3.06E-05 | Likely Benign (PM2, BP4, BP1)  Benign (BA1, BP6, BP1, BP4)  Benign (BA1, BP1, BP4, BP6) |
| *CETP* | PAV | 16:56975116:T:C*  16:56981179:G:C(rs5880*)  16:56983407:G:A(rs1800777*) | HDLFC M | 0.0007  0.03  0.008 | 0.92  -0.48  -0.72 | 0.36  0.003  0.01 | 0.002 | Likely Benign (PM2, BP4, BP1)  Benign (BA1, BP6, BP1, BP4)  Benign (BA1, BP1, BP4, BP6) |
| *CETP* | PAV | 16:56975116:T:C*  16:56981179:G:C(rs5880*)  16:56983407:G:A(rs1800777*) | HDL M | 0.0007  0.03  0.008 | 1.02  -0.57  -0.82 | 0.31  0.0004  0.005 | 0.0002 | Likely Benign (PM2, BP4, BP1)  Benign (BA1, BP6, BP1, BP4)  Benign (BA1, BP1, BP4, BP6) |
| *CETP* | PAV | 16:56975116:T:C*  16:56981179:G:C(rs5880*)  16:56983407:G:A(rs1800777*) | HDLPL M | 0.0007  0.03  0.008 | 0.15  -0.51  -0.95 | 0.88  0.002  0.001 | 0.0003 | Likely Benign (PM2, BP4, BP1)  Benign (BA1, BP6, BP1, BP4)  Benign (BA1, BP1, BP4, BP6) |
| *APOE* | PAV | 19:44907807:G:A(rs201672011*)  19:44907853:T:C(rs769452*)  19:44908822:C:T (rs7412*) | APOB | 0.0006  0.009  0.04 | 0.11  0.31  -0.49 | 0.91  0.21  0.0001 | 0.0003 | VUS (PM2, PP2, PP5, BP4)  Likely Benign (PP2, BP4, BS2)  VUS (PM1, PP2, PP3, BA1, PP5) |
| *APOE* | PAV | 19:44907853:T:C (rs201672011*)  19:44908822:C:T(rs7412*) | HDLD | 0.005  0.03 | -0.37  1.16 | 0.60  0.0005 | 0.0007 | Likely Benign (PP2, BS2, BP4)  VUS (PM2, PP2, PP3, PP5, BA1) |
| *APOE* | PAV | 19:44907807:G:A(rs201672011*)  19:44907853:T:C(rs769452*)  19:44908822:C:T (rs7412*) | LDLCE L | 0.0007  0.009  0.03 | -2.43  0.26  -0.57 | 0.01  0.35  0.00012 | 0.0002 | VUS (PM2, PP2, PP5, BP4),  Likely Benign (PP2, BP4, BS2)  VUS (PM1, PP2, PP3, BA1, PP5) |
| *APOE* | PAV | 19:44907807:G:A(rs201672011*)  19:44907853:T:C(rs769452*)  19:44908822:C:T (rs7412*) | LDLCE M | 0.0007  0.009  0.03 | -0.61  0.15  -0.53 | 0.54  0.59  0.0005 | 0.001 | VUS (PM2, PP2, PP5, BP4)  Likely Benign (PP2, BP4, BS2)  VUS (PM1, PP2, PP3, BA1, PP5) |
| *APOE* | PAV | 19:44907807:G:A(rs201672011*)  19:44907853:T:C(rs769452*)  19:44908822:C:T (rs7412*) | LDLC L | 0.0007  0.009  0.03 | -2.44  0.30  -0.58 | 0.01  0.29  0.0001 | 0.0002 | VUS (PM2, PP2, PP5, BP4)  Likely Benign (PP2, BP4, BS2)  VUS (PM1, PP2, PP3, BA1, PP5) |
| *APOE* | PAV | 19:44907807:G:A(rs201672011*)  19:44907853:T:C(rs769452*)  19:44908822:C:T (rs7412*) | LDLC M | 0.0007  0.009  0.03 | -1.08  0.25  -0.58 | 0.28  0.38  0.0001 | 0.0003 | VUS (PM2, PP2, PP5, BP4)  Likely Benign (PP2, BP4, BS2)  VUS (PM1, PP2, PP3, BA1, PP5) |
| *APOE* | PAV | 19:44907807:G:A(rs201672011*)  19:44907853:T:C(rs769452*)  19:44908822:C:T (rs7412*) | LDLC | 0.0007  0.009  0.03 | -2.01  0.26  -0.57 | 0.04  0.35  0.0002 | 0.0003 | VUS (PM2, PP2, PP5, BP4)  Likely Benign (PP2, BP4, BS2)  VUS (PM1, PP2, PP3, BA1, PP5) |

PTV= Protein truncating variant. PAV= Protein altering variant. SINGLEVAR EFFECTs= Effect sizes of single variants. P-value=P-value for SKAT meta-analysis gene test. *indicates nonsynonymous missense mutation. Classification = Variant classification according to ACMG guidelines (*Richards et al. 2015*), using automated classification provided by VarSome (*Kopanos et al, 2019*):
PVS1: Pathogenic, Very Strong.
PS1-3: Pathogenic, Strong
PM1-5: Pathogenic, Moderate.
PP2-5: Pathogenic, Supporting.
BA1: Benign, Stand Alone
BP1-7: Benign, Supporting
BS1-3: Benign, Strong

The studied genes include genes causing

- hypercholesteremia (*PCSK9* (MIM:607786)*, LDLR* (MIM:606945)*, APOB* (MIM:107730)*, APOE* (MIM:107741), *LPA* (MIM:152200)*, LDLRAP1* (MIM:605747)*, LIPA* (MIM:613497), and *CYP7A1* (MIM:118455),
- monogenic hypertriglyceridemia (*LPL (MIM*:*609708), APOC2* (MIM:608083)*, GPIHBP1* (MIM: 612757)*, APOA5* (MIM:606368) and *LMF1* (MIM:611761)),
- genetic disorders of HDL metabolism (*ABCA1* (MIM:600046), *LCAT* (MIM:606967), *APOA1* (MIM:107680)*, ANGPTL3* (MIM:604774)*, CETP* (MIM:118470), *LIPC* (MIM:151670)*,* and *LIPG* (MIM: 603684))

as described in Musunuru et al., 2015.

# Table S7: Replication p-values for the single variant meta-analysis lead variants in the GLGC GWAS, UKBB WES, and Lipid WES.

|  |  |  |  |  |  | **CHOL** | | | **HDL** | | | **LDL** | | | **TG** | | | | **Non-HDL** | | | **TG:HDL** | | **apoA** | | **apoB** |  |
| --- | --- | --- | --- | --- | --- | --- | --- | --- | --- | --- | --- | --- | --- | --- | --- | --- | --- | --- | --- | --- | --- | --- | --- | --- | --- | --- | --- |
| **CHRPOS** | **RS** | **GENE** | **REF** | **ALT** | **AF** | **GLGC** | **UKBB** | **Hindy** | **GLGC** | **UKBB** | **Hindy** | **GLGC** | **UKBB** | **Hindy** | | **GLGC** | **UKBB** | **Hindy** | | **GLGC** | **Hindy** | | **Hindy** | | **UKBB** | **UKBB** | |
| 1:19448881 | rs79308175 | *CAPZB* | G | A | 0.013 | 0.195 | NA | 0.101 | 0.725 | NA | 0.619 | 0.208 | NA | 0.177 | | 0.186 | NA | 0.585 | | 0.158 | 0.052 | | 0.643 | | NA | NA | |
| 4:40432687 | rs564837143 | *RBM47* | AGCGGCTGCGGCGGCTGCGGCC | A | 0.005 | 0.696 | NA | 0.673 | 0.866 | NA | 0.167 | 0.592 | NA | 0.923 | | 0.910 | NA | 0.645 | | 0.193 | 0.999 | | 0.283 | | NA | NA | |
| 4:139045355 | rs144899070 | *NOCT* | G | A | 0.005 | 0.015 | NA | 0.805 | 0.396 | NA | 0.980 | 0.018 | NA | 0.395 | | 0.007 | NA | 0.706 | | 0.020 | 0.630 | | 0.750 | | NA | NA | |
| 5:123023945 | rs451195 | *PPIC* | T | C | 0.156 | 0.110 | NA | 0.076 | 2.1E-07 | NA | 0.187 | 0.127 | NA | 0.065 | | 0.001 | NA | 0.367 | | 0.332 | 0.078 | | 0.266 | | NA | NA | |
| 6:87415755 | rs35978098 | *CFAP206* | A | G | 0.005 | 0.011 | 0.841 | 0.420 | 0.886 | 0.784 | 0.482 | 0.105 | 0.718 | 0.543 | | 0.095 | 0.870 | 0.850 | | 0.120 | 0.434 | | 0.980 | | 0.697 | 0.952 | |
| 7:66994210 | rs113993993 | *SBDS* | A | G | 0.005 | 0.617 | 0.135 | 0.185 | 0.295 | 0.446 | 0.488 | 0.957 | 0.209 | 0.170 | | 0.338 | 0.833 | 0.480 | | 0.489 | 0.118 | | 0.302 | | 0.458 | 0.278 | |
| 8:6989658 | rs797006828 | *DEFT1P* | C | T | 0.02 | NA | NA | NA | NA | NA | NA | NA | NA | NA | | NA | NA | NA | | NA | NA | | NA | | NA | NA | |
| 8:32754452 | rs74942016 | *NRG1* | G | T | 0.015 | 0.838 | 0.372 | 0.410 | 0.840 | 0.657 | 0.603 | 0.468 | 0.294 | 0.413 | | 0.132 | 0.852 | 0.848 | | 0.995 | 0.304 | | 0.666 | | 0.903 | 0.297 | |
| 9:21141554 | rs201154486 | *IFNW1* | G | C | 0.005 | 0.106 | NA | 0.267 | 0.588 | NA | 0.314 | 0.013 | NA | 0.185 | | 0.349 | NA | 0.251 | | 0.038 | 0.178 | | 0.201 | | NA | NA | |
| 9:133054787 | rs202207045 | *GTF3C5* | C | T | 0.007 | 0.862 | NA | 0.375 | 0.079 | NA | 0.349 | 0.770 | NA | 0.125 | | 0.742 | NA | 0.943 | | 0.919 | 0.199 | | 0.742 | | NA | NA | |
| 10:68572915 | rs12773594 | *TET1* | T | A | 0.091 | 0.007 | NA | 0.901 | 0.626 | NA | 0.762 | 0.039 | NA | 0.400 | | 0.124 | NA | 0.853 | | 0.056 | 0.596 | | 0.668 | | NA | NA | |
| 11:433867 | rs12575508 | *ANO9* | G | A | 0.185 | 0.203 | NA | 0.182 | 0.725 | NA | 0.160 | 0.115 | NA | 0.580 | | 0.802 | NA | 0.359 | | 0.037 | 0.512 | | 0.818 | | NA | NA | |
| 12:29464617 | rs11050243 | *OVCH1* | G | A | 0.191 | 0.040 | NA | 0.630 | 0.129 | NA | 0.279 | 0.296 | NA | 0.712 | | 0.039 | NA | 0.743 | | 0.738 | 0.378 | | 0.396 | | NA | NA | |
| 13:49201861 | rs45604939 | *FNDC3A* | A | G | 0.064 | 0.437 | 0.654 | 0.352 | 0.025 | 0.012 | 0.774 | 0.092 | 0.893 | 0.360 | | 0.999 | 0.360 | 0.928 | | 0.169 | 0.277 | | 0.817 | | 0.079 | 0.473 | |
| 14:95417986 | rs12434757 | *SYNE3* | G | A | 0.65 | 0.531 | NA | 0.987 | 0.222 | NA | 0.704 | 0.838 | NA | 0.733 | | 0.448 | NA | 0.564 | | 0.506 | 0.816 | | 0.567 | | NA | NA | |
| 15:27271593 | rs61549890 | *GABRG3* | GAGTC | G | 0.299 | NA | NA | 0.244 | NA | NA | 0.032 | NA | NA | 0.724 | | NA | NA | 0.994 | | NA | 0.742 | | 0.345 | | NA | NA | |
| 15:58563549 | rs113298164 | *LIPC* | C | T | 0.017 | 1.9E-31 | 1.4E-07 | 0.078 | 3.9E-135 | 1.5E-39 | 1.0E-15 | 0.937 | 0.376 | 0.205 | | 5.9E-33 | 1.7E-12 | 0.040 | | 0.185 | 0.231 | | 0.126 | | 9.3E-46 | 0.110 | |
| 17:2475244 | rs2028600 | *METTL16* | T | G | 0.068 | 0.584 | NA | 0.789 | 0.058 | NA | 0.465 | 0.305 | NA | 0.749 | | 0.697 | NA | 0.875 | | 0.334 | 0.869 | | 0.746 | | NA | NA | |
| 17:62736189 | rs147046907 | *MARCH10* | G | A | 0.003 | 0.388 | NA | 0.549 | 0.343 | NA | 0.284 | 0.348 | NA | 0.610 | | 0.877 | NA | 0.179 | | 0.657 | 0.403 | | 0.253 | | NA | NA | |
| 17:75591977 | rs736522 | *MYO15B* | G | T | 0.252 | 0.457 | NA | 0.209 | 0.110 | NA | 0.479 | 0.800 | NA | 0.324 | | 0.229 | NA | 0.328 | | 0.100 | 0.259 | | 0.844 | | NA | NA | |
| 19:58206912 | rs45580533 | *ZNF274* | A | G | 0.021 | 2.4E-14 | NA | 0.001 | 0.681 | NA | 0.225 | 3.0E-13 | NA | 0.003 | | 0.003 | NA | 0.716 | | 2.0E-16 | 0.011 | | 0.786 | | NA | NA | |
| 20:38346542 | rs2232580 | *LBP* | C | T | 0.052 | 0.893 | NA | 0.501 | 0.968 | NA | 0.964 | 0.964 | NA | 0.714 | | 0.617 | NA | 0.863 | | 0.846 | 0.565 | | 0.746 | | NA | NA | |
| 21:25135053 | rs2829553 | *AP000233.3* | C | T | 0.223 | 0.581 | NA | NA | 0.438 | NA | NA | 0.492 | NA | NA | | 0.543 | NA | NA | | 0.581 | NA | | NA | | NA | NA | |
| 21:30396529 | rs151147550 | *KRTAP13-1* | G | A | 0.007 | 0.138 | NA | 0.036 | 0.577 | NA | 0.512 | 0.052 | NA | 0.014 | | 0.300 | NA | 0.875 | | 0.591 | 0.057 | | 0.760 | | NA | NA | |

Lipid associations for available standard lipids from GLGC GWAS (Graham et al. 2021), UKBB WES (Backman et al. 2021), and Lipid WES (Hindy et al. 2022). Red shades indicate that the variant had positive effect direction for the minor allele; blue shades that the variant had negative effect for the minor allele. Dark shades, p<0.05/27/8; medium shades, p<0.05/27; light shades, p<0.05.

# Table S8: Single variant score test association results for PTVs and PAVs within the lead genes from the WES+WGS SKAT or VT meta-analysis.

|  | **WES+WGS meta-analysis** | | | | |  | |  | **WES+WGS meta** | | |  | | **GWAS** | | | | |
| --- | --- | --- | --- | --- | --- | --- | --- | --- | --- | --- | --- | --- | --- | --- | --- | --- | --- | --- |
| **GENE** | **Type** | **Pheno** | **P-value** | **Variants** |  | | **MAF** | | **MAC** | **Effect** | **P-value** |  | **N** | | **AF** | **Effect** | **P-value** |  |
| **SKAT** |  |  |  |  |  | |  | |  |  |  |  |  | |  |  |  |  |
| *DEFT1P/ DEFT1P2* | PTV | VLDLPL XL | 1.23E-06 | 8:6989658:C:T (rs797006828, splice donor variant ) |  | | 0.020 | | 17 | 1.157 | 1.23E-06 |  |  | |  |  |  |  |
| *SBDS* | PTV | apoC-III | 5.37E-06 | 7:66994210:A:G (rs113993993, splice donor variant); |  | | 0.005 | | 6 | 1.865 | 5.86E-06 |  | 2982 | | 0.008 | 0.22 | 0.19 |  |
|  |  |  |  | 7:66994286:T:A (rs113993991, stop gain) |  | | 0.001 | | 1 | 0.603 | 0.55 |  |  | |  |  |  |  |
| *LIPC* | PAV | apoA1 | 1.48E-07 | 15:58541794:G:A (rs6078*); |  | | 0.032 | | 58 | 0.171 | 0.20 |  | 3759 | | 0.058 | 0.04 | 0.41 |  |
|  |  |  |  | 15:58541843:C:T (rs562988299*); |  | | 0.001 | | 1 | 0.877 | 0.38 |  |  | |  |  |  |  |
|  |  |  |  | 15:58541944:G:A (rs201563586*); |  | | 0.001 | | 1 | 1.794 | 0.07 |  | 3759 | | 0.002 | 0.12 | 0.67 |  |
|  |  |  |  | 15:58548387:C:T (rs121912502*); |  | | 0.001 | | 1 | 0.937 | 0.35 |  | 3759 | | 0.0002 | 2.56 | 0.04** |  |
|  |  |  |  | 15:58560880:A:C (rs3829462*); |  | | 0.045 | | 83 | 0.001 | 0.99 |  | 3759 | | 0.046 | 0.12 | 0.02 |  |
|  |  |  |  | 15:58563549:C:T (rs113298164*) |  | | 0.017 | | 31 | 0.980 | 7.77E-08 |  | 3759 | | 0.058 | 0.01 | 0.89 |  |
| *GTF3C5* | PAV | Non-HDLC | 6.86E-07 | 9:133042147:C:T (-) ; |  | | 0.001 | | 1 | 0.596 | 0.55 |  |  | |  |  |  |  |
|  |  |  |  | 9:133043731:T:C (rs189383196, start lost, missense); |  | | 0.004 | | 8 | 0.311 | 0.38 |  | 4636 | | 0.010 | 0.14 | 0.17 |  |
|  |  |  |  | 9:133043838:C:A (rs150056568)*; |  | | 0.001 | | 2 | 0.017 | 0.98 |  | 4636 | | 0.003 | 0.18 | 0.33 |  |
|  |  |  |  | 9:133054459:A:G (rs369889499*); |  | | 0.001 | | 2 | -0.392 | 0.58 |  | 4636 | | 0.001 | -0.08 | 0.84 |  |
|  |  |  |  | 9:133054733:A:G (rs1302997790)*; |  | | 0.001 | | 1 | 0.840 | 0.40 |  |  | |  |  |  |  |
|  |  |  |  | 9:133054787:C:T (rs202207045*); |  | | 0.007 | | 13 | -1.382 | 7.26E-07 |  | 4636 | | 0.008 | -0.28 | 0.02 |  |
|  |  |  |  | 9:133056848:G:A (rs637435)*; |  | | 0.001 | | 1 | -0.596 | 0.55 |  |  | |  |  |  |  |
|  |  |  |  | 9:133057819:T:C (rs111893665)* |  | | 0.001 | | 1 | -0.120 | 0.90 |  | 4636 | | 0.001 | 0.17 | 0.60 |  |
| *GTF3C5* | PAV | LDL Friedewald | 9.27E-07 | 9:133042147:C:T (-) ; |  | | 0.001 | | 1 | 0.642 | 0.52 |  |  | |  |  |  |  |
|  |  |  |  | 9:133043731:T:C (rs189383196, start lost, missense); |  | | 0.003 | | 6 | -0.309 | 0.45 |  | 4584 | | 0.010 | 0.16 | 0.11 |  |
|  |  |  |  | 9:133043838:C:A (rs150056568)*; |  | | 0.001 | | 2 | 0.100 | 0.89 |  | 4584 | | 0.003 | 0.19 | 0.31 |  |
|  |  |  |  | 9:133054459:A:G (rs369889499*); |  | | 0.001 | | 2 | -0.302 | 0.67 |  | 4584 | | 0.001 | -0.01 | 0.99 |  |
|  |  |  |  | 9:133054733:A:G (rs1302997790)*; |  | | 0.001 | | 1 | 0.956 | 0.34 |  |  | |  |  |  |  |
|  |  |  |  | 9:133054787:C:T (rs202207045*); |  | | 0.007 | | 13 | -1.350 | 1.31E-06 |  | 4584 | | 0.008 | -0.28 | 0.02 |  |
|  |  |  |  | 9:133056848:G:A (rs637435)*; |  | | 0.001 | | 1 | -0.690 | 0.49 |  |  | |  |  |  |  |
|  |  |  |  | 9:133057819:T:C (rs111893665)* |  | | 0.001 | | 1 | -0.131 | 0.90 |  | 4584 | | 0.001 | 0.07 | 0.83 |  |
| *GTF3C5* | PAV | CHOL CL | 1.30E-06 | 9:133042147:C:T (-) ; |  | | 0.001 | | 1 | 1.113 | 0.27 |  |  | |  |  |  |  |
|  |  |  |  | 9:133043731:T:C (rs189383196, start lost, missense); |  | | 0.004 | | 8 | 0.253 | 0.48 |  | 4653 | | 0.010 | 0.12 | 0.23 |  |
|  |  |  |  | 9:133043838:C:A (rs150056568)*; |  | | 0.001 | | 2 | -0.003 | 1.00 |  | 4653 | | 0.003 | 0.18 | 0.35 |  |
|  |  |  |  | 9:133054459:A:G (rs369889499*); |  | | 0.001 | | 2 | -0.344 | 0.63 |  | 4653 | | 0.001 | -0.10 | 0.78 |  |
|  |  |  |  | 9:133054733:A:G (rs1302997790)*; |  | | 0.001 | | 1 | 0.671 | 0.50 |  |  | |  |  |  |  |
|  |  |  |  | 9:133054787:C:T (rs202207045*); |  | | 0.007 | | 13 | -1.353 | 1.23E-06 |  | 4653 | | 0.008 | -0.18 | 0.11 |  |
|  |  |  |  | 9:133056848:G:A (rs637435)*; |  | | 0.001 | | 1 | -0.885 | 0.38 |  |  | |  |  |  |  |
|  |  |  |  | 9:133057819:T:C (rs111893665)* |  | | 0.001 | | 1 | 0.248 | 0.80 |  | 4653 | | 0.001 | 0.31 | 0.32 |  |
| *TRMT5* | PAV | VLDLPL XS | 7.87E-07 | 14:60975180:G:A (rs147405788*); |  | | 0.001 | | 2 | -1.575 | 0.03 |  | 1466 | | 0.002 | -0.08 | 0.82 |  |
|  |  |  |  | 14:60975182:T:C (-)*; |  | | 0.001 | | 1 | -1.386 | 0.17 |  |  | |  |  |  |  |
|  |  |  |  | 14:60975636:G:A (rs45604437*); |  | | 0.007 | | 10 | -1.094 | 5.76E-04 |  | 1466 | | 0.003 | -0.23 | 0.53 |  |
|  |  |  |  | 14:60979242:T:C (rs138139551*); |  | | 0.002 | | 3 | -0.599 | 0.30 |  | 1466 | | 0.003 | -0.04 | 0.91 |  |
|  |  |  |  | 14:60979257:T:C (rs191207997*); |  | | 0.001 | | 1 | -0.713 | 0.48 |  | 1466 | | 0.001 | -0.36 | 0.53 |  |
|  |  |  |  | 14:60979303:C:G (rs1285433520)*; |  | | 0.001 | | 1 | 0.400 | 0.69 |  |  | |  |  |  |  |
|  |  |  |  | 14:60979401:G:A (rs746471581*); |  | | 0.001 | | 1 | 1.362 | 0.17 |  |  | |  |  |  |  |
|  |  |  |  | 14:60979429:T:A (rs115400838)* |  | | 0.007 | | 10 | -1.211 | 1.59E-04 |  | 1466 | | 0.013 | -0.05 | 0.75 |  |
| *RBM47* | PAV | apoC-III | 1.33E-06 | 4:40426074:C:T (rs35529250)*; |  | | 0.001 | | 1 | -1.020 | 0.31 |  | 2982 | | 0.005 | 0.06 | 0.76 |  |
|  |  |  |  | 4:40432687:AGCGGCTGCGGCGGCTGCGGCC:A (rs564837143, in frame deletion) |  | | 0.005 | | 6 | -1.938 | 2.50E-06 |  | 2982 | | 0.008 | 0.26 | 0.08 |  |
|  |  |  |  | 4:40438508:C:T (rs373211767*) |  | | 0.002 | | 2 | 0.817 | 0.25 |  | 2982 | | 0.001 | 0.52 | 0.23 |  |
| **VT** |  |  |  |  |  | |  | |  |  |  |  |  | |  |  |  |  |
| *RYR3* | PAV | VLDLTG XS | 2.08E-06 | 15:33579984:A:G (rs146838868)* |  | | 0.008 | | 12 | 0.318 | 0.28 |  | 2598 | | 0.018 | 0.060 | 0.58 |  |
|  |  |  |  | 15:33601461:G:A (rs979125271)* |  | | 0.0007 | | 1 | 1.258 | 0.21 |  |  | |  |  |  |  |
|  |  |  |  | 15:33629953:A:G (rs2229119)* |  | | 0.007 | | 11 | 0.812 | 7.42E-03 |  | 2598 | | 0.007 | -0.203 | 0.22 |  |
|  |  |  |  | 15:33634625:G:A (rs749982964)* |  | | 0.0007 | | 1 | 0.612 | 0.54 |  |  | |  |  |  |  |
|  |  |  |  | 15:33649129:G:A (rs1309424472)* |  | | 0.0007 | | 1 | 1.064 | 0.29 |  |  | |  |  |  |  |
|  |  |  |  | 15:33652758:A:G (rs571025757)* |  | | 0.0007 | | 1 | 1.091 | 0.27 |  |  | |  |  |  |  |
|  |  |  |  | 15:33663642:A:C (rs201524472)* |  | | 0.0007 | | 1 | 0.674 | 0.50 |  |  | |  |  |  |  |
|  |  |  |  | 15:33699813:A:G (rs370232598)* |  | | 0.0007 | | 1 | -0.359 | 0.72 |  |  | |  |  |  |  |
|  |  |  |  | 15:33706919:G:T (rs766431661)* |  | | 0.0007 | | 1 | -0.146 | 0.88 |  | 2598 | | 0.0005 | 0.801 | 0.26 |  |
|  |  |  |  | 15:33728863:C:T (rs781065862)* |  | | 0.0007 | | 1 | 1.952 | 0.05 |  |  | |  |  |  |  |
|  |  |  |  | 15:33731519:A:G (rs200294137)* |  | | 0.0007 | | 1 | -2.065 | 0.04 |  |  | |  |  |  |  |
|  |  |  |  | 15:33739987:C:G (rs41279214)* |  | | 0.0013 | | 2 | 0.414 | 0.56 |  | 2598 | | 0.002 | -0.068 | 0.81 |  |
|  |  |  |  | 15:33748495:G:A (rs376553827)* |  | | 0.0007 | | 1 | 0.784 | 0.43 |  |  | |  |  |  |  |
|  |  |  |  | 15:33750262:A:G (rs1429954855)* |  | | 0.0007 | | 1 | -0.361 | 0.72 |  |  | |  |  |  |  |
|  |  |  |  | 15:33772108:C:T (rs776121340)* |  | | 0.0007 | | 1 | 1.592 | 0.11 |  |  | |  |  |  |  |
|  |  |  |  | 15:33773591:C:T (rs764112773)* |  | | 0.0007 | | 1 | -1.091 | 0.27 |  |  | |  |  |  |  |
|  |  |  |  | 15:33780327:C:G (rs61996335)* |  | | 0.005 | | 8 | 0.792 | 0.03 |  | 2598 | | 0.004 | -0.276 | 0.22 |  |
|  |  |  |  | 15:33785748:G:A (rs200830195)* |  | | 0.005 | | 8 | 0.092 | 0.80 |  | 2598 | | 0.005 | -0.122 | 0.54 |  |
|  |  |  |  | 15:33788395:C:G (rs775224723)* |  | | 0.0007 | | 1 | -0.545 | 0.59 |  |  | |  |  |  |  |
|  |  |  |  | 15:33801900:T:C (rs150028316)* |  | | 0.0007 | | 1 | 0.534 | 0.59 |  | 2598 | | 0.005 | -0.089 | 0.65 |  |
|  |  |  |  | 15:33821528:A:G (rs146201205)* |  | | 0.009 | | 14 | 0.861 | 1.39E-03 |  | 2598 | | 0.009 | 0.064 | 0.66 |  |
|  |  |  |  | 15:33835049:A:C (rs202181075)* |  | | 0.009 | | 14 | 0.670 | 0.01 |  | 2598 | | 0.014 | 0.052 | 0.68 |  |
|  |  |  |  | 15:33838411:T:C (rs747261018)* |  | | 0.0007 | | 1 | -0.015 | 0.99 |  |  | |  |  |  |  |
|  |  |  |  | 15:33841953:G:A (rs201791791)* |  | | 0.0013 | | 2 | -0.355 | 0.62 |  | 2598 | | 0.0008 | -0.692 | 0.17 |  |
|  |  |  |  | 15:33857882:G:A (rs182257230)* |  | | 0.014 | | 21 | -0.049 | 0.82 |  | 2598 | | 0.015 | -0.139 | 0.21 |  |
| *MARCHF10* | PAV | VLDLPL XS | 2.24E-06 | 17:62736189:G:A (rs147046907)* |  | | 0.003 | | 5 | -2.186 | 1.07E-06 |  | 2598 | | 0.005 | -0.064 | 0.76 |  |
|  |  |  |  | 17:62736691:T:G (rs140716269)* |  | | 0.005 | | 7 | 0.229 | 0.55 |  | 2598 | | 0.004 | -0.030 | 0.89 |  |
|  |  |  |  | 17:62737066:A:G (rs1329816461)* |  | | 0.0007 | | 1 | 0.368 | 0.71 |  |  | |  |  |  |  |
|  |  |  |  | 17:62746934:C:T (rs916315847)* |  | | 0.0007 | | 1 | -2.213 | 0.03 |  |  | |  |  |  |  |
|  |  |  |  | 17:62801665:T:C (rs199705946)* |  | | 0.003 | | 4 | -0.919 | 0.07 |  | 2598 | | 0.005 | -0.046 | 0.83 |  |

P-value: P-value for gene aggregate test (Liu’s method used for SKAT) or for the single variant analysis. Variants: Variants within the gene that contributed to the gene aggregate meta-analysis (i.e. all PTVs or PAVs within the gene, with MAF below the threshold for the VT test. Annotation includes chr:pos:REF:ALT alleles, rs number if known, and consequence. *indicates nonsynonymous missense mutation. MAF: Minor allele frequency for each variant; MAC: minor allele count. Effect: beta effect size estimate from score test meta-analysis. GWAS: N, Allele frequency (AF), Effect size and P-value for the variants in the non-overlapping GWAS data. P-values <0.05 (not corrected for multiple testing) are highlighted in bold.

VLDLPL XS/XL: Phospholipid in extra small/large VLDL, VLDLTG XS: Triglycerides in extra small VLDL, apoC-III: Serum Apolipoprotein C-III, apoA1: Serum apolipoprotein A-I [mg/dl], CHOL CL: Total Cholesterol, LDL Friedewald: LDL cholesterol calculated with Friedewald equation. Non-HDLC: Non-HDL cholesterol.
**LIPC rs121912502 was imputed with low imputation quality, INFO 0.37

# Table S9: WES-WGS SKAT meta-analysis on coronary artery disease (CAD), Strokes, cardiovascular disease (CVD; defined as CAD or stroke), and diabetic kidney disease (DKD) for the lead genes.

| GENE | N VAR | AVG_AF | P CVD | P CAD | P Stroke | P DKD |
| --- | --- | --- | --- | --- | --- | --- |
| PAV |  |  |  |  |  |  |
| *RBM47* | 4 | 0.002 | 0.444 | 1.000 | 0.148 | 0.332 |
| *CYP3A43* | 7 | 0.005 | 0.360 | 0.091 | 0.675 | 0.004 |
| *GTF3C5* | 9 | 0.002 | 0.066 | 0.270 | 0.465 | 0.844 |
| *AKAP3* | 10 | 0.015 | 0.733 | 0.599 | 0.827 | 0.822 |
| *TRMT5* | 8 | 0.003 | 0.307 | 0.567 | 0.610 | 0.736 |
| *LIPC* | 6 | 0.017 | 0.325 | 0.726 | 0.508 | 0.658 |
| *RYR3* | 29 | 0.003 | 0.36 | 0.39 | 0.77 | 0.03 |
| *MARCH10* | 7 | 0.003 | 0.53 | 0.56 | 0.21 | 0.95 |
| PTV |  |  |  |  |  |  |
| *PTGER3* | 1 | 0.010 | 0.672 | 0.278 | 0.157 | 0.476 |
| *SBDS* | 3 | 0.002 | 0.595 | 0.276 | 0.536 | 0.061 |
| *DEFT1P/ DEFT1P2* | 1 | 0.024 | 0.448 | 0.197 | 0.707 | 0.265 |

N Var: number of PAV/ PTV variants with MAF ≤ 5% within the gene. AVG_AF: average allele frequency of the variants. P CVD/ CAD/ Stroke/ DKD: WES-WGS SKAT meta-analysis P-value.

Table S10: Lead gene associations with cardiovascular outcomes in the UK Biobank WES by Backman et al. 2021. All nominally significant associations are shown.

| Name | effect_allele | Trait | Model | odds_ratio | ci_lower | ci_upper | p_value | effect_allele_frequency | standard_error |
| --- | --- | --- | --- | --- | --- | --- | --- | --- | --- |
| *GTF3C5* | M1.1 | CAD | ADD-WGR-FIRTH | 1.89 | 1.26 | 2.84 | 0.0022 | 0.00040 | 0.208 |
| *RYR3* | M1.1 | MI | ADD-WGR-FIRTH | 1.76 | 1.19 | 2.61 | 0.0046 | 0.00060 | 0.200 |
| *CYP3A43* | M1.1 | CAD | ADD-WGR-FIRTH | 1.32 | 1.04 | 1.67 | 0.021 | 0.0013 | 0.120 |

M1.1: putative loss-of-function variants with MAF ≤ 1%. CAD: Coronary artery disease, GWAS catalog GCST90085452. MI: myocardial infarction, GWAS catalog GCST90085481. Associations were sought also for stroke, GWAS catalog GCST90081649.

# Table S11: Variant association with “Diseases of the circulatory system” phenotypes in the FinnGen GWAS data.

| **GENE: Pheno** | **VARs** | **Effect** | **rsID** | **MAFs** | **EFF** | **P-values** | **SIFT** | **PolyPhen** | **FinEnr** | **phenotype** | **beta** | **p-value** | **N** |
| --- | --- | --- | --- | --- | --- | --- | --- | --- | --- | --- | --- | --- | --- |
| DEFT1P/ DEFT1P2:  VLDLPL XL | 8:6989658:C:T | Splice donor | rs797006828 | 0.020 | 1.157 | 1.23E-06 | - | - |  |  |  |  |  |
| SBDS: APOC3 | 7:66994210:A:G | Splice donor | rs113993993 | 0.005 | 1.865 | 5.86E-06 | - | - | 2.27 | [Hypertension complicating pregnancy, childbirth, and the puerperium](http://r4.finngen.fi/pheno/I9_HYPTENSPREG) | 0.26 | 1.60E-02 | 6120 / 94241 |
|  |  |  |  |  |  |  |  |  |  | [Varicose veins](http://r4.finngen.fi/pheno/I9_VARICVE) | -0.19 | 2.10E-02 | 13928 / 153951 |
|  |  |  |  |  |  |  |  |  |  | [Vascular dementia](http://r4.finngen.fi/pheno/I9_VASCDEM) | 0.7 | 2.50E-02 | 706 / 171075 |
|  |  |  |  |  |  |  |  |  |  | [Sequelae of cerebrovascular disease](http://r4.finngen.fi/pheno/I9_SEQULAE) | 0.31 | 3.00E-02 | 3520 / 165040 |
|  |  |  |  |  |  |  |  |  |  | [Aortic aneurysm](http://r4.finngen.fi/pheno/I9_AORTANEUR) | -0.41 | 3.40E-02 | 1919 / 167843 |
|  |  |  |  |  |  |  |  |  |  | [Secondary right heart disease](http://r4.finngen.fi/pheno/I9_SECONDRIGHT) | 0.94 | 3.60E-02 | 315 / 173597 |
|  |  |  |  |  |  |  |  |  |  | [Diseases of veins, lymphatic vessels and lymph nodes, not elsewhere classified](http://r4.finngen.fi/pheno/I9_DISVEINLYMPH) | -0.13 | 4.80E-02 | 22948 / 153951 |
|  | 7:66994286:T:A | K/* | rs120074160 | 0.001 | 0.603 | 0.55 | - | - |  |  |  |  |  |
| LIPC: APOA1 | 15:58541794:G:A | V/M | rs6078 | 0.032 | 0.171 | 0.20 | 1 | 0.01 | 1.79 | [Venous thromboembolism](http://r4.finngen.fi/pheno/I9_VTE) | -0.11 | 4.00E-03 | 6913 / 169986 |
|  |  |  |  |  |  |  |  |  |  | [DVT of lower extremities and pulmonary embolism](http://r4.finngen.fi/pheno/I9_DVTANDPULM) | -0.12 | 5.50E-03 | 6019 / 170880 |
|  |  |  |  |  |  |  |  |  |  | [Pulmonary heart disease, diseases of pulmonary circulation](http://r4.finngen.fi/pheno/I9_PULMHEART) | -0.13 | 1.90E-02 | 3302 / 173597 |
|  |  |  |  |  |  |  |  |  |  | [Aneurysms, operations, SAH](http://r4.finngen.fi/pheno/I9_SAHANEUR) | -0.18 | 2.40E-02 | 1582 / 165040 |
|  |  |  |  |  |  |  |  |  |  | [Other diseases of arteries and capillaries](http://r4.finngen.fi/pheno/I9_ARTOTH) | 0.21 | 2.90E-02 | 1046 / 167843 |
|  |  |  |  |  |  |  |  |  |  | [Cardiomyopathy, other and unspecified](http://r4.finngen.fi/pheno/I9_CARDMYOOTH) | -0.22 | 3.00E-02 | 971 / 129908 |
|  |  |  |  |  |  |  |  |  |  | [Pulmonary embolism](http://r4.finngen.fi/pheno/I9_PULMEMB) | -0.12 | 3.50E-02 | 3016 / 173597 |
|  |  |  |  |  |  |  |  |  |  | [Cerebral aneurysm, nonruptured](http://r4.finngen.fi/pheno/I9_ANEURYSM) | -0.25 | 3.70E-02 | 720 / 165040 |
|  |  |  |  |  |  |  |  |  |  | [Nontraumatic intracranial haemmorrhage](http://r4.finngen.fi/pheno/I9_INTRACRA) | -0.15 | 3.80E-02 | 2086 / 165040 |
|  |  |  |  |  |  |  |  |  |  | [Other peripheral vascular diseases](http://r4.finngen.fi/pheno/I9_OTHPER) | -0.23 | 4.30E-02 | 799 / 167843 |
|  | 15:58541843:C:T | P/L | rs562988299 | 0.001 | 0.877 | 0.38 | 0.31 | 0 |  |  |  |  |  |
|  | 15:58541944:G:A | A/T | rs201563586 | 0.001 | 1.794 | 0.07 | 0.01 | 0.97 | None | [Non-rheumatic valve diseases](http://r4.finngen.fi/pheno/I9_NONRHEVALV) | -0.48 | 2.10E-02 | 7440 / 129908 |
|  |  |  |  |  |  |  |  |  |  | [Other other unspecified disorders of the circulatory system](http://r4.finngen.fi/pheno/I9_OTHOTH) | 1.9 | 3.00E-02 | 340 / 175178 |
|  |  |  |  |  |  |  |  |  |  | [Aortic aneurysm](http://r4.finngen.fi/pheno/I9_AORTANEUR) | -0.78 | 4.20E-02 | 1919 / 167843 |
|  | 15:58548387:C:T | S/F | rs121912502 | 0.001 | 0.937 | 0.35 | 0 | 0.987 | 0.20 | [Intracerebral haemmorrhage](http://r4.finngen.fi/pheno/I9_ICH) | 2.6 | 7.80E-03 | 1224 / 163533 |
|  |  |  |  |  |  |  |  |  |  | [Transient ischemic attack](http://r4.finngen.fi/pheno/I9_TIA) | 0.96 | 9.20E-03 | 6729 / 164286 |
|  |  |  |  |  |  |  |  |  |  | [Other intracranial haemorrhages](http://r4.finngen.fi/pheno/I9_OTHINTRACRA) | 6.1 | 1.20E-02 | 230 / 165040 |
|  |  |  |  |  |  |  |  |  |  | [Cardiomyopathy, other and unspecified](http://r4.finngen.fi/pheno/I9_CARDMYOOTH) | 2.2 | 2.10E-02 | 971 / 129908 |
|  |  |  |  |  |  |  |  |  |  | [Nontraumatic intracranial haemmorrhage](http://r4.finngen.fi/pheno/I9_INTRACRA) | 1.3 | 3.10E-02 | 2086 / 165040 |
|  | 15:58560880:A:C | F/L | rs3829462 | 0.045 | 0.001 | 0.99 | - | - | 0.96 | Other or ill-defined heart diseases | 0.33 | 1.3e-2 | 583 / 129908 |
|  |  |  |  |  |  |  |  |  |  | Other pulmonary heart/vessel disease | -0.37 | 2.9e-2 | 363 / 173597 |
|  |  |  |  |  |  |  |  |  |  | Secondary right heart disease | -0.39 | 3.5e-2 | 315 / 173597 |
|  |  |  |  |  |  |  |  |  |  | Phlebitis and thrombophlebitis (not including DVT) | -0.14 | 4.2e-2 | 2506 / 153951 |
|  | 15:58563549:C:T | T/M | rs113298164 | 0.017 | 0.980 | 7.77E-08 | 0.02 | 0.996 | 4.41 | [Secondary hypertension](http://r4.finngen.fi/pheno/I9_HYPTENSEC) | 0.36 | 4.90E-02 | 982 / 133323 |
| GTF3C5: Non-HDLC | 9:133042147:C:T | H/Y | - | 0.001 | 0.596 | 0.55 | 0 | 0.998 |  |  |  |  |  |
|  | 9:133043731:T:C | Start lost; M/T | rs189383196 | 0.004 | 0.311 | 0.38 | 0 | 0.932 | 84.2 | [Non-ischemic cardiomyopathy](http://r4.finngen.fi/pheno/I9_NONISCHCARDMYOP) | 0.33 | 2.80E-05 | 8300 / 143233 |
|  |  |  |  |  |  |  |  |  |  | [Hypertension](http://r4.finngen.fi/pheno/I9_HYPTENS) | 0.16 | 6.70E-04 | 43545 / 133323 |
|  |  |  |  |  |  |  |  |  |  | [Hypertensive diseases](http://r4.finngen.fi/pheno/I9_HYPERTENSION) | 0.15 | 7.50E-04 | 43576 / 133323 |
|  |  |  |  |  |  |  |  |  |  | [Cardiovascular diseases](http://r4.finngen.fi/pheno/I9_CVD) | 0.12 | 1.90E-03 | 86957 / 89942 |
|  |  |  |  |  |  |  |  |  |  | [Statin medication](http://r4.finngen.fi/pheno/RX_STATIN) | 0.14 | 2.10E-03 | 53518 / 123381 |
|  |  |  |  |  |  |  |  |  |  | [DVT of lower extremities](http://r4.finngen.fi/pheno/I9_PHLETHROMBDVTLOW) | 0.35 | 2.20E-03 | 3592 / 153951 |
|  |  |  |  |  |  |  |  |  |  | [All-cause Heart Failure](http://r4.finngen.fi/pheno/I9_HEARTFAIL_ALLCAUSE) | 0.17 | 4.50E-03 | 17387 / 159058 |
|  |  |  |  |  |  |  |  |  |  | [Secondary right heart disease](http://r4.finngen.fi/pheno/I9_SECONDRIGHT) | 1 | 4.80E-03 | 315 / 173597 |
|  |  |  |  |  |  |  |  |  |  | [Venous thromboembolism](http://r4.finngen.fi/pheno/I9_VTE) | 0.24 | 5.30E-03 | 6913 / 169986 |
|  |  |  |  |  |  |  |  |  |  | [Heart failure, not strict](http://r4.finngen.fi/pheno/I9_HEARTFAIL_NS) | 0.16 | 6.70E-03 | 17613 / 159286 |
|  |  |  |  |  |  |  |  |  |  | [Hypertension, essential](http://r4.finngen.fi/pheno/I9_HYPTENSESS) | 0.13 | 7.20E-03 | 33229 / 133323 |
|  |  |  |  |  |  |  |  |  |  | [Phlebitis and thrombophlebitis (not including DVT)](http://r4.finngen.fi/pheno/I9_PHLETHROM) | 0.37 | 7.90E-03 | 2506 / 153951 |
|  |  |  |  |  |  |  |  |  |  | [Other heart diseases](http://r4.finngen.fi/pheno/I9_OTHHEART) | 0.1 | 1.00E-02 | 46991 / 129908 |
|  |  |  |  |  |  |  |  |  |  | [Peripheral artery operations in Hilmo](http://r4.finngen.fi/pheno/I9_PERIPH) | 0.45 | 1.70E-02 | 1254 / 167843 |
|  |  |  |  |  |  |  |  |  |  | [Conduction disorders](http://r4.finngen.fi/pheno/I9_CONDUCTIO) | 0.29 | 1.80E-02 | 3271 / 129908 |
|  |  |  |  |  |  |  |  |  |  | [Other pulmonary heart/vessel disease](http://r4.finngen.fi/pheno/I9_PULMOTHHD) | 0.79 | 1.90E-02 | 363 / 173597 |
|  |  |  |  |  |  |  |  |  |  | [Diseases of arteries, arterioles and capillaries (FINNGEN)](http://r4.finngen.fi/pheno/FG_DOAAC) | 0.22 | 2.80E-02 | 6988 / 97214 |
|  |  |  |  |  |  |  |  |  |  | [Diseases of veins, lymphatic vessels and lymph nodes, not elsewhere classified](http://r4.finngen.fi/pheno/I9_DISVEINLYMPH) | 0.11 | 2.90E-02 | 22948 / 153951 |
|  |  |  |  |  |  |  |  |  |  | [Atrial fibrillation and flutter](http://r4.finngen.fi/pheno/I9_AF) | 0.17 | 3.00E-02 | 17325 / 97214 |
|  |  |  |  |  |  |  |  |  |  | [Diseases of arteries, arterioles and capillaries](http://r4.finngen.fi/pheno/I9_DOAAC) | 0.15 | 4.70E-02 | 9056 / 167843 |
|  | 9:133043838:C:A | Q/K | rs150056568 | 0.001 | 0.017 | 0.98 | 0.08 | 0.755 | 2.30 | [Non-ischemic cardiomyopathy](http://r4.finngen.fi/pheno/I9_NONISCHCARDMYOP) | 0.46 | 7.80E-03 | 8300 / 143233 |
|  |  |  |  |  |  |  |  |  |  | [Nonischemic cardiomyopathy](http://r4.finngen.fi/pheno/I9_NONISCHCARDMYOP_STRICT) | 1.5 | 1.20E-02 | 653 / 150661 |
|  |  |  |  |  |  |  |  |  |  | [Other diseases of arteries and capillaries](http://r4.finngen.fi/pheno/I9_ARTOTH) | -1 | 2.40E-02 | 1046 / 167843 |
|  |  |  |  |  |  |  |  |  |  | [Transient ischemic attack](http://r4.finngen.fi/pheno/I9_TIA) | -0.42 | 2.60E-02 | 6729 / 164286 |
|  |  |  |  |  |  |  |  |  |  | [Nonspesific lymphadenitis](http://r4.finngen.fi/pheno/I9_LYMPHADE) | 1.1 | 2.60E-02 | 806 / 153951 |
|  |  |  |  |  |  |  |  |  |  | [Ischaemic Stroke, excluding all haemorrhages](http://r4.finngen.fi/pheno/I9_STR_EXH) | -0.35 | 4.70E-02 | 8046 / 164286 |
|  |  |  |  |  |  |  |  |  |  | [Cardiomyopathy](http://r4.finngen.fi/pheno/I9_CARDMYO) | 0.61 | 4.80E-02 | 2342 / 129908 |
|  | 9:133054459:A:G | Y/C | rs369889499 | 0.001 | -0.392 | 0.58 | 0 | 0.993 | None | [Angina pectoris](http://r4.finngen.fi/pheno/I9_ANGINA) | 1 | 9.20E-05 | 14712 / 152621 |
|  |  |  |  |  |  |  |  |  |  | [Ischaemic heart disease, wide definition](http://r4.finngen.fi/pheno/I9_IHD) | 0.71 | 6.10E-04 | 25366 / 151533 |
|  |  |  |  |  |  |  |  |  |  | [Unstable angina pectoris](http://r4.finngen.fi/pheno/I9_UAP) | 1.2 | 2.40E-03 | 5774 / 160163 |
|  |  |  |  |  |  |  |  |  |  | [Ischemic heart diseases](http://r4.finngen.fi/pheno/I9_ISCHHEART) | 0.62 | 3.10E-03 | 24278 / 152621 |
|  |  |  |  |  |  |  |  |  |  | [Cardiomyopathies, Primary/intrinsic](http://r4.finngen.fi/pheno/I9_CARDMPRI) | 1.7 | 3.40E-03 | 1678 / 129908 |
|  |  |  |  |  |  |  |  |  |  | [Other diseases of pericardium](http://r4.finngen.fi/pheno/I9_PERICAOTH) | 5.1 | 4.50E-03 | 307 / 129908 |
|  |  |  |  |  |  |  |  |  |  | [Cardiomyopathy, Hypertrophic obstructive](http://r4.finngen.fi/pheno/I9_CARDMYOHYP) | 4.7 | 7.00E-03 | 251 / 129908 |
|  |  |  |  |  |  |  |  |  |  | [Coronary revascularization (ANGIO or CABG)](http://r4.finngen.fi/pheno/I9_REVASC) | 0.79 | 1.20E-02 | 9971 / 152621 |
|  |  |  |  |  |  |  |  |  |  | [Myocarditis](http://r4.finngen.fi/pheno/I9_MYOCARD) | 2.7 | 1.30E-02 | 626 / 97214 |
|  |  |  |  |  |  |  |  |  |  | [Other CVD (FINNGEN)](http://r4.finngen.fi/pheno/FG_OTH) | 2 | 1.40E-02 | 1398 / 97214 |
|  |  |  |  |  |  |  |  |  |  | [Other disorders of veins](http://r4.finngen.fi/pheno/I9_VEINSOTH) | -0.88 | 2.10E-02 | 4588 / 153951 |
|  |  |  |  |  |  |  |  |  |  | [Valvular operations](http://r4.finngen.fi/pheno/I9_VALVES) | 0.39 | 2.30E-02 | 26015 / 129908 |
|  |  |  |  |  |  |  |  |  |  | [Valvular heart disease including rheumatic fever](http://r4.finngen.fi/pheno/I9_VHD) | 0.38 | 2.30E-02 | 27684 / 129908 |
|  |  |  |  |  |  |  |  |  |  | [Cardiomyopathy](http://r4.finngen.fi/pheno/I9_CARDMYO) | 1 | 3.00E-02 | 2342 / 129908 |
|  |  |  |  |  |  |  |  |  |  | [Left bundle-branch block](http://r4.finngen.fi/pheno/I9_LBBB) | 2.4 | 3.20E-02 | 582 / 129908 |
|  |  |  |  |  |  |  |  |  |  | [Cardiovascular diseases](http://r4.finngen.fi/pheno/I9_CVD) | 0.29 | 3.60E-02 | 86957 / 89942 |
|  |  |  |  |  |  |  |  |  |  | [Coronary atherosclerosis](http://r4.finngen.fi/pheno/I9_CORATHER) | 0.51 | 3.70E-02 | 18295 / 152621 |
|  |  |  |  |  |  |  |  |  |  | [Hypertrophic cardiomyopathy](http://r4.finngen.fi/pheno/I9_HYPERTROCARDMYOP) | 2.2 | 3.80E-02 | 432 / 176467 |
|  |  |  |  |  |  |  |  |  |  | [Endocarditis](http://r4.finngen.fi/pheno/I9_ENDOCARD) | 2.7 | 4.70E-02 | 312 / 129908 |
|  |  |  |  |  |  |  |  |  |  | [Heart failure,strict](http://r4.finngen.fi/pheno/I9_HEARTFAIL) | 0.54 | 4.80E-02 | 9576 / 159286 |
|  |  |  |  |  |  |  |  |  |  | [Hypertension](http://r4.finngen.fi/pheno/I9_HYPTENS) | 0.33 | 4.90E-02 | 43545 / 133323 |
|  | 9:133054733:A:G | H/R | rs1302997790 | 0.001 | 0.840 | 0.40 | 0.5 | 0.011 |  |  |  |  |  |
|  | 9:133054787:C:T | A/V | rs202207045 | 0.007 | -1.382 | 7.26E-07 | 0.24 | 0.03 | None | [Vascular dementia](http://r4.finngen.fi/pheno/I9_VASCDEM) | -0.76 | 2.10E-02 | 706 / 171075 |
|  | 9:133056848:G:A | D/N | rs637435 | 0.001 | -0.596 | 0.55 | 0.04 | 0.833 |  |  |  |  |  |
|  | 9:133057819:T:C | F/L | rs111893665 | 0.001 | -0.120 | 0.90 | 0.27 | 0.914 | 0.152* | [Other peripheral vascular diseases](http://r4.finngen.fi/pheno/I9_OTHPER) | 2.9 | 1.00E-02 | 799 / 167843 |
|  |  |  |  |  |  |  |  |  |  | [Paroxysmal tachycardia](http://r4.finngen.fi/pheno/I9_PAROXTAC) | -1.1 | 2.10E-02 | 3667 / 97214 |
| *TRMT5:* VLDLPL_XS | 14:60975180:G:A | P/S | rs147405788 | 0.001 | -1.575 | 0.03 | 0 | 0.88 | 6.24 | [Type 2 diabetes with peripheral circulatory complications](http://r4.finngen.fi/pheno/E4_DM2PERIPH) | -1.1 | 3.40E-02 | 820 / 148190 |
|  |  |  |  |  |  |  |  |  |  | [Cardiomyopathy, Hypertrophic obstructive](http://r4.finngen.fi/pheno/I9_CARDMYOHYP) | 1.7 | 3.60E-02 | 251 / 129908 |
|  | 14:60975182:T:C | D/G | - | 0.001 | -1.386 | 0.17 | 0.1 | 0.001 |  |  |  |  |  |
|  | 14:60975636:G:A | A/V | rs45604437 | 0.007 | -1.094 | 5.76E-04 | 0.16 | 0 | 2.73 | [Other peripheral vascular diseases](http://r4.finngen.fi/pheno/I9_OTHPER) | 1.1 | 4.90E-03 | 799 / 167843 |
|  |  |  |  |  |  |  |  |  |  | [Phlebitis and thrombophlebitis (not including DVT)](http://r4.finngen.fi/pheno/I9_PHLETHROM) | 0.5 | 1.90E-02 | 2506 / 153951 |
|  |  |  |  |  |  |  |  |  |  | [Sequelae of cerebrovascular disease](http://r4.finngen.fi/pheno/I9_SEQULAE) | -0.44 | 2.10E-02 | 3520 / 165040 |
|  |  |  |  |  |  |  |  |  |  | [Hypertensive Renal Disease](http://r4.finngen.fi/pheno/I9_HYPTENSRD) | 1.2 | 2.60E-02 | 373 / 133323 |
|  |  |  |  |  |  |  |  |  |  | [Stroke, excluding SAH](http://r4.finngen.fi/pheno/I9_STR) | -0.25 | 4.60E-02 | 8877 / 163535 |
|  |  |  |  |  |  |  |  |  |  | [Stroke, including SAH](http://r4.finngen.fi/pheno/I9_STR_SAH) | -0.23 | 4.90E-02 | 9592 / 162861 |
|  | 14:60979242:T:C | K/R | rs138139551 | 0.002 | -0.599 | 0.30 | 0.2 | 0.253 | None | [Other noninfective disordersof lymphatic vessels and lymph nodes](http://r4.finngen.fi/pheno/I9_LYMPHOTH) | 2.8 | 3.00E-03 | 301 / 153951 |
|  |  |  |  |  |  |  |  |  |  | [Left bundle-branch block](http://r4.finngen.fi/pheno/I9_LBBB) | 1.6 | 1.30E-02 | 582 / 129908 |
|  |  |  |  |  |  |  |  |  |  | [Occlusion and stenosis of arteries, not leading to stroke](http://r4.finngen.fi/pheno/I9_STENOSIS) | 2.7 | 2.50E-02 | 147 / 165040 |
|  |  |  |  |  |  |  |  |  |  | [DVT of lower extremities](http://r4.finngen.fi/pheno/I9_PHLETHROMBDVTLOW) | -0.58 | 2.70E-02 | 3592 / 153951 |
|  |  |  |  |  |  |  |  |  |  | [Status post-ami](http://r4.finngen.fi/pheno/I9_POSTAMI) | 0.96 | 2.70E-02 | 1141 / 152621 |
|  |  |  |  |  |  |  |  |  |  | [Hypertensive Renal Disease](http://r4.finngen.fi/pheno/I9_HYPTENSRD) | 1.8 | 3.40E-02 | 373 / 133323 |
|  |  |  |  |  |  |  |  |  |  | [Conduction disorders](http://r4.finngen.fi/pheno/I9_CONDUCTIO) | 0.57 | 3.70E-02 | 3271 / 129908 |
|  | 14:60979257:T:C | H/R | rs191207997 | 0.001 | -0.713 | 0.48 | 0.05 | 0.27 | 3.12 | [Hypertensive Heart Disease](http://r4.finngen.fi/pheno/I9_HYPTENSHD) | 0.95 | 1.40E-02 | 3252 / 133323 |
|  |  |  |  |  |  |  |  |  |  | [Non-rheumatic valve diseases](http://r4.finngen.fi/pheno/I9_NONRHEVALV) | 0.62 | 1.50E-02 | 7440 / 129908 |
|  | 14:60979303:C:G | G/R | rs1285433520 | 0.001 | 0.400 | 0.69 | 0 | 0.588 |  |  |  |  |  |
|  | 14:60979401:G:A | P/L | rs746471581 | 0.001 | 1.362 | 0.17 | 0.02 | 0.031 |  |  |  |  |  |
|  | 14:60979429:T:A | S/C | rs115400838 | 0.007 | -1.211 | 1.59E-04 | 0.03 | 0.375 | 6.99 | [Stroke, excluding SAH](http://r4.finngen.fi/pheno/I9_STR) | 0.24 | 1.90E-04 | 8877 / 163535 |
|  |  |  |  |  |  |  |  |  |  | [Stroke, including SAH](http://r4.finngen.fi/pheno/I9_STR_SAH) | 0.22 | 3.70E-04 | 9592 / 162861 |
|  |  |  |  |  |  |  |  |  |  | [Sequelae of cerebrovascular disease](http://r4.finngen.fi/pheno/I9_SEQULAE) | 0.35 | 5.40E-04 | 3520 / 165040 |
|  |  |  |  |  |  |  |  |  |  | [Ischaemic Stroke, excluding all haemorrhages](http://r4.finngen.fi/pheno/I9_STR_EXH) | 0.23 | 6.00E-04 | 8046 / 164286 |
|  |  |  |  |  |  |  |  |  |  | [Cerebrovascular diseases](http://r4.finngen.fi/pheno/I9_CEREBVASC) | 0.18 | 1.60E-03 | 11859 / 165040 |
|  |  |  |  |  |  |  |  |  |  | [Cerebrovascular diseases (FINNGEN)](http://r4.finngen.fi/pheno/FG_CEREBVASC) | 0.17 | 1.40E-02 | 10367 / 97214 |
|  |  |  |  |  |  |  |  |  |  | [Arterial embolism and thrombosis](http://r4.finngen.fi/pheno/I9_ARTEMBTHR) | 0.58 | 1.50E-02 | 580 / 167843 |
|  |  |  |  |  |  |  |  |  |  | [Rheumatic fever incl heart disease](http://r4.finngen.fi/pheno/I9_RHEUFEV) | 0.56 | 3.30E-02 | 462 / 176437 |
|  |  |  |  |  |  |  |  |  |  | [Valvular operations](http://r4.finngen.fi/pheno/I9_VALVES) | 0.087 | 3.50E-02 | 26015 / 129908 |
| RBM47: APOC3 | 4:40426074:C:T | G/R | rs35529250 | 0.001 | -1.020 | 0.31 | 0 | 0.905 | 0.81 | [Cardiomyopathy, Hypertrophic obstructive](http://r4.finngen.fi/pheno/I9_CARDMYOHYP) | 2.3 | 2.10E-03 | 251 / 129908 |
|  |  |  |  |  |  |  |  |  |  | [Hypertrophic cardiomyopathy](http://r4.finngen.fi/pheno/I9_HYPERTROCARDMYOP) | 1.4 | 7.30E-03 | 432 / 176467 |
|  |  |  |  |  |  |  |  |  |  | [Hypertension, essential](http://r4.finngen.fi/pheno/I9_HYPTENSESS) | -0.18 | 1.60E-02 | 33229 / 133323 |
|  |  |  |  |  |  |  |  |  |  | [Hypertensive diseases](http://r4.finngen.fi/pheno/I9_HYPERTENSION) | -0.17 | 1.90E-02 | 43576 / 133323 |
|  |  |  |  |  |  |  |  |  |  | [Hypertension](http://r4.finngen.fi/pheno/I9_HYPTENS) | -0.17 | 1.90E-02 | 43545 / 133323 |
|  |  |  |  |  |  |  |  |  |  | [Phlebitis and thrombophlebitis (not including DVT)](http://r4.finngen.fi/pheno/I9_PHLETHROM) | -0.45 | 2.30E-02 | 2506 / 153951 |
|  |  |  |  |  |  |  |  |  |  | [Diseases of veins, lymphatic vessels and lymph nodes, not elsewhere classified](http://r4.finngen.fi/pheno/I9_DISVEINLYMPH) | -0.17 | 3.50E-02 | 22948 / 153951 |
|  |  |  |  |  |  |  |  |  |  | [Stroke, excluding SAH](http://r4.finngen.fi/pheno/I9_STR) | -0.24 | 3.70E-02 | 8877 / 163535 |
|  |  |  |  |  |  |  |  |  |  | [Ischaemic Stroke, excluding all haemorrhages](http://r4.finngen.fi/pheno/I9_STR_EXH) | -0.24 | 4.30E-02 | 8046 / 164286 |
|  | 4:40432687:AGCGGCTGCGGCGGCTGCGGCC:A | AAAAAAAA/A | rs564837143 | 0.005 | -1.938 | 2.50E-06 | - | - |  | [Secondary hypertension](http://r4.finngen.fi/pheno/I9_HYPTENSEC) | -0.65 | 1.10E-02 | 982 / 133323 |
|  |  |  |  |  |  |  |  |  |  | [Other peripheral vascular diseases](http://r4.finngen.fi/pheno/I9_OTHPER) | -0.58 | 3.50E-02 | 799 / 167843 |
|  | 4:40438508:C:T | R/H | rs373211767 | 0.002 | 0.817 | 0.25 | 0.03 | 0.891 | None | [Vascular syndromes of brain in cerebrovascular disorders](http://r4.finngen.fi/pheno/I9_VASCSYND) | 3.4 | 6.90E-03 | 458 / 170687 |
|  |  |  |  |  |  |  |  |  |  | [Angina pectoris](http://r4.finngen.fi/pheno/I9_ANGINA) | 0.58 | 1.20E-02 | 14712 / 152621 |
|  |  |  |  |  |  |  |  |  |  | [Aortic aneurysm](http://r4.finngen.fi/pheno/I9_AORTANEUR) | 1.1 | 2.80E-02 | 1919 / 167843 |
|  |  |  |  |  |  |  |  |  |  | [Status post-ami](http://r4.finngen.fi/pheno/I9_POSTAMI) | 1.4 | 2.80E-02 | 1141 / 152621 |
|  |  |  |  |  |  |  |  |  |  | [Other specified cerebrovascular diseases, other cerebrovascular disorders in diseases classified elsewhere](http://r4.finngen.fi/pheno/I9_CEREBDOTH) | 1.9 | 3.10E-02 | 729 / 165040 |
|  |  |  |  |  |  |  |  |  |  | [Other aneurysm](http://r4.finngen.fi/pheno/I9_OTHANEUR) | 2.1 | 4.00E-02 | 444 / 167843 |
| MARCHF10: VLDLPL XS | 17:62736189:G:A | T/I | rs147046907 | 0.003 | -2.186 | 1.07E-06 | 0.05 | 0.031 | 0.602 | Thoracic aortic aneurysm | 0.39 | 5.50E-03 | 5881 / 288638 |
|  |  |  |  |  |  |  |  |  |  | Aortic aneurysm | 0.37 | 7.40E-03 | 6092 / 288638 |
|  |  |  |  |  |  |  |  |  |  | Left bundle-branch block | 0.66 | 8.80E-03 | 1373 / 218984 |
|  |  |  |  |  |  |  |  |  |  | Conduction disorders | 0.32 | 1.20E-02 | 7262 / 218984 |
|  |  |  |  |  |  |  |  |  |  | Alcoholic cardiomyopathy | 2.70 | 1.50E-02 | 99 / 304548 |
|  |  |  |  |  |  |  |  |  |  | Coronary revascularization (ANGIO or CABG) | -0.22 | 2.80E-02 | 19151 / 260124 |
|  |  |  |  |  |  |  |  |  |  | Coronary artery bypass grafting | -0.30 | 3.30E-02 | 8693 / 260124 |
|  |  |  |  |  |  |  |  |  |  | Paroxysmal tachycardia | -0.29 | 3.40E-02 | 7401 / 156457 |
|  |  |  |  |  |  |  |  |  |  | Embolic stroke with flimmer | 1.10 | 3.50E-02 | 496 / 274406 |
|  |  |  |  |  |  |  |  |  |  | Other specified cerebrovascular diseases, other cerebrovascular disorders in diseases classified elsewhere | 0.61 | 4.00E-02 | 1487 / 284164 |
|  | 17:62736691:T:G | S/R | rs140716269 | 0.005 | 0.229 | 0.55 | 0.16 | 0.034 | 78 | Varicose veins of other sites | 0.58 | 6.50E-03 | 1469 / 267090 |
|  |  |  |  |  |  |  |  |  |  | Coronary angiopasty | 0.24 | 7.10E-03 | 12553 / 260124 |
|  |  |  |  |  |  |  |  |  |  | Complications following myocardial infarction | 1.90 | 1.20E-02 | 173 / 260124 |
|  |  |  |  |  |  |  |  |  |  | Rheumatic fever incl heart disease | 0.82 | 1.50E-02 | 808 / 308346 |
|  |  |  |  |  |  |  |  |  |  | Diseases of veins, lymphatic vessels and lymph nodes, not elsewhere classified | 0.11 | 4.10E-02 | 42064 / 267090 |
|  |  |  |  |  |  |  |  |  |  | Rheumatic valve diseases | 0.84 | 4.20E-02 | 575 / 308346 |
|  |  |  |  |  |  |  |  |  |  | Other other unspecified disorders of the circulatory system | 0.74 | 4.30E-02 | 771 / 305353 |
|  |  |  |  |  |  |  |  |  |  | Cardiac arrest | 0.49 | 4.60E-02 | 1761 / 156457 |
|  |  |  |  |  |  |  |  |  |  | Coronary revascularization (ANGIO or CABG) | 0.16 | 4.70E-02 | 19151 / 260124 |
|  |  |  |  |  |  |  |  |  |  | Atherosclerosis, excluding cerebral, coronary and PAD | 0.19 | 4.80E-02 | 11189 / 288638 |
|  | 17:62737066:A:G | S/P | rs1329816461 | 0.0007 | 0.368 | 0.71 | 1 | 0 | NA |  |  |  |  |
|  | 17:62746934:C:T | G/R | rs916315847 | 0.0007 | -2.213 | 0.03 | 0.02 | 0.001 | NA |  |  |  |  |
|  | 17:62801665:T:C | K/R | rs199705946 | 0.003 | -0.919 | 0.07 | 0.02 | 0.979 | None | Cardiomyopathy, Hypertrophic obstructive | 1.30 | 3.40E-05 | 433 / 218984 |
|  |  |  |  |  |  |  |  |  |  | Hypertrophic cardiomyopathy | 1.00 | 2.00E-04 | 808 / 308346 |
|  |  |  |  |  |  |  |  |  |  | Dissection of cerebral arteries, nonruptured | 1.50 | 1.00E-02 | 137 / 284164 |
|  |  |  |  |  |  |  |  |  |  | Major coronary heart disease event | 0.19 | 1.30E-02 | 33628 / 275526 |
|  |  |  |  |  |  |  |  |  |  | Varicose veins | -0.18 | 3.00E-02 | 24281 / 267090 |
|  |  |  |  |  |  |  |  |  |  | Diseases of veins, lymphatic vessels and lymph nodes, not elsewhere classified | -0.14 | 3.50E-02 | 42064 / 267090 |
|  |  |  |  |  |  |  |  |  |  | Cardiovascular diseases | -0.10 | 3.90E-02 | 166137 / 143017 |
|  |  |  |  |  |  |  |  |  |  | Dissection of aorta | 0.90 | 4.00E-02 | 680 / 288638 |
|  |  |  |  |  |  |  |  |  |  | Dronedarone medication | 0.94 | 4.40E-02 | 638 / 218984 |
|  |  |  |  |  |  |  |  |  |  | Coronary atherosclerosis | 0.15 | 4.80E-02 | 36418 / 260124 |
|  |  |  |  |  |  |  |  |  |  | Peripheral artery operations in Hilmo | -0.43 | 4.80E-02 | 2971 / 288638 |
| *RYR3*: VLDLTG XS | 15:33579984:A:G | N/S | rs146838868 | 0.008 | 0.318 | 0.28 | 0.23 | 0.097 | 221 | Valvular operations | -0.09 | 9.70E-04 | 59338 / 218984 |
|  |  |  |  |  |  |  |  |  |  | Other heart diseases | -0.08 | 1.00E-03 | 90170 / 218984 |
|  |  |  |  |  |  |  |  |  |  | Valvular heart disease excluding rheumatic fever | -0.08 | 2.20E-03 | 62218 / 218984 |
|  |  |  |  |  |  |  |  |  |  | Atrial fibrillation and flutter with reimbursement | -0.16 | 3.30E-03 | 17629 / 156457 |
|  |  |  |  |  |  |  |  |  |  | Atrial fibrillation and flutter | -0.13 | 3.40E-03 | 34748 / 156457 |
|  |  |  |  |  |  |  |  |  |  | Ischemic heart diseases | -0.09 | 5.40E-03 | 49030 / 260124 |
|  |  |  |  |  |  |  |  |  |  | Ischaemic heart disease, wide definition | -0.09 | 6.30E-03 | 48706 / 260448 |
|  |  |  |  |  |  |  |  |  |  | Coronary atherosclerosis | -0.09 | 9.60E-03 | 36418 / 260124 |
|  |  |  |  |  |  |  |  |  |  | Calcific aortic valvular stenosis, including rheumatic fever | -0.17 | 1.10E-02 | 6907 / 302247 |
|  |  |  |  |  |  |  |  |  |  | Hypertensive heart and/or renal disease | -0.17 | 1.10E-02 | 7222 / 223663 |
|  |  |  |  |  |  |  |  |  |  | Coronary artery bypass grafting | -0.17 | 1.10E-02 | 8693 / 260124 |
|  |  |  |  |  |  |  |  |  |  | Calcific aortic valvular stenosis | -0.17 | 1.30E-02 | 6870 / 302284 |
|  |  |  |  |  |  |  |  |  |  | Calcific aortic valvular stenosis, operated | -0.17 | 1.30E-02 | 6870 / 302284 |
|  |  |  |  |  |  |  |  |  |  | Hypertensive Heart Disease | -0.18 | 1.50E-02 | 6348 / 223663 |
|  |  |  |  |  |  |  |  |  |  | Coronary revascularization (ANGIO or CABG) | -0.11 | 1.50E-02 | 19151 / 260124 |
|  |  |  |  |  |  |  |  |  |  | Hypertension | -0.06 | 1.70E-02 | 85438 / 223663 |
|  |  |  |  |  |  |  |  |  |  | Hypertensive diseases | -0.06 | 1.70E-02 | 85491 / 223663 |
|  |  |  |  |  |  |  |  |  |  | Cardiovascular diseases | -0.05 | 1.90E-02 | 166137 / 143017 |
|  |  |  |  |  |  |  |  |  |  | DVT of lower extremities | -0.16 | 1.90E-02 | 7008 / 267090 |
|  |  |  |  |  |  |  |  |  |  | Other peripheral vascular diseases | 0.31 | 2.10E-02 | 1658 / 288638 |
|  |  |  |  |  |  |  |  |  |  | DVT of lower extremities and pulmonary embolism | -0.12 | 2.20E-02 | 12559 / 296595 |
|  |  |  |  |  |  |  |  |  |  | Heart failure,strict | -0.10 | 2.30E-02 | 19676 / 272371 |
|  |  |  |  |  |  |  |  |  |  | Other disorders of veins | -0.13 | 2.70E-02 | 9288 / 267090 |
|  |  |  |  |  |  |  |  |  |  | Conduction disorders | -0.14 | 3.20E-02 | 7262 / 218984 |
|  |  |  |  |  |  |  |  |  |  | All-cause Heart Failure | -0.09 | 3.40E-02 | 19350 / 288996 |
|  |  |  |  |  |  |  |  |  |  | Venous thromboembolism | -0.10 | 3.70E-02 | 14454 / 294700 |
|  |  |  |  |  |  |  |  |  |  | Aortic valve malfunction, age under 60 years | 0.24 | 4.00E-02 | 2085 / 307069 |
|  |  |  |  |  |  |  |  |  |  | Heart failure, not strict | -0.07 | 4.20E-02 | 36783 / 272371 |
|  |  |  |  |  |  |  |  |  |  | Hypertension, essential | -0.05 | 4.80E-02 | 70651 / 223663 |
|  |  |  |  |  |  |  |  |  |  | Angina pectoris | -0.08 | 4.90E-02 | 27046 / 260124 |
|  | 15:33601461:G:A | A/T | rs979125271 | 0.0007 | 1.258 | 0.21 | 0 | 1 | NA |  |  |  |  |
|  | 15:33629953:A:G | N/S | rs2229119 | 0.007 | 0.812 | 7.42E-03 | 0.38 | 0.249 | 4.21 | Status post-ami | 0.58 | 2.30E-04 | 2429 / 260124 |
|  |  |  |  |  |  |  |  |  |  | Myocardial infarction | 0.21 | 1.90E-03 | 20404 / 260124 |
|  |  |  |  |  |  |  |  |  |  | Myocardial infarction, strict | 0.21 | 2.20E-03 | 18339 / 260124 |
|  |  |  |  |  |  |  |  |  |  | Other other unspecified disorders of the circulatory system | -1.40 | 3.40E-03 | 771 / 305353 |
|  |  |  |  |  |  |  |  |  |  | Death due to cardiac causes | 0.22 | 3.70E-03 | 13673 / 295481 |
|  |  |  |  |  |  |  |  |  |  | Hypertensive Renal Disease | 0.65 | 8.50E-03 | 777 / 223663 |
|  |  |  |  |  |  |  |  |  |  | Cardiomyopathy in other diseases | 2.20 | 1.30E-02 | 87 / 218984 |
|  |  |  |  |  |  |  |  |  |  | Major coronary heart disease event excluding revascularizations | 0.13 | 2.30E-02 | 27443 / 275228 |
|  |  |  |  |  |  |  |  |  |  | Hypotension | 0.34 | 2.30E-02 | 3061 / 305353 |
|  |  |  |  |  |  |  |  |  |  | Statin medication | 0.08 | 3.00E-02 | 100639 / 208515 |
|  |  |  |  |  |  |  |  |  |  | Hypertension, Pulmonary Arterial | 1.30 | 3.20E-02 | 179 / 223663 |
|  |  |  |  |  |  |  |  |  |  | Cardiovascular diseases | 0.07 | 4.20E-02 | 166137 / 143017 |
|  |  |  |  |  |  |  |  |  |  | Other CVD (FINNGEN) | 0.32 | 4.70E-02 | 3061 / 156457 |
|  |  |  |  |  |  |  |  |  |  | Paroxysmal tachycardia | 0.20 | 4.80E-02 | 7401 / 156457 |
|  |  |  |  |  |  |  |  |  |  | Heart failure, not strict | 0.10 | 4.80E-02 | 36783 / 272371 |
|  | 15:33634625:G:A | A/T | rs749982964 | 0.0007 | 0.612 | 0.54 | 0.56 | 0.009 | NA |  |  |  |  |
|  | 15:33649129:G:A | G/R | rs1309424472 | 0.0007 | 1.064 | 0.29 | 0 | 0.999 | NA |  |  |  |  |
|  | 15:33652758:A:G | I/V | rs571025757 | 0.0007 | 1.091 | 0.27 | 0.25 | 0.001 | NA |  |  |  |  |
|  | 15:33663642:A:C | K/Q | rs201524472 | 0.0007 | 0.674 | 0.50 | 0.1 | 0.509 | NA |  |  |  |  |
|  | 15:33699813:A:G | E/G | rs370232598 | 0.0007 | -0.359 | 0.72 | 0 | 0.748 | NA |  |  |  |  |
|  | 15:33706919:G:T | V/L | rs766431661 | 0.0007 | -0.146 | 0.88 | 0.01 | 0.212 | NA |  |  |  |  |
|  | 15:33728863:C:T | S/L | rs781065862 | 0.0007 | 1.952 | 0.05 | 0.31 | 0.011 | NA |  |  |  |  |
|  | 15:33731519:A:G | I/V | rs200294137 | 0.0007 | -2.065 | 0.04 | 0.04 | 0.015 | 1.39 | Non-ischemic cardiomyopathy | 0.70 | 3.90E-03 | 7047 / 253401 |
|  |  |  |  |  |  |  |  |  |  | Thromboangiitis obliterans | 2.40 | 6.40E-03 | 85 / 288638 |
|  |  |  |  |  |  |  |  |  |  | Other other unspecified disorders of the circulatory system | 1.40 | 8.50E-03 | 771 / 305353 |
|  |  |  |  |  |  |  |  |  |  | Left bundle-branch block | 1.00 | 1.60E-02 | 1373 / 218984 |
|  |  |  |  |  |  |  |  |  |  | Transient ischemic attack | -0.52 | 1.80E-02 | 13790 / 283057 |
|  |  |  |  |  |  |  |  |  |  | Cerebral atherosclerosis | 2.00 | 1.80E-02 | 221 / 308933 |
|  |  |  |  |  |  |  |  |  |  | Cerebral atherosclerosis | 2.00 | 1.90E-02 | 221 / 284164 |
|  |  |  |  |  |  |  |  |  |  | Other or ill-defined heart diseases | 1.70 | 2.20E-02 | 1145 / 218984 |
|  |  |  |  |  |  |  |  |  |  | Rheumatic fever without mention of heart involvement | 5.90 | 2.50E-02 | 80 / 309074 |
|  | 15:33739987:C:G | N/K | rs41279214 | 0.0013 | 0.414 | 0.56 | 0 | 0.116 | 0.51 | Major coronary heart disease event excluding revascularizations | 0.24 | 6.20E-03 | 27443 / 275228 |
|  |  |  |  |  |  |  |  |  |  | Nonpyogenic thrombosis of intracranial venous system | 1.50 | 1.10E-02 | 169 / 284164 |
|  |  |  |  |  |  |  |  |  |  | Myocardial infarction, strict | 0.27 | 1.30E-02 | 18339 / 260124 |
|  |  |  |  |  |  |  |  |  |  | Unstable angina pectoris | 0.32 | 2.20E-02 | 10368 / 275228 |
|  |  |  |  |  |  |  |  |  |  | Major coronary heart disease event | 0.19 | 2.30E-02 | 33628 / 275526 |
|  |  |  |  |  |  |  |  |  |  | Abdominal aortic aneurysm (AAA) | 0.63 | 2.30E-02 | 2434 / 288638 |
|  |  |  |  |  |  |  |  |  |  | Myocardial infarction | 0.24 | 2.40E-02 | 20404 / 260124 |
|  |  |  |  |  |  |  |  |  |  | Death due to cardiac causes | 0.26 | 2.90E-02 | 13673 / 295481 |
|  |  |  |  |  |  |  |  |  |  | Conduction disorders | -0.33 | 3.80E-02 | 7262 / 218984 |
|  |  |  |  |  |  |  |  |  |  | Status post-ami | 0.55 | 4.00E-02 | 2429 / 260124 |
|  |  |  |  |  |  |  |  |  |  | Angina pectoris | 0.18 | 4.70E-02 | 27046 / 260124 |
|  | 15:33748495:G:A | D/N | rs376553827 | 0.0007 | 0.784 | 0.43 | 0.02 | 0.994 | NA |  |  |  |  |
|  | 15:33750262:A:G | Q/R | rs1429954855 | 0.0007 | -0.361 | 0.72 | 0.01 | 0.634 | NA |  |  |  |  |
|  | 15:33772108:C:T | T/M | rs776121340 | 0.0007 | 1.592 | 0.11 | 0 | 0.993 | NA |  |  |  |  |
|  | 15:33773591:C:T | T/M | rs764112773 | 0.0007 | -1.091 | 0.27 | 0 | 0.998 | NA |  |  |  |  |
|  | 15:33780327:C:G | P/R | rs61996335 | 0.005 | 0.792 | 0.03 | 0.05 | 0.307 | 0.86 | Coronary artery bypass grafting | 0.31 | 8.60E-03 | 8693 / 260124 |
|  |  |  |  |  |  |  |  |  |  | Calcific aortic valvular stenosis, including rheumatic fever | 0.31 | 1.90E-02 | 6907 / 302247 |
|  |  |  |  |  |  |  |  |  |  | Other aneurysm | 0.74 | 2.20E-02 | 1085 / 288638 |
|  |  |  |  |  |  |  |  |  |  | Calcific aortic valvular stenosis | 0.30 | 2.30E-02 | 6870 / 302284 |
|  |  |  |  |  |  |  |  |  |  | Calcific aortic valvular stenosis, operated | 0.30 | 2.30E-02 | 6870 / 302284 |
|  |  |  |  |  |  |  |  |  |  | Varicose veins of other sites | 0.61 | 2.40E-02 | 1469 / 267090 |
|  |  |  |  |  |  |  |  |  |  | Coronary revascularization (ANGIO or CABG) | 0.20 | 2.80E-02 | 19151 / 260124 |
|  |  |  |  |  |  |  |  |  |  | Other other unspecified disorders of the circulatory system | 0.77 | 3.70E-02 | 771 / 305353 |
|  |  |  |  |  |  |  |  |  |  | Cardiac arrest | 0.54 | 4.20E-02 | 1761 / 156457 |
|  |  |  |  |  |  |  |  |  |  | Intracerebral haemmorrhage | -0.42 | 4.40E-02 | 2643 / 281373 |
|  | 15:33785748:G:A | E/K | rs200830195 | 0.005 | 0.092 | 0.80 | 0.01 | 0.342 | 1.9 | Atrial fibrillation and flutter with reimbursement | 0.31 | 1.70E-03 | 17629 / 156457 |
|  |  |  |  |  |  |  |  |  |  | Alcoholic cardiomyopathy | 1.50 | 1.00E-02 | 99 / 304548 |
|  |  |  |  |  |  |  |  |  |  | Cerebrovascular diseases (FINNGEN) | 0.21 | 2.00E-02 | 21738 / 156457 |
|  |  |  |  |  |  |  |  |  |  | Progressive vascular leukoencephalopathy | 1.10 | 2.50E-02 | 452 / 284164 |
|  |  |  |  |  |  |  |  |  |  | Cerebrovascular diseases | 0.16 | 2.90E-02 | 24990 / 284164 |
|  | 15:33788395:C:G | A/G | rs775224723 | 0.0007 | -0.545 | 0.59 | 0.27 | 0.061 |  |  |  |  |  |
|  | 15:33801900:T:C | I/T | rs150028316 | 0.0007 | 0.534 | 0.59 | 0.06 | 0.719 | 1.9 | Valvular operations | -0.21 | 2.80E-03 | 59338 / 218984 |
|  |  |  |  |  |  |  |  |  |  | Phlebitis and thrombophlebitis (not including DVT) | 0.46 | 5.10E-03 | 5135 / 267090 |
|  |  |  |  |  |  |  |  |  |  | Congenital malformations of the circulatory system | 0.57 | 7.50E-03 | 3244 / 305910 |
|  |  |  |  |  |  |  |  |  |  | Valvular heart disease excluding rheumatic fever | -0.18 | 7.90E-03 | 62218 / 218984 |
|  |  |  |  |  |  |  |  |  |  | Myocardial infarction | -0.28 | 1.10E-02 | 20404 / 260124 |
|  |  |  |  |  |  |  |  |  |  | Other heart diseases | -0.15 | 1.20E-02 | 90170 / 218984 |
|  |  |  |  |  |  |  |  |  |  | Ischemic heart diseases | -0.19 | 1.30E-02 | 49030 / 260124 |
|  |  |  |  |  |  |  |  |  |  | Conduction disorders | -0.40 | 1.50E-02 | 7262 / 218984 |
|  |  |  |  |  |  |  |  |  |  | Myocardial infarction, strict | -0.26 | 2.40E-02 | 18339 / 260124 |
|  |  |  |  |  |  |  |  |  |  | Nonpyogenic thrombosis of intracranial venous system | 1.50 | 2.50E-02 | 169 / 284164 |
|  |  |  |  |  |  |  |  |  |  | Ischaemic heart disease, wide definition | -0.17 | 3.00E-02 | 48706 / 260448 |
|  |  |  |  |  |  |  |  |  |  | Other arterial embolism and thrombosis | 1.20 | 3.20E-02 | 608 / 288638 |
|  |  |  |  |  |  |  |  |  |  | Cardiomyopathy | -0.45 | 3.70E-02 | 4606 / 218984 |
|  |  |  |  |  |  |  |  |  |  | AV-block | -0.44 | 3.90E-02 | 3968 / 218984 |
|  |  |  |  |  |  |  |  |  |  | Major coronary heart disease event excluding revascularizations | -0.19 | 4.40E-02 | 27443 / 275228 |
|  |  |  |  |  |  |  |  |  |  | Other other unspecified disorders of the circulatory system | -1.00 | 4.50E-02 | 771 / 305353 |
|  | 15:33821528:A:G | M/V | rs146201205 | 0.009 | 0.861 | 1.39E-03 | 0.71 | 0.007 | 11.3 | All-cause Heart Failure | -0.18 | 3.80E-03 | 19350 / 288996 |
|  |  |  |  |  |  |  |  |  |  | Heart failure,strict | -0.17 | 5.10E-03 | 19676 / 272371 |
|  |  |  |  |  |  |  |  |  |  | Non-ischemic cardiomyopathy | -0.23 | 1.30E-02 | 7047 / 253401 |
|  |  |  |  |  |  |  |  |  |  | AV-block | -0.25 | 4.70E-02 | 3968 / 218984 |
|  | 15:33835049:A:C | N/H | rs202181075 | 0.009 | 0.670 | 0.01 | 1 | 0.023 | 11.3 | Statin medication | 0.07 | 8.20E-03 | 100639 / 208515 |
|  |  |  |  |  |  |  |  |  |  | Diseases of veins, lymphatic vessels and lymph nodes, not elsewhere classified | 0.09 | 8.70E-03 | 42064 / 267090 |
|  |  |  |  |  |  |  |  |  |  | Coronary atherosclerosis | 0.10 | 1.30E-02 | 36418 / 260124 |
|  |  |  |  |  |  |  |  |  |  | Oesophageal varices | 0.47 | 1.40E-02 | 769 / 267090 |
|  |  |  |  |  |  |  |  |  |  | Coronary revascularization (ANGIO or CABG) | 0.13 | 1.60E-02 | 19151 / 260124 |
|  |  |  |  |  |  |  |  |  |  | Coronary angiopasty | 0.14 | 2.50E-02 | 12553 / 260124 |
|  |  |  |  |  |  |  |  |  |  | Progressive vascular leukoencephalopathy | -0.61 | 3.30E-02 | 452 / 284164 |
|  |  |  |  |  |  |  |  |  |  | Thromboangiitis obliterans | 1.40 | 3.80E-02 | 85 / 288638 |
|  |  |  |  |  |  |  |  |  |  | Rheumatic fever without mention of heart involvement | 1.40 | 4.20E-02 | 80 / 309074 |
|  |  |  |  |  |  |  |  |  |  | Coronary artery bypass grafting | 0.15 | 4.80E-02 | 8693 / 260124 |
|  | 15:33838411:T:C | M/T | rs747261018 | 0.0007 | -0.015 | 0.99 | 0.64 | 0.003 | NA |  |  |  |  |
|  | 15:33841953:G:A | R/Q | rs201791791 | 0.0013 | -0.355 | 0.62 | 0.2 | 0.174 | 0.85 | Diseases of arteries, arterioles and capillaries (FINNGEN) | 0.58 | 1.00E-02 | 16317 / 156457 |
|  |  |  |  |  |  |  |  |  |  | Hypertension, Pulmonary Arterial | 2.10 | 1.30E-02 | 179 / 223663 |
|  |  |  |  |  |  |  |  |  |  | Hypotension | 1.10 | 1.30E-02 | 3061 / 305353 |
|  |  |  |  |  |  |  |  |  |  | Other disorders of veins | -0.58 | 1.50E-02 | 9288 / 267090 |
|  |  |  |  |  |  |  |  |  |  | Cerebral atherosclerosis | 2.00 | 1.90E-02 | 221 / 284164 |
|  |  |  |  |  |  |  |  |  |  | Cerebral atherosclerosis | 1.90 | 2.20E-02 | 221 / 308933 |
|  |  |  |  |  |  |  |  |  |  | Thoracic aortic aneurysm | 0.71 | 2.30E-02 | 5881 / 288638 |
|  |  |  |  |  |  |  |  |  |  | Other CVD (FINNGEN) | 1.00 | 2.60E-02 | 3061 / 156457 |
|  |  |  |  |  |  |  |  |  |  | Atherosclerosis, excluding cerebral, coronary and PAD | 0.54 | 2.80E-02 | 11189 / 288638 |
|  |  |  |  |  |  |  |  |  |  | Aortic aneurysm | 0.66 | 3.20E-02 | 6092 / 288638 |
|  |  |  |  |  |  |  |  |  |  | Other CVD | 0.78 | 4.70E-02 | 3801 / 305353 |
|  |  |  |  |  |  |  |  |  |  | Diseases of arteries, arterioles and capillaries | 0.35 | 4.90E-02 | 20516 / 288638 |
|  | 15:33857882:G:A | E/K | rs182257230 | 0.014 | -0.049 | 0.82 | 0.03 | 0.184 | 1.29 | Death due to cardiac causes | -0.14 | 1.60E-02 | 13673 / 295481 |
|  |  |  |  |  |  |  |  |  |  | Heart failure, not strict | 0.08 | 2.30E-02 | 36783 / 272371 |
|  |  |  |  |  |  |  |  |  |  | Hypertensive Heart Disease | 0.18 | 2.90E-02 | 6348 / 223663 |
|  |  |  |  |  |  |  |  |  |  | Statin medication | 0.06 | 3.50E-02 | 100639 / 208515 |
|  |  |  |  |  |  |  |  |  |  | Hypertensive heart and/or renal disease | 0.16 | 3.90E-02 | 7222 / 223663 |
|  |  |  |  |  |  |  |  |  |  | Hypertension, essential | 0.06 | 4.00E-02 | 70651 / 223663 |
|  |  |  |  |  |  |  |  |  |  | Arterial embolism and thrombosis | -0.33 | 4.60E-02 | 1296 / 288638 |
|  |  |  |  |  |  |  |  |  |  | Secondary hypertension | 0.26 | 4.90E-02 | 2045 / 156457 |

# **Table S12: FinnDiane p**hysicians and nurses participating in the collection of the FinnDiane study subjects

| FinnDiane Study Centers | Physicians and nurses |
| --- | --- |
| Anjalankoski Health Centre | S. Koivula, T. Uggeldahl |
| Central Finland Central Hospital, Jyväskylä | T. Forslund, A. Halonen, A. Koistinen, P. Koskiaho, M. Laukkanen, J. Saltevo, M. Tiihonen |
| Central Hospital of Åland Islands, Mariehamn | M. Forsen, H. Granlund, A-C. Jonsson, B. Nyroos |
| Central Hospital of Kanta-Häme, Hämeenlinna | P. Kinnunen, A. Orvola, T. Salonen, A. Vähänen |
| Central Hospital of Länsi-Pohja, Kemi | H. Laukkanen, P. Nyländen, A. Sademies |
| Central Ostrabothnian Hospital District, Kokkola | S. Anderson, B. Asplund, U. Byskata, P. Liedes, M. Kuusela, T. Virkkala |
| City of Espoo Health Centre |  |
| Espoonlahti | A. Nikkola, E. Ritola |
| Tapiola | M. Niska, H. Saarinen |
| Samaria | E. Oukko-Ruponen, T. Virtanen |
| Viherlaakso | A. Lyytinen |
| City of Helsinki Health Centre |  |
| Puistola | H. Kari, T. Simonen |
| Suutarila | A. Kaprio, J. Kärkkäinen, B. Rantaeskola |
| Töölö | P. Kääriäinen, J. Haaga, A-L. Pietiläinen |
| City of Hyvinkää Health Centre | S. Klemetti, T. Nyandoto, E. Rontu, S. Satuli-Autere |
| City of Vantaa Health Centre |  |
| Korso | R. Toivonen, H. Virtanen |
| Länsimäki | R. Ahonen, M. Ivaska-Suomela, A. Jauhiainen |
| Martinlaakso | M. Laine, T. Pellonpää, R. Puranen |
| Myyrmäki | A. Airas, J. Laakso, K. Rautavaara |
| Rekola | M. Erola, E. Jatkola |
| Tikkurila | R. Lönnblad, A. Malm, J. Mäkelä, E. Rautamo |
| Heinola Health Centre | P. Hentunen, J. Lagerstam |
| Helsinki University Central Hospital, Department of Medicine, Division of Nephrology | A. Ahola, J. Fagerudd, M. Feodoroff, D. Gordin, O. Heikkilä, K Hietala, L. Kyllönen, J. Kytö, S. Lindh, K. Pettersson-Fernholm, M. Rosengård-Bärlund, M. Rönnback, A. Sandelin, A-R Salonen, L. Salovaara, L. Thorn, J. Tuomikangas, T. Vesisenaho, J. Wadén |
| Herttoniemi Hospital, Helsinki | V. Sipilä |
| Hospital of Lounais-Häme, Forssa | T. Kalliomäki, J. Koskelainen, R. Nikkanen, N. Savolainen, H. Sulonen, E. Valtonen |
| Iisalmi Hospital | E. Toivanen |
| Jokilaakso Hospital, Jämsä | A. Parta, I. Pirttiniemi |
| Jorvi Hospital, Helsinki University Central Hospital | S. Aranko, S. Ervasti, R. Kauppinen-Mäkelin, A. Kuusisto, T. Leppälä, K. Nikkilä, L. Pekkonen |
| Jyväskylä Health Centre, Kyllö | K. Nuorva, M. Tiihonen |
| Kainuu Central Hospital, Kajaani | S. Jokelainen, P. Kemppainen, A-M. Mankinen, M. Sankari |
| Kerava Health Centre | H. Stuckey, P. Suominen |
| Kirkkonummi Health Centre | A. Lappalainen, M. Liimatainen, J. Santaholma |
| Kivelä Hospital, Helsinki | A. Aimolahti, E. Huovinen |
| Koskela Hospital, Helsinki | V. Ilkka, M. Lehtimäki |
| Kotka Heath Centre | E. Pälikkö-Kontinen, A. Vanhanen |
| Kouvola Health Centre | E. Koskinen, T. Siitonen |
| Kuopio University Hospital | E. Huttunen, R. Ikäheimo, P. Karhapää, P. Kekäläinen, M. Laakso, T. Lakka, E. Lampainen, L. Moilanen, L. Niskanen, U. Tuovinen, I. Vauhkonen, E. Voutilainen |
| Kuusamo Health Centre | T. Kääriäinen, E. Isopoussu |
| Kuusankoski Hospital | E. Kilkki, I. Koskinen, L. Riihelä |
| Laakso Hospital, Helsinki | T. Meriläinen, P. Poukka, R. Savolainen, N. Uhlenius |
| Lahti City Hospital | A. Mäkelä, M. Tanner |
| Lapland Central Hospital, Rovaniemi | L. Hyvärinen, S. Severinkangas, T. Tulokas |
| Lappeenranta Health Centre | P. Linkola, I. Pulli |
| Lohja Hospital | T. Granlund, M. Saari, T. Salonen |
| Loimaa Health Centre | A. Mäkelä, P. Eloranta |
| Länsi-Uusimaa Hospital, Tammisaari | I-M. Jousmaa, J. Rinne |
| Malmi Hospital, Helsinki | H. Lanki, S. Moilanen, M. Tilly-Kiesi |
| Mikkeli Central Hospital | A. Gynther, R. Manninen, P. Nironen, M. Salminen, T. Vänttinen |
| Mänttä Regional Hospital | I. Pirttiniemi, A-M. Hänninen |
| North Karelian Hospital, Joensuu | U-M. Henttula, P. Kekäläinen, M. Pietarinen, A. Rissanen, M. Voutilainen |
| Nurmijärvi Health Centre | A. Burgos, K. Urtamo |
| Oulankangas Hospital, Oulainen | E. Jokelainen, P-L. Jylkkä, E. Kaarlela, J. Vuolaspuro |
| Oulu Health Centre | L. Hiltunen, R. Häkkinen, S. Keinänen-Kiukaanniemi |
| Oulu University Hospital | R. Ikäheimo |
| Päijät-Häme Central Hospital | H. Haapamäki, A. Helanterä, S. Hämäläinen, V. Ilvesmäki, H. Miettinen |
| Palokka Health Centre | P. Sopanen, L. Welling |
| Pieksämäki Hospital | V. Javtsenko, M. Tamminen |
| Pietarsaari Hospital | M-L. Holmbäck, B. Isomaa, L. Sarelin |
| Pori City Hospital | P. Ahonen, P. Merensalo, K. Sävelä |
| Porvoo Hospital | M. Kallio, B. Rask, S. Rämö |
| Raahe Hospital | A. Holma, M. Honkala, A. Tuomivaara, R. Vainionpää |
| Rauma Hospital | K. Laine, K. Saarinen, T. Salminen |
| Riihimäki Hospital | P. Aalto, E. Immonen, L. Juurinen |
| Salo Hospital | A. Alanko, J. Lapinleimu, P. Rautio, M. Virtanen |
| Satakunta Central Hospital, Pori | M. Asola, M. Juhola, P. Kunelius, M-L. Lahdenmäki, P. Pääkkönen, M. Rautavirta |
| Savonlinna Central Hospital | E. Korpi-Hyövälti, T. Latvala, E. Leijala |
| South Karelia Central Hospital, Lappeenranta | T. Ensala, E. Hussi, R. Härkönen, U. Nyholm, J. Toivanen |
| Tampere Health Centre | A. Vaden, P. Alarotu, E. Kujansuu, H. Kirkkopelto-Jokinen, M. Helin, S. Gummerus, L. Calonius, T. Niskanen, T. Kaitala, T. Vatanen |
| Tampere University Hospital | I. Ala-Houhala, T. Kuningas, P. Lampinen, M. Määttä, H. Oksala, T. Oksanen, K. Salonen, H. Tauriainen, S. Tulokas |
| Tiirismaa Health Centre, Hollola | T. Kivelä, L, Petlin, L. Savolainen |
| Turku Health Centre | I. Hämäläinen, H. Virtamo, M. Vähätalo |
| Turku University Central Hospital | K. Breitholz, R. Eskola, K. Metsärinne, U. Pietilä, P. Saarinen, R. Tuominen, S. Äyräpää |
| Vaajakoski Health Centre | K. Mäkinen, P. Sopanen |
| Valkeakoski Regional Hospital | S. Ojanen, E. Valtonen, H. Ylönen, M. Rautiainen, T. Immonen |
| Vammala Regional Hospital | I. Isomäki, R. Kroneld, M. Tapiolinna-Mäkelä |
| Vaasa Central Hospital | S. Bergkulla, U. Hautamäki, V-A. Myllyniemi, I. Rusk |

Fig. S1: Power calculations. A: Statistical power to detect a single-variant association with exome-wide significant p<4.3×10-7 for the standard lipid measurements with ~920 samples. B: Statistical power to detect a single-variant association with exome-wide significant p<4.3×10-7 for the serum NMR lipid measurements with 748 samples. C, D: Simplistic statistical power calculations to detect an aggregate association based on the cumulative minor allele count (MAC) of five variants with a given MAF, to yield a significant p-value after correction for the number of genes with protein altering variants, p<2.9×10-6, for standard lipid measurements (C) or serum NMR lipids (D).


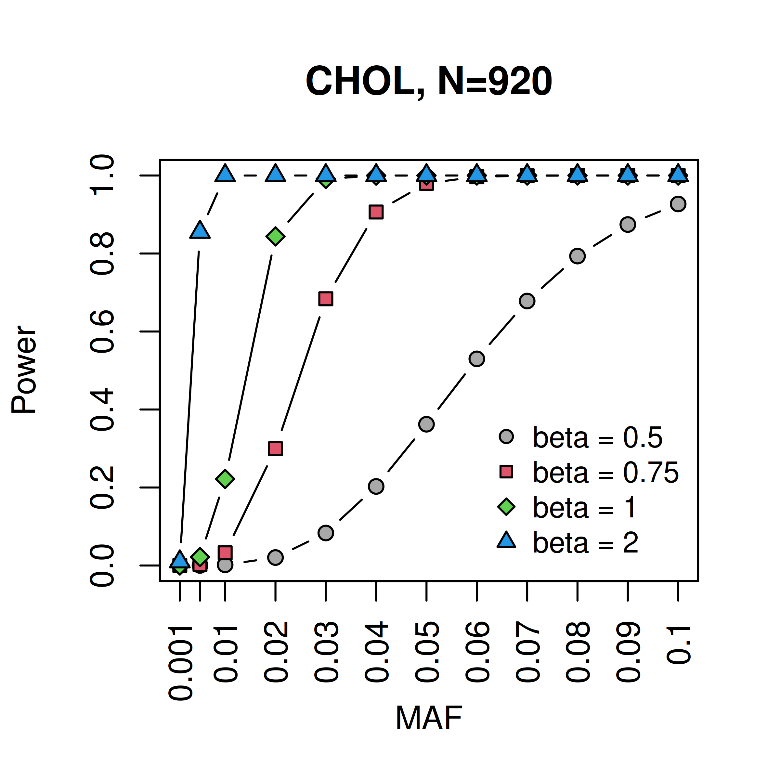

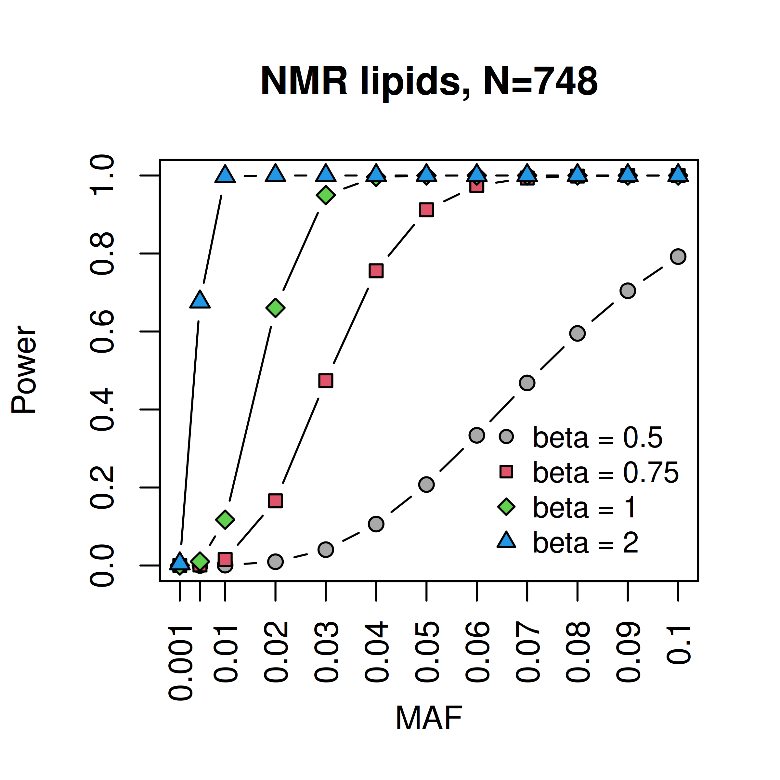


A

B


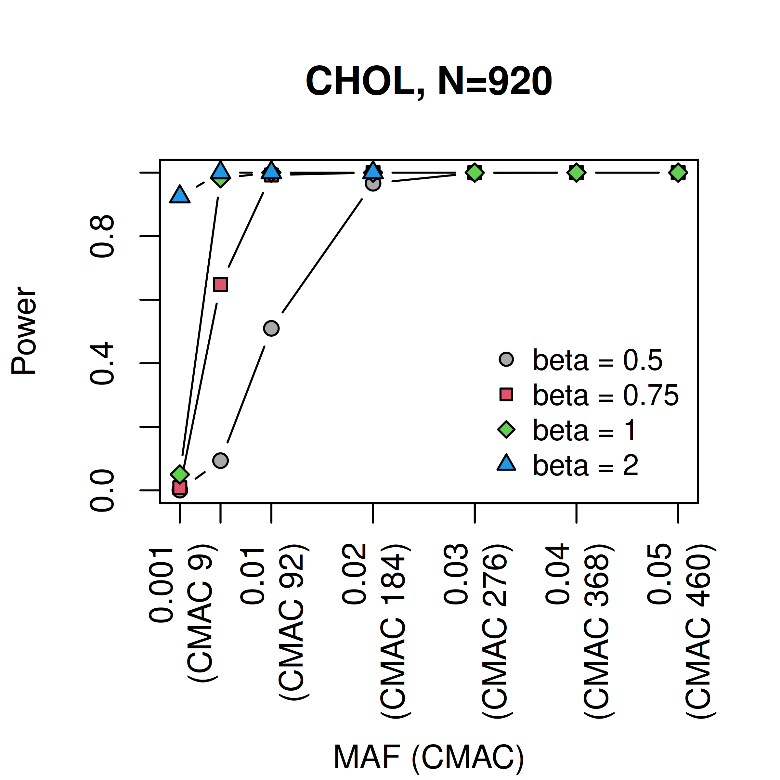

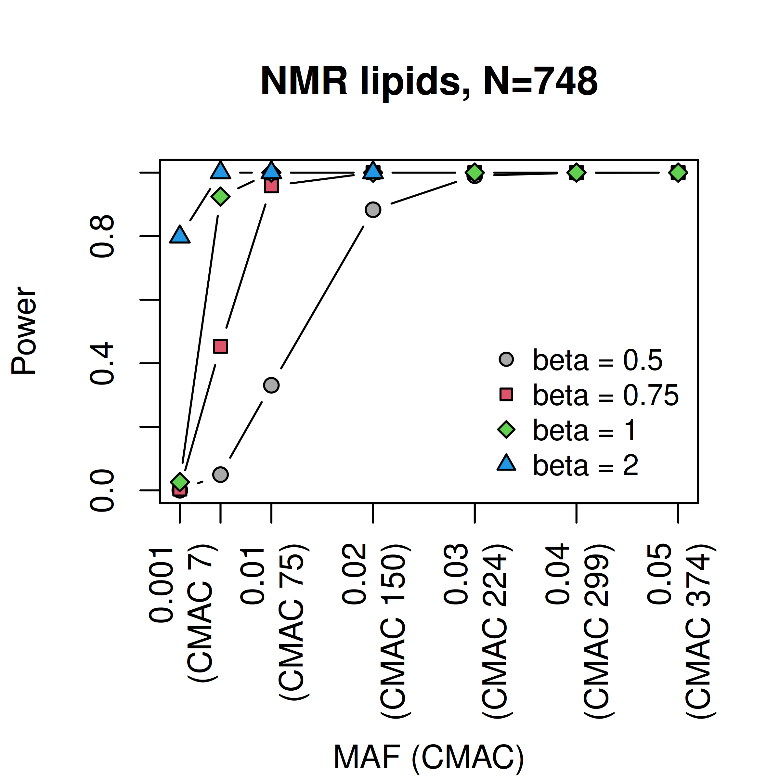


D

C

Fig. S2: Correlation plot of analyzed lipid phenotypes. Correlations were calculated with R 4.2.0 and drawn with the corrplot v0.92 package. The lipid phenotypes were ordered by hierarchical clustering.


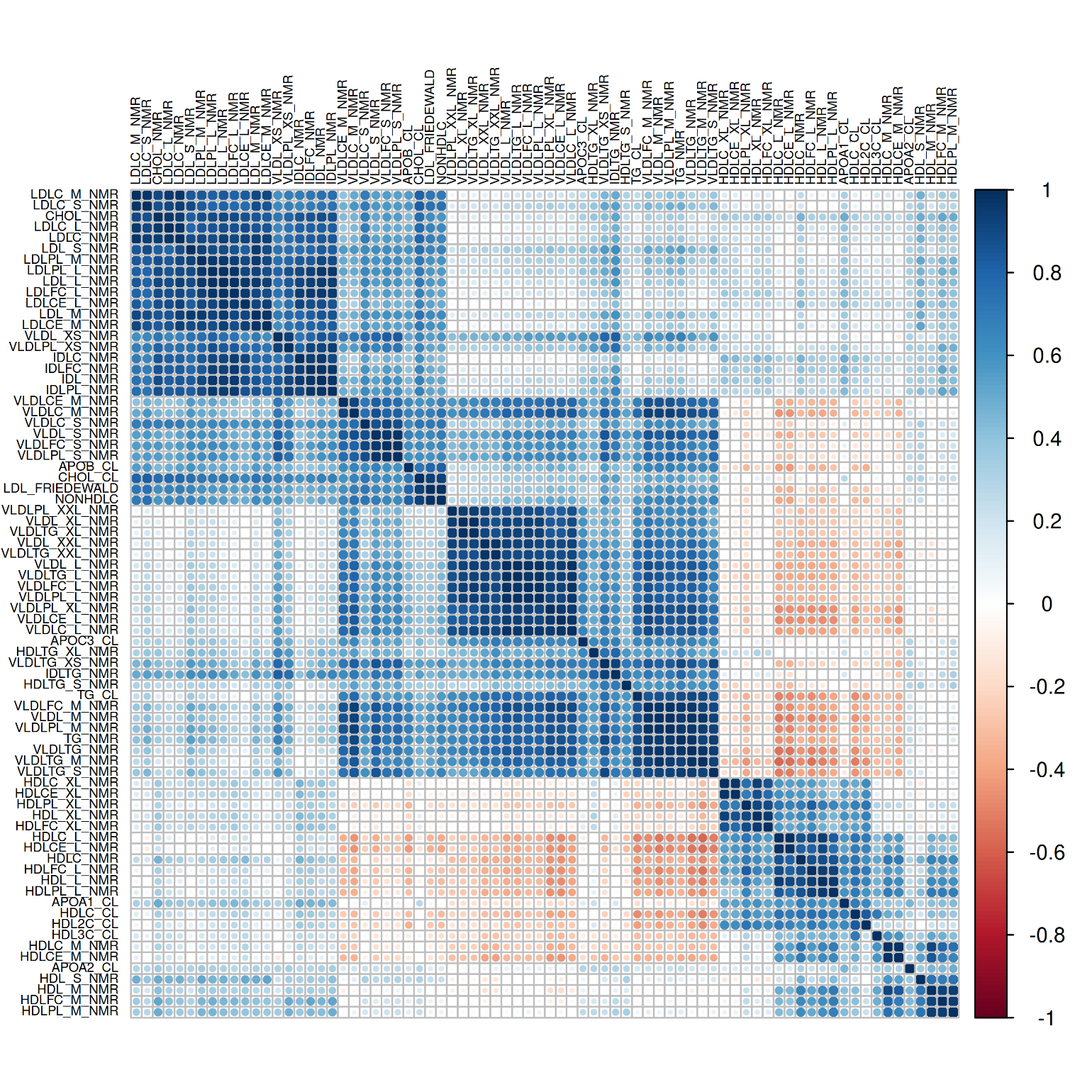


# **Fig. S3: Survival analysis for coronary artery disease (CAD) and strokes for the *LIPC*** p.Thr405Met **and *RBM47*** p.Ala496-Ala502del variants.

**A and B:** Kaplan-Meier survival plot of CAD events (**A**) and strokes (**B**), stratified by *LIPC* p.Thr405Met (rs113298164) variant count. Cox proportional hazard (PH) model *p*-value was non-significant for both CAD and strokes. **C and D:** Kaplan-Meier survival plot of CAD events (**C**) and strokes (**D**), stratified by *RBM47* p.Ala496-Ala502del (rs564837143) variant count (Cox PH models non-significant). The analysis had 35% power to detect association with CAD assuming a hazard ratio [HR] of 1.5 for the *LIPC* lead variant with allele frequency of 1.7%, median follow-up time of 56 years, and event rate of 33% (obtained from the data). Corresponding analysis indicated 19% power for the *RBM47* (MAF 0.008%) variant.


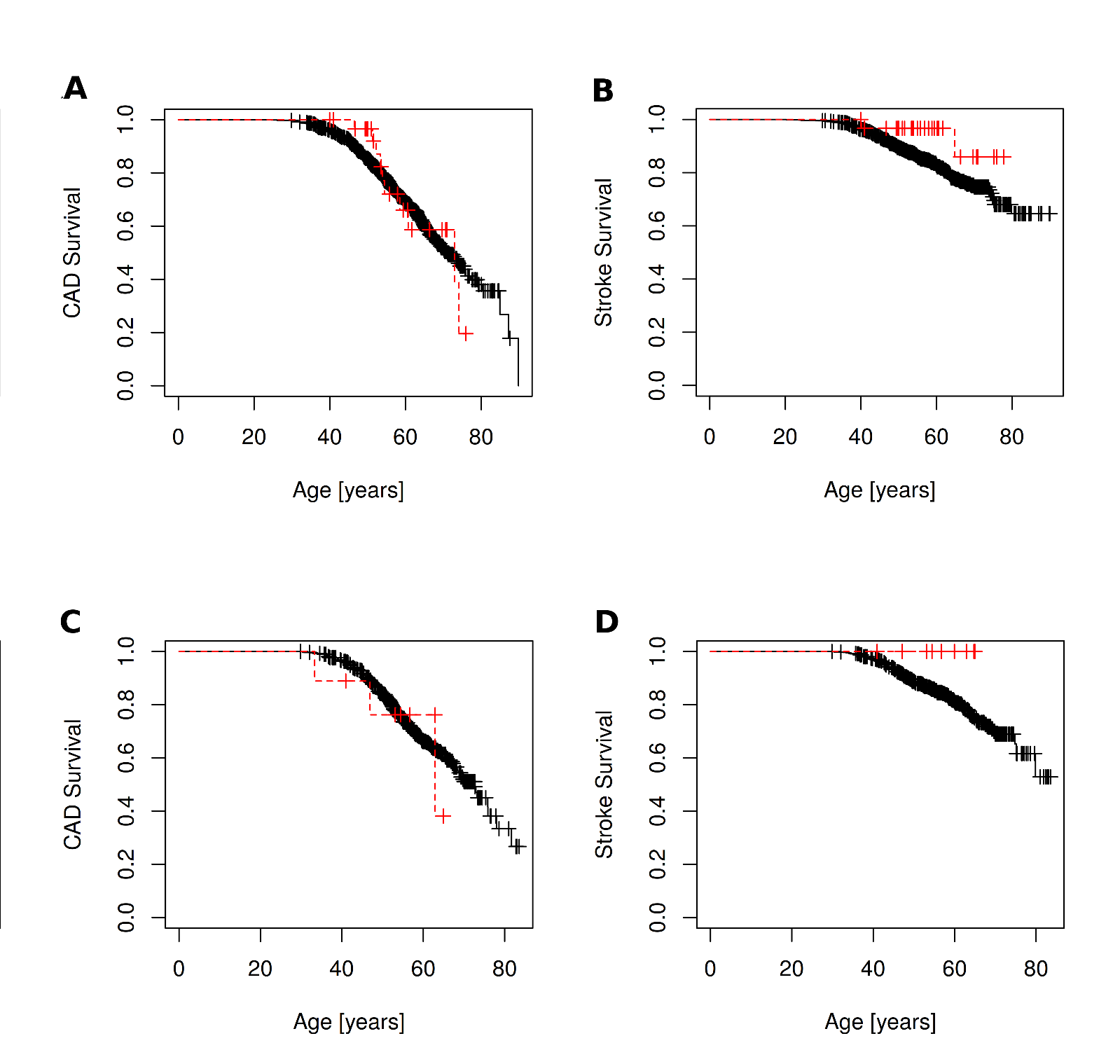


Fig. S4: PAVs in *CYP3A43* are associated with lower cholesterol esters in large LDL particles. **A:** *CYP3A43* PAVs are associated with lower cholesterol esters in large LDL particularly among individuals with statin medication. **B:** Four PAVs with MAF < 5% were identified among individuals with statin medication. All p.Pro340Ala carriers also had the p.Tyr25fs variant.

# Fig. S5: WES+WGS SKAT meta-analysis results, Manhattan and QQ-plots**.**

A: PAVs for total cholesterol. B: PAVs for apo-A1. C: PAVs for apoC-III. D: PAVs for phospholipids in extra small VLDL particles. E: PTVs for phospholipids in extra large VLDL particles.

A

B

C

D

E

Fig. S6: *APOB* rs1407451223 (N=2) or rs1232943044 (N=1) protein truncating frameshift variants are associated with non-HDL and LDL cholesterol and Apolipoprotein B (apoB) concentrations. A: Non-HDLC concentration in the PTV carriers (group 1: 1.7 – 3.2 mmol/L) vs. in the non-carriers (group 0; median 3.9 mmol/L, interquartile range 3.2 – 4.7 mmol/L). B: Apolipoprotein B (apoB) concentration in the three APOB frameshift carriers (group 1; 44 – 50 mg/dl) vs. in the other participants (group 0; median 88 mg/dl, interquartile range 72 – 104 mg/dl). C: LDL cholesterol (LDLC) concentration in the three APOB frameshift carriers (group 1; 1.6 – 3.1 mmol/L) vs. in the other participants (group 0; median 3.6 mmol/L, interquartile range 3.0 – 4.4 mmol/L).

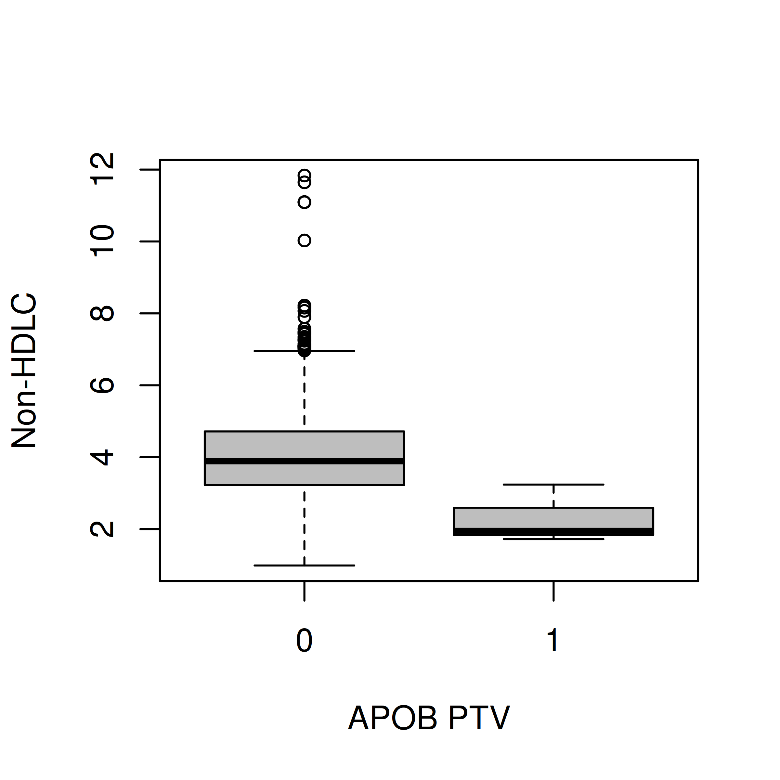

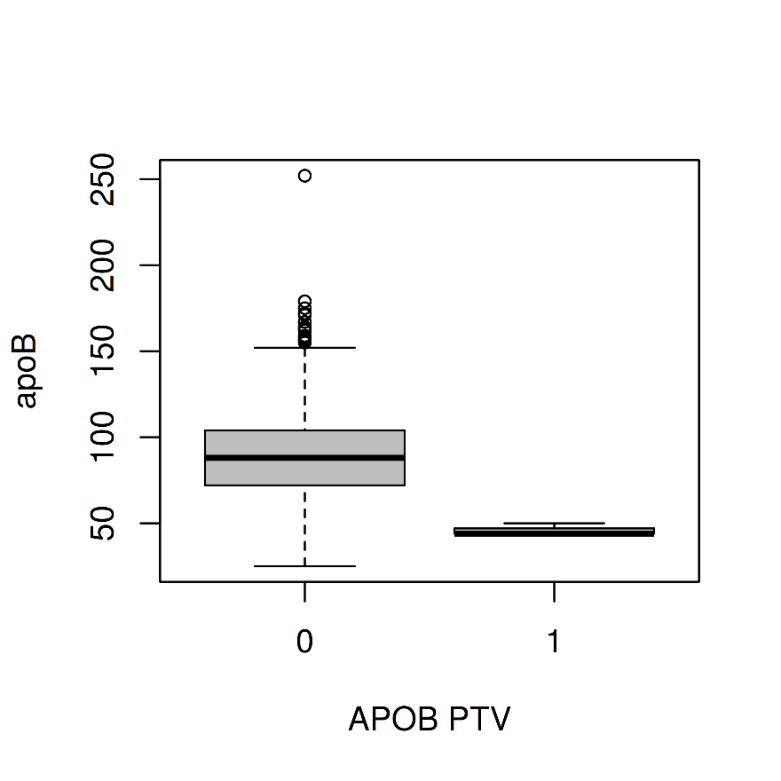


B

A


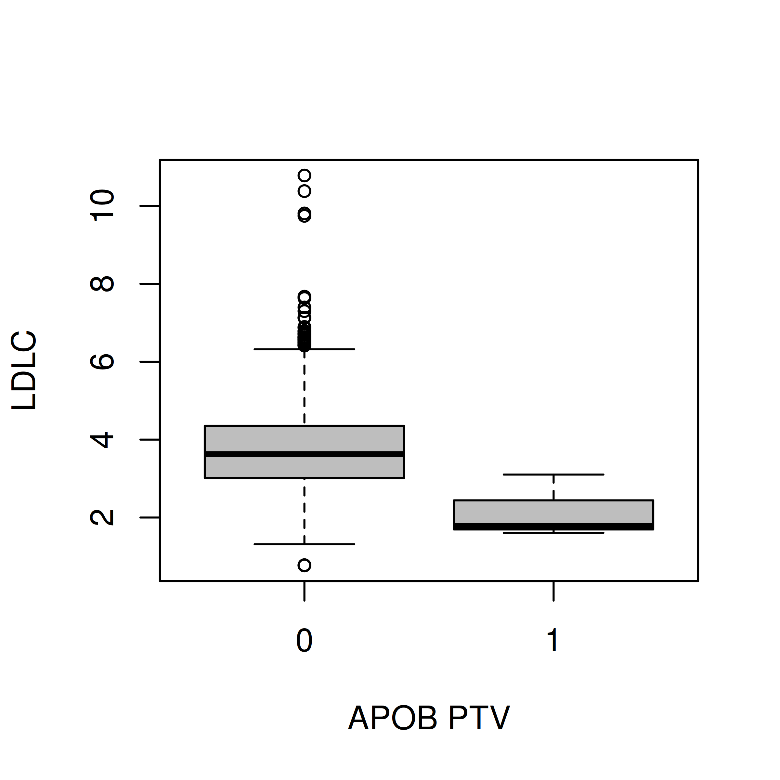


C

Fig. S7: WES-WGS SKAT meta-analysis results for genes previously associated with rare lipid disorders. **A:** Enrichment of PAVs. **B:** Enrichment of PTVs. The color indicates the strength of the gene – phenotype association (SKAT p-value).


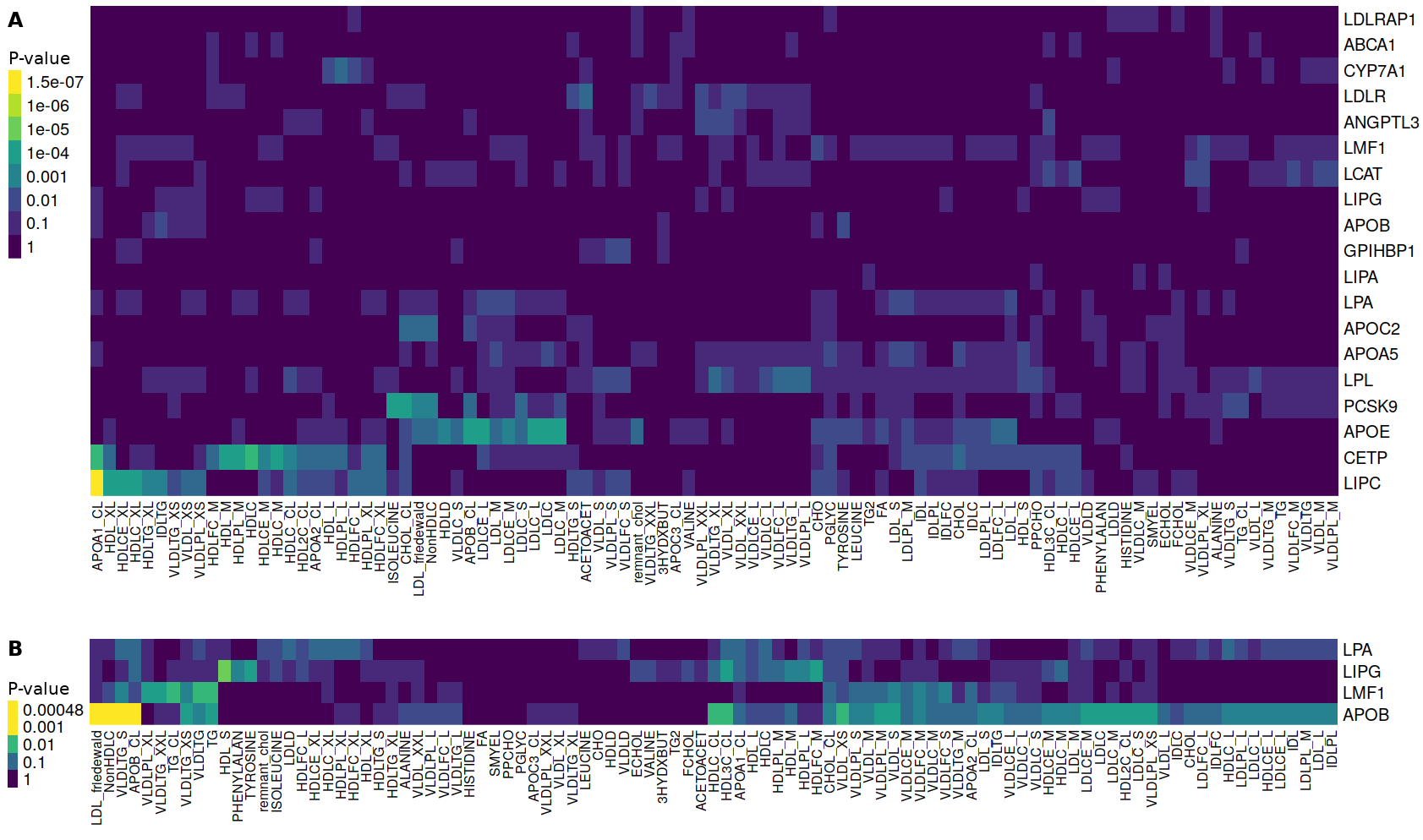


# References

Backman, Joshua D., Alexander H. Li, Anthony Marcketta, Dylan Sun, Joelle Mbatchou, Michael D. Kessler, Christian Benner, et al. 2021. “Exome Sequencing and Analysis of 454,787 UK Biobank Participants.” *Nature* 599 (7886): 628–34. https://doi.org/10.1038/s41586-021-04103-z.

Friedewald WT, Levy RI, Fredrickson DS. Estimation of the concentration of low-density lipoprotein cholesterol in plasma, without use of the preparative ultracentrifuge. Clin Chem 1972;18:499-502.

Graham, Sarah E., Shoa L. Clarke, Kuan-Han H. Wu, Stavroula Kanoni, Greg J. M. Zajac, Shweta Ramdas, Ida Surakka, et al. 2021. “The Power of Genetic Diversity in Genome-Wide Association Studies of Lipids.” *Nature* 600 (7890): 675–79. https://doi.org/10.1038/s41586-021-04064-3.

Hindy, George, Peter Dornbos, Mark D. Chaffin, Dajiang J. Liu, Minxian Wang, Margaret Sunitha Selvaraj, David Zhang, et al. 2022. “Rare Coding Variants in 35 Genes Associate with Circulating Lipid Levels-A Multi-Ancestry Analysis of 170,000 Exomes.” *American Journal of Human Genetics* 109 (1): 81–96. https://doi.org/10.1016/j.ajhg.2021.11.021.

Kopanos C, Tsiolkas V, Kouris A, Chapple CE, Albarca Aguilera M, Meyer R, Massouras A. VarSome: the human genomic variant search engine. Bioinformatics 2019;35:1978-1980.

Musunuru K. Dyslipidemias: Pathophysiology, Evaluation and Management: . NJ: Totowa, 2015.

Richards S, Aziz N, Bale S, Bick D, Das S, Gastier-Foster J, Grody WW, Hegde M, Lyon E, Spector E, Voelkerding K, Rehm HL, on behalf of the ACMG Laboratory Quality, Assurance Committee. Standards and guidelines for the interpretation of sequence variants: a joint consensus recommendation of the American College of Medical Genetics and Genomics and the Association for Molecular Pathology. Genetics in Medicine 2015;17:405-423

Tolonen N, Forsblom C, Thorn L, Wadén J, Rosengård-Bärlund M, Saraheimo M, Heikkilä O, Pettersson-Fernholm K, Taskinen M-, Groop P-, FinnDiane SG. Relationship between lipid profiles and kidney function in patients with type 1 diabetes. Diabetologia 2008;51:12-20.

Varbo A, Benn M, Nordestgaard BG. Remnant cholesterol as a cause of ischemic heart disease: Evidence, definition, measurement, atherogenicity, high risk patients, and present and future treatment. Pharmacol Ther 2014;141:358-367.

# Text S1: Example code to run WES/WGS meta-analysis of the coding variants

### for single variant and gene aggregate tests

### 2022-10-27 N Sandholm and R Hotakainen

variants="'chromosome_number_variation|exon_loss_variant|frameshift_variant|stop_gained|stop_lost|start_lost|splice_acceptor_variant|splice_donor_variant|missense_variant|inframe_insertion|disruptive_inframe_insertion|inframe_deletion|disruptive_inframe_deletion'" #all protein altering variants as annotated in the WES/WGS files with SnpEff

${WESfile}="path_to_WES.vcf.gz"

${WGSfile}="path_to_WGS.vcf.gz"

${WESphenopath}="path_to_WES_phenotype_table.txt"

${WGSphenopath}="path_to_WGS_phenotype_table.txt"

sub[1]="S_APOA1_CL" ## phenotypes to be tested

s=1 # Nth phenotype to be tested

### 1. Run WES and WGS single variant analysis

###---------------------------------------------------------------------

### WES single variant analysis

rvtest \

--inVcf ${WESfile} \

--pheno ${WESphenopath} \

--pheno-name ${sub[$s]} \

--covar ${WESphenopath} \

--covar-name AGE,SEX,PC1,PC2,BATCH \

--meta score,cov[windowSize=500000] \

--freqLower 0.0005 \

--inverseNormal \

--useResidualAsPhenotype \

--annoType $variants \

--out results_WES_${sub[$s]}

#freqLower 0.0005 to include only MAC>=1

### WGS single variant analysis

rvtest \

--inVcf ${WGSfile} \

--pheno ${WGSphenopath} \

--pheno-name ${sub[$s]} \

--covar ${WGSphenopath} \

--covar-name AGE,SEX,PC1,PC2,BATCH \

--meta score,cov[windowSize=500000] \

--freqLower 0.0005 \

--inverseNormal \

--useResidualAsPhenotype \

--annoType $variants \

--out results_WGS_${sub[$s]}

#freqLower 0.0005 to include only MAC>=1

### 3. Add file locations to summary files

###---------------------------------------------------------------------

echo results_WES_${sub[$s]}.MetaScore.assoc.gz > summaryFiles_${sub[$s]}

echo results_WGS_${sub[$s]}.MetaScore.assoc.gz >> summaryFiles_${sub[$s]}

echo results_WES_${sub[$s]}.MetaCov.assoc.gz > summaryCOVs_${sub[$s]}

echo results_WGS_${sub[$s]}.MetaCov.assoc.gz >> summaryCOVs_${sub[$s]}

### 4. Write common list of WES/WGS variants

###---------------------------------------------------------------------

raremetal \

--summaryFiles summaryFiles_${sub[$s]} \

--covFiles summaryCOVs_${sub[$s]} \

--writeVcf \

--prefix variants_meta_${sub[$s]}

## Output: ${outpath}Variants/variants_meta_*.pooled.variants.vcf

### 5. Annotate WES/WGS variants

###---------------------------------------------------------------------

### remove 'chr' from the variant positions to match the WES/WGS data

sed 's/>chr/>/g' refGenomes/hg38.fa > refGenomes/hg38_wochr.fa

cd refGenomes/

# index the new fasta file

samtools faidx hg38_wochr.fa

cd ../

EPACTS-3.2.3/bin/anno \

-i variants_meta_${sub[$s]}.pooled.variants.vcf \

-o variants_meta_${sub[$s]}.pooled.variants.ANNO.vcf \

-r refGenomes/hg38_wochr.fa \

-g refGenomes/refFlat_hg38_chr_prefix_removed.txt.gz \

-p refGenomes/priority.txt \

-c refGenomes/codon.txt

### 6. Run WES-WGS meta-analysis

###--------------------------------------------------------------------------

raremetal \

--summaryFiles summaryFiles_${sub[$s]} \

--covFiles summaryCOVs_${sub[$s]} \

--annotatedVcf variants_meta_${sub[$s]}.pooled.variants.ANNO.vcf \

--annotation Start_Loss/Start_Gain/Stop_Loss/Stop_Gain/Frameshift/Essential_Splice_Site/StructuralVariation/CodonLoss/CodonGain/Nonsynonymous \

--SKAT \

--VT \

--maf 0.05 \

--prefix meta_analysis_${sub[$s]}_0.05_PAV

raremetal \

--summaryFiles summaryFiles_${sub[$s]} \

--covFiles summaryCOVs_${sub[$s]} \

--annotatedVcf variants_meta_${sub[$s]}.pooled.variants.ANNO.vcf \

--annotation Start_Loss/Start_Gain/Stop_Loss/Stop_Gain/Frameshift/Essential_Splice_Site/ \

--SKAT \

--VT \

--maf 0.05 \

--prefix meta_analysis_${sub[$s]}_0.05_PTV
